# Controlled human malaria infections by mosquito bites induce more severe clinical symptoms than asexual blood-stage challenge infections

**DOI:** 10.1016/j.ebiom.2022.103919

**Published:** 2022-03-09

**Authors:** Manon Alkema, X. Zen Yap, Gerdie M. de Jong, Isaie J. Reuling, Quirijn de Mast, Reinout van Crevel, Christian F. Ockenhouse, Katharine A. Collins, Teun Bousema, Matthew B.B. McCall, Robert W. Sauerwein

**Affiliations:** a Department of Medical Microbiology, Radboud Center for Infectious Diseases, Radboud university medical center, 6500 HB Nijmegen, The Netherlands; b Department of Medical Microbiology and Infectious Diseases, University Medical Center Rotterdam, 3000 CA Rotterdam, The Netherlands; c Department of Internal Medicine, Radboud Center for Infectious Diseases, Radboud university medical center, 6500 HB Nijmegen, The Netherlands; d PATH Malaria Vaccine Initiative, Washington, DC 20001, United States

**Keywords:** Malaria, Plasmodium falciparum, Inflammation, Controlled Human Malaria Infection, Tolerance

## Abstract

**Background:**

Fever and inflammation are a hallmark of clinical *Plasmodium falciparum (Pf)* malaria induced by circulating asexual parasites. Although clinical manifestations of inflammation are associated with parasite density, this relationship is influenced by a complex network of immune-modulating factors of both human and parasite origin.

**Methods:**

In the Controlled Human Malaria infection (CHMI) model, we compared clinical inflammation in healthy malaria-naïve volunteers infected by either *Pf*-infected mosquito bites (MB, n=12) or intravenous administration of *Pf*-infected red blood cells (BS, n=12).

**Findings:**

All volunteers developed patent parasitaemia, but both the incidence and duration of severe adverse events were significantly higher after MB infection. Similarly, clinical laboratory markers of inflammation were significantly increased in the MB-group, as well as serum pro-inflammatory cytokine concentrations including IFN-γ, IL-6, MCP1 and IL-8. Parasite load, as reflected by maximum parasite density and area under the curve, was similar, but median duration of parasitaemia until treatment was longer in the BS-group compared to the MB-group (8 days [range 8 – 8 days] versus 5·5 days [range 3·5 – 12·5 days]). The *in vitro* response of subsets of peripheral blood mononuclear cells showed attenuated *Pf*-specific IFNγ production by γδ T-cells in the BS-arm.

**Interpretation:**

In conclusion, irrespective the parasite load, *Pf*-infections by MB induce stronger signs and symptoms of inflammation compared to CHMI by BS infection. The pathophysiological basis remains speculative but may relate to induced immune tolerance.

**Funding:**

The trial was supported by PATH's Malaria Vaccine Initiative; the current analyses were supported by the AMMODO Science Award 2019 (TB).

**Research in context**

**Evidence before this study**

We conducted an online literature search on inflammation or severity of symptomatology following human malaria infection using PubMed for articles published up to October 1st, 2021. Articles were searched using the terms "Malaria"[Mesh] AND ("Virulence"[Mesh] OR "Inflammation"[Mesh] OR "disease severity" OR "tolerability") AND ("blood stage" OR "blood stage infection" OR "blood passage" OR "induced blood stage malaria" OR "mosquito bite" OR mosquito bite infection OR "controlled human malaria infection") and screened on title and abstract. Of the 397 screened articles, 15 contained information on inflammation or disease severity following mosquito bite or blood-stage infection in humans specifically. None of the articles included a side-by-side analysis of symptoms or signs of inflammation in humans following infection by mosquito bite and blood-stage infection. Some articles referenced in the screened publications were also included, but contained mostly information on non-falciparum strains in animal models. These studies were mutually contradictive and provide no clear leads for pathophysiologic mechanisms underlying a difference in disease severity resulting from different infection routes.

**Added value of this study**

Here, we directly compare clinical inflammatory reactions to controlled human malaria infection (CHMI) commencing at two distinct *Pf* stages; sporozoite and blood-stage. This study adds new insights into the pathophysiology of malaria. Our combined findings suggest that exposure to initially low densities of blood-stage parasites induces a level of tolerance towards *Pf* parasites, leading to significantly milder symptomatology and laboratory abnormalities following blood-stage infection compared to infection by mosquito bites.

**Implications of all the available evidence**

Recognising and understanding this phenomenon is of significant importance for the understanding of general malaria pathophysiology and parasite-host interaction. Clinical trials using the controlled human malaria infection model should carefully consider the implications of the chosen infection route on the clinical symptoms and inflammation parameters.

Alt-text: Unlabelled box

## Introduction

*Plasmodium falciparum* (*Pf*) infection is characterised clinically by marked systemic inflammation that is generally associated with circulating asexual parasite density. However, even in malaria-naïve individuals without pre-existing immunity, presentation and severity of clinical symptoms following *Pf* infection can be heterogeneous due to modulation of host immunity.

We recently conducted a controlled human malaria infection (CHMI) in malaria-naïve Dutch volunteers with the aim of generating *Pf* gametocytes, the life-cycle stage responsible for malaria transmission from the human host to the mosquito vector. In order to achieve a predefined density of asexual parasitaemia acting as source of these gametocytes, volunteers were subjected to distinct infection regimens; volunteers received either mosquito bites from laboratory-reared *Pf*-infected mosquitoes (MB-group) or intravenous inoculation of asexual blood-stage parasites (BS-group) from an established master cell bank.^1^^,^^2^

Here we report on the contrasting clinical parameters and signs of systemic inflammation in these two groups, which were monitored by number, severity and duration of adverse events, as well as markers of inflammation in the circulation and in peripheral blood mononuclear cells (PBMC) after *in vitro* stimulation.

## Methods

### Ethics

An open-label, single centre, randomised, trial was conducted at the Radboud university medical center (Nijmegen, the Netherlands) as described before.^3^ The trial protocol (file number NL63552.000.17) was approved by the Dutch Central Committee for Research involving Human subjects (CCMO), the Western Institutional Review Board (WIRB), and registered at ClinicalTrials.gov, identifier NCT03454048 and EudraCT, identifier 2017-00040005-40.

### Study design

In short, twenty-four healthy, malaria naïve volunteers (18 – 35 years) provided informed consent and were screened and enrolled to receive infection by either bites of 5 mosquitoes infected with the *Pf* 3D7 isolate, or by intravenous injection of ∼2,800 *Pf* 3D7 infected red blood cells. Volunteers in the MB-group received a sub-curative treatment of piperaquine (480mg) when parasitaemia reached 5,000 parasites/mL as defined by 18S qPCR, or at an earlier timepoint if pre-defined safety criteria were met. All volunteers of the BS-group received sub-curative treatment of piperaquine (480mg) on day 8 post-infection based on earlier findings ^2^, or at an earlier timepoint if parasitaemia reached 5,000 parasites/mL before day 8 and was accompanied with clinical symptoms of malaria. All volunteers received a final curative treatment between day 27 – 36 according to protocol.^3^

Original primary and secondary study outcomes focussed on safety and transmissibility to mosquitoes as previously described in detail.^3^

Adverse events were recorded from inclusion until day 51 post-infection, and graded for severity; grade 1 (mild) defined as: awareness of symptoms that are easily tolerated and do not interfere with usual daily activity; grade 2 (moderate) defined as discomfort that interferes with or limits usual daily activity; and grade 3 (severe) defined as disabling, with subsequent inability to perform usual daily activity, resulting in absenteeism or requiring bed rest. For each AE a start and stop date and time was recorded to calculate its duration. Relatedness to the trial procedures (CHMI and drug treatment) was categorised for each adverse event as not related, possibly related or probably related. Only adverse events that were possibly or probably related to the trial were included in the current analysis. Local adverse events at the site of injection or mosquito bite were recorded but not included in these analyses.

Clinical laboratory parameters including complete blood count, C-reactive protein, liver enzymes (AST, ALT, ALP, bilirubin and gammaGT) and troponin-T were determined at predefined timepoints during the trial. Laboratory normal values are represented in supplemental table 1. Parasitaemia was quantified by 18S qPCR as described before.^4^

### PBMC and serum collection

**Figure 1:**
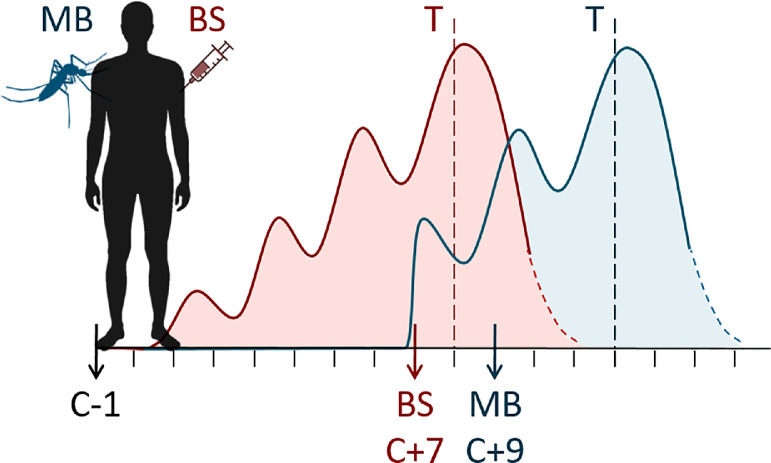
Schematic study design and course of parasitaemia. Malaria naïve volunteers were challenged by either bites of 5 infected mosquitoes or by injection with ∼2,800 infected red blood cells, both with the *Pf* 3D7 isolate. Following mosquito bite, *Pf* sporozoites first invade liver cells, within which they undergo schizogony over the course of ∼7 days before releasing a first wave of blood-stage parasites into circulation, which subsequently commence cyclical asexual blood-stage multiplication (blue curve). Following inoculation of infected red blood cells, in contrast, blood-stage multiplication commences immediately (red curve). Both curves represent schematic blood-stage parasite density in peripheral venous blood on a log-scale; the apparent wave-like pattern of parasitaemia observed in peripheral blood is due to sequential sequestration of mature blood-stage schizonts in the vasculature and subsequent release of next-generation parasites into peripheral circulation in the course of each multiplication cycle. Volunteers infected by mosquito bite (MB, n=12) received treatment (T, dashed lines) when parasitaemia reached 5,000 parasites/mL, or earlier if safety criteria were met. One subject received treatment at day 10•3 due to thrombocytopenia; this subject was included in all analyses. Volunteers who received a blood stage inoculum (BS, n=12) were all treated presumptively on day 8 post-challenge. PBMC sampling time points are indicated with arrows and days relative to challenge infection (C+#).

PBMCs and sera were collected and stored as previously described.^5^ Briefly, venous whole blood was collected in CPT vacutainers containing citrate (BD Biosciences) at key timepoints (Figure 1), PBMCs were isolated and cryopreserved. For cytokine analyses, venous whole blood was collected in SSTII Advance vacutainers (BD Biosciences), blood was centrifuged and sera were cryopreserved.

### Cytokine analysis

Inflammatory cytokines (IL-1β, IFN-α, IFN-γ, TNF-α, MCP-1, IL-6, IL-8, IL-10, IL-12p70, IL-17A, IL-18, IL-23, and IL-33) were determined in serum prior to challenge infection (C-1), 7 or 9 days after infection (C+7 for BS and C+9 for MB), at time of treatment (T), as well as two (T+2) and four (T+4) days thereafter, using a flow-based multiplex kit (Legendplex, Cat# 740809) measured on a Beckman-Coulter Gallios flow cytometer.

### PBMC stimulation and flow cytometry

Cryopreserved PBMCs collected at baseline and prior to treatment (C+7 for BS and C+9 for MB, respectively) were thawed, counted using a haemocytometer and 0·1% Trypan Blue, and resuspended in culture media consisting of Dutch modified Roswell Park Memorial Institute (RPMI) 1640 (Gibco, Cat# 11875101) media supplemented with 2mM Glutamax (Gibco, Cat# 35050061), 0·05M gentamycin, 1mM pyruvate, and 10% A^+^ human serum (Sanquin, Nijmegen) and plated at a concentration of 0·5 × 10^6^ PBMCs/well in 96-well U-bottom plates. PBMCs from each volunteer were stimulated in duplicate with 1 × 10^6^ NF54 parasitised red blood cells (*Pf*RBC) per well for a ratio of 2 *Pf*RBC:PBMC for 24h at 37°C in 5% CO_2_. PBMCs were also stimulated with 1 × 10^6^ uninfected RBCs (uRBCs) per well as a control. CD107a-Pacific Blue (BioLegend Cat# 328624, RRID:AB_2265606) monoclonal antibody was added for the duration of the stimulation period. For the last 4 h of stimulation, 10µg/mL Brefeldin A (Sigma-Aldrich, Cat# 20350-15-6) and 2µM monensin (eBioscience, Cat# 00-4505-51) were added to each well. PBMCs were stained with fixable viability dye eFluor 780 (eBioscience, #65-0865-14) and subsequently with: CD14-FITC (BioLegend Cat# 325604, RRID:AB_830677), pan-γδ T cell receptor-PE (BioLegend Cat# 331209, RRID:AB_1089219), CD69-PerCP-Cy5.5 (BioLegend Cat# 310926, RRID:AB_2074956), HLA-DR-PECy7 (BioLegend Cat# 307616, RRID:AB_493588), CD86-APC (BioLegend Cat# 305412, RRID:AB_493231), CD56-AF700 (BioLegend Cat# 318316, RRID:AB_604104), CD3-PB (BioLegend Cat# 317314, RRID:AB_571909) and CD16-BrilliantViolet510 (BioLegend Cat# 302242, RRID:AB_2561668). Cells were permeabilised using FoxP3 Fix/Perm kit (Thermo Fisher, Cat# 00-5521-00) and then stained with IFN-γ- PE-DazzleCF594 (BioLegend Cat# 502545, RRID:AB_2563626) and resuspended in 1% paraformaldehyde in PBS. All samples were measured on a 10-colour BC Gallios (Beckman Coulter) using Kaluza 4.0 (Beckman Coulter). Uninfected RBC background measurements for each volunteer were subtracted from the corresponding *Pf*RBC measurement.

### Statistics

Analyses were performed using SPSS, Flowjo and Graphpad Prism 5. The Mann-Whitney U test was used for comparison of continuous variables between study arms; for dichotomous variables the Fisher's exact test was used. For comparison of variables at different timepoints within groups, the Wilcoxon signed-rank test was used. The area under the curve of asexual parasite density over time (AUC) was computed with the (ΔX)*(Y1 +Y2)/2 formula in GraphPad Prism 5. Cytokine concentrations were calculated using Graphpad Prism 5 using a 5 parameter logistic curve with log-transformed data and 12-point standard curves for each cytokine standard. Sample sizes were based on the primary objectives of the trial and allowed the detection of a proportion of infectious individuals of 34% or higher.^3^

### Role of funding source

PATH's Malaria Vaccine Initiative supported the original trial, was involved in the trial design and approved the final manuscript. AMMODO science award had no role in the design, conduct or analysis of the trial.

## Results

### Randomisation

Randomisation resulted in balanced groups as demonstrated by comparable baseline characteristics as shown previously.^3^ All 24 participants completed follow up and were included in all analyses.

### Kinetics of parasitaemia

**Figure 2:**
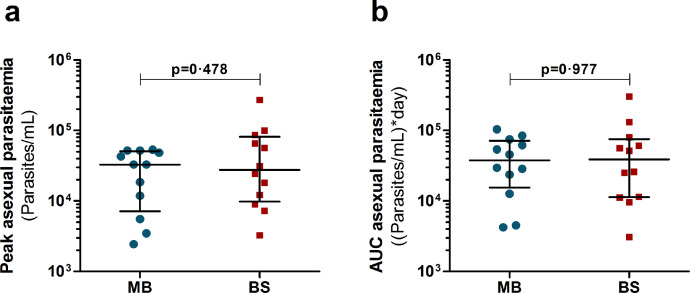
Asexual parasitaemia per study group. a) Peak asexual parasite density in mosquito bite group (MB, n=12, blue circles) and blood stage-infected subjects (BS, n=12, red squares). b) Area under the curve (AUC) asexual parasite density over time. Error bars present median with interquartile range. P-values by Mann-Whitney U test.

All twenty-four participants developed parasitaemia; MB-infected participants received treatment a median of 12·3 days post-infection (range 10·3 – 19·5) while all BS-infected participants were treated on day 8 post-infection. The duration of detectable parasitaemia was shorter in the MB-group (median of 5·3 days, range 3·3 – 12·5) compared to the BS-group (8·0 days) (shown schematically in Figure 1). One participant in the MB-group received treatment at day 10·3 due to thrombocytopaenia (105 *10^9^/L). All other subjects in the MB-group received treatment based on the predefined treatment threshold of >5,000 parasites/mL. Median peak parasitaemia, generally observed half a day after start of treatment, was 32,807 parasites/mL (interquartile range [IQR] 7,137 – 50,831 parasites/mL) for the MB-group and 27,700 (IQR 9,818 – 81,091 parasites*/*mL) for the BS-group. Median area under the parasitaemia curve (AUC) for MB was 37,654 (IQR 15,430 – 71,484) and for BS 38,735 (IQR 11,366 – 75,145). Neither the peak nor the AUC of the asexual parasitaemia were statistically significantly different between groups (p=0·478 and p=0·977 respectively, Mann-Whitney U; Figure 2).

### Clinical inflammation parameters

**Figure 3:**
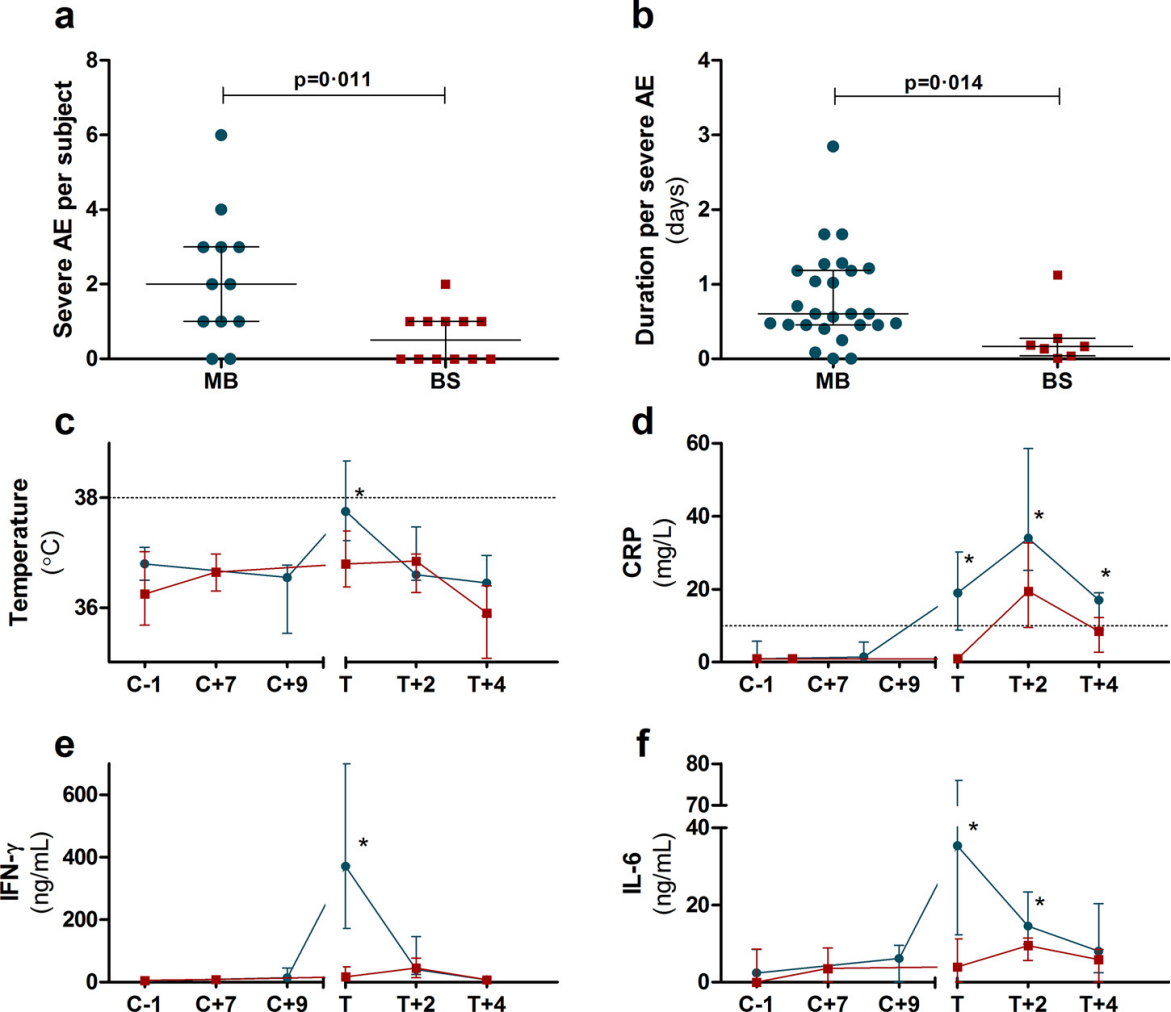
Adverse events and inflammaotry parameters per study group. a) Absolute number of severe adverse events per subject. b) Median duration in days of severe adverse events per subject in days. Bullets in a+b represent individual subjects infected by mosquito bite (MB, blue circles) or blood-stage inoculation (BS, red squares); bar and whiskers represent median and IQR; p-values for difference between groups by Mann-Whitney-U. c) Body temperature (tympanic). d) C-reactive protein concentrations. e) Serum IFN-γ concentrations. f) Serum IL-6 concentrations. Figures c-f show median values for MB (blue circles) and BS (red squares), with error bars for IQR. Dotted lines represent threshold for normal values. Time points are indicated as days relative to challenge infection (C+#) or treatment (T+#). Asterisks indicate a significant difference between inoculation groups at a given time point (p<0•05, Mann-Whitney U).

All volunteers experienced adverse events (AEs), but none were serious adverse events. The median number of AEs per subject (mild, moderate and severe) did not differ significantly between groups, with 13·5 AE/subject (range 9 – 25) in the MB-group and 11 AE/subject (range 5 – 35) in the BS-group (Supplemental table 2). However, the number of severe (grade 3) adverse events was significantly lower in the BS-group (median MB-group: 2·0 AE/subject, range 0 – 6; median BS-group: 0·5 AE/subject, range 0 – 2; p=0·011, Mann-Whitney U). In addition, the median duration per AE (mild, moderate or severe) was significantly shorter in the BS-group (median MB-group: 18 hours, range 1 minute – 7 days; median BS-group: 12 hours, range 1 minute – 8 days; p=0·007, Mann-Whitney U). Moreover, the median duration per severe AE was also significantly lower in the BS-group (median MB-group: 14.5 hours, range 1 minute – 68 hours; median BS-group 4 hours, range 15 minutes – 27 hours; p=0·014, Mann-Whitney U). Fever, defined as a temperature ≥38·0°C, occurred slightly more frequently in the subjects infected by MB (12/12) compared to BS (9/12), though this was not statistically significant (p=0·109, Fisher's exact). At time of treatment, temperature was significantly different between the two groups (median MB 37·8°C, range 36·4 – 39·0°C; median BS 36·8°C, range 35·8 – 37·8°C; p=0·002, Mann-Whitney U; Figure 3). Statistically significant differences at time of treatment were also found in C-reactive protein (CRP) (median MB-group 19 mg/L, range 5 – 54 mg/L; median BS-group 1 mg/L, range 1 – 22 mg/L; p<0·001, Mann-Whitney U), platelet count (median MB-group 168 *10^9^/L, range 83 – 259 *10^9^/L; median BS-group 234 *10^9^/L, range 182 – 271 *10^9^/L; p=0·014, Mann-Whitney U) and lymphocyte count (median MB-group 0·72 *10^9^/L, range 0·32 – 1·39*10^9^/L; median BS-group 1 ·46 *10^9^/L, range 0·74 – 2·28 *10^9^/L; p<0·001, Mann-Whitney U). In particular grade 3 liver enzyme abnormalities, considered a marker of systemic inflammation in uncomplicated malaria ^6^, were more common following MB-infection (Supplemental figure 1). The incidence and severity of liver enzyme abnormalities in the MB-infected group was comparable to a previous CHMI study conducted by MB.^6^^,^^7^

### Circulating Cytokines

Plasma IFN-γ and IL-6 concentrations were significantly higher immediately prior to treatment (T) in MB-infected subjects than in BS subjects (Figure 3). MCP1, IL-8 and IL-10 were similarly elevated in MB compared to BS at time of treatment (Supplemental figure 2). Moreover, while cytokine responses in MB-infected subjects generally peaked at time of treatment, responses in BS-infected subjects generally showed an increase at day 2 post-treatment (T+2) relative to baseline, while still remaining below those of MB-infected subjects. Furthermore, peak cytokine concentrations (IFN-γ, IL-6, MCP1, IL-8 and IL-10), were significantly higher in the MB-inoculated subjects (Supplemental figure 2). IL1-b, IFN-α, TNF-α, IL33, IL17a and IL23 showed no significant changes over time in either of the cohorts and IL12p70, IL18 remained below the limit of detection (data not shown).

### In vitro cytokine responses in (semi-)innate cells

**Figure 4:**
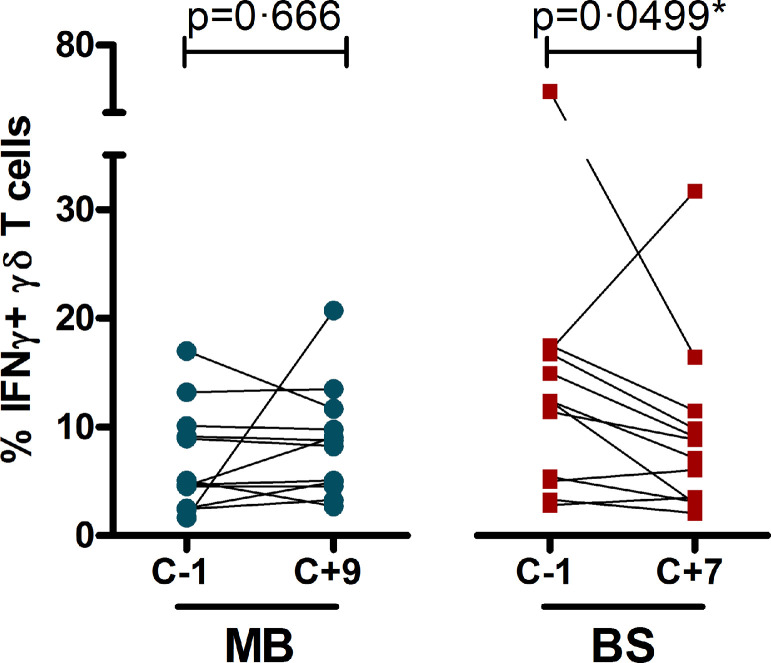
In vitro stimulation of γδ T-cells. The proportion of IFNγ+ γδ T-cells after *in vitro* stimulation with PfRBCs. Figures show values for individual mosquito bite infected- (MB, blue circles, n=12) or blood-stage infected subjects (BS, red squares, n=12) at baseline (one day before challenge C-1) and respectively day 9 or day 7 post-challenge (C+9, C+7); values corrected for proportions of IFNγ+ cells stimulated with uninfected RBCs (range 0·25 - 8·14 %). P-values by Wilcoxon signed-rank test.

To further dissect the nature of the inflammatory response, BS and MB subjects’ PBMC were stimulated *in vitro* with *Pf*-infected RBC at different study time points (C-1 and either C+7 or C+9, respectively). The proportion of *Pf*-specific IFN-γ+ cells were assessed by flow cytometry, focusing in particular on the innate(-like) γδ T-cell, NK-cell and monocyte populations in both study groups. While total numbers of γδ T-cells, NK-cells and monocytes remained stable over the two respective time points, there was a decrease in the percentage of responding IFN-γ+ cells in the BS-group at day 7 post-infection (C+7, i.e. after 7 days of exposure to (sub-microscopic) parasitaemia), most pronounced and reaching significance in the γδ T-cell population (Figure 4, Supplemental figure 3). In contrast, the proportion of *Pf*-responding IFN-γ+ cells in the MB-group did not differ at C+9 (i.e. after only 2 days of detectable parasitaemia) compared to baseline. We did not detect significant differences in monocyte or lymphocyte activation parameters including HLA-DR and CD86 or CD69, respectively (not shown). The combined data show an *in vitro Pf-*hypo-responsiveness over time in particularly γδ T-cells in the BS-subjects.

## Discussion

In this study, combined evidence shows a stronger inflammatory profile in MB- compared to BS- infection with the same *P. falciparum* 3D7 parasite line in malaria-naïve volunteers, despite similar asexual parasite densities. This comprises the more frequent occurrence and longer duration of grade 3 adverse events in the MB-group. Objective measures including temperature and clinical parameters of inflammation such as CRP, platelet and lymphocyte counts and liver enzymes also suggest more severe systemic inflammation in the MB-infected subjects. The difference in inflammatory status between the two groups is further confirmed by significant differences in a number of circulating cytokines (IFN-γ, IL-6, MCP1, IL-8 and IL-10). The mechanism behind these observations remains speculative, but the host response to parasite kinetics may play an important role. In the BS-group, with a gradual increase of parasite densities over 8 days starting at unphysiologically low parasite densities (a total of roughly 2,800 parasites, ∼10% of the parasites estimated to emerge from a single infected hepatocyte^8^), an inflammatory reaction may only become overt upon drug treatment by exposure of (semi)-innate cells to the resultant increased parasite debris. In contrast, in the MB-group inflammation is already initiated a few days before treatment, when abundant numbers of asexual parasite forms are released into the circulation from bursting liver schizonts^3^.

Although treatment initiation criteria differed slightly between the two groups, this still resulted in similar asexual AUC and peak parasite densities - generally considered to be prime drivers of inflammation. Despite being treated earlier, the overall duration of exposure to the symptomatic asexual stages was actually longer in the BS-group (Figure 1). Therefore we believe that the observed milder symptoms and signs of inflammation in the BS-group cannot be explained by the difference in treatment criteria or shorter duration of parasite exposure, but are indeed inherent to the inoculation method.

Our *in vitro* stimulation assays suggest that reduced (semi)-innate PBMC responsiveness in the BS-group may play a role, thereby modulating the severity of clinical symptoms. The decreased IFN-γ production after stimulation with *Pf*RBCs *in vitro* may be due to exposure to relatively low numbers of blood-stage parasites over a sustained time period. This observation is most pronounced in the γδ T-cell subset previously identified as one of the main IFNγ-producers in response to *Pf*RBC.^9^ These findings may suggest an induced attenuation of inflammatory responses by very low numbers of injected parasites. Furthermore, the BS-group has a much longer window of sub-PCR parasitaemia which may contribute to a degree of parasite tolerance. This finding may explain our *in vivo* observations of milder symptomatology, lower levels of inflammatory parameters and cytokines (including IFN-γ) in the BS-group. Yet, attenuation of induced inflammatory responses is not reflected by an increase of circulating IL-10 in the BS-group (Supplemental figure 2), suggesting that this cytokine does not represent the immunological mechanism that prevents inflammation. The kinetics of parasite exposure in BS may induce a tolerogenic or immunoregulatory effect through (an) alternate immunological pathway(s), that in turn limit(s) the development of inflammation. As we considered the elapsed time from first exposure to parasites to the sampled time points too short to expect significant adaptive responses, our current flow analysis focused on (semi-)innate immunity. It would however be interesting to include regulatory cellular immune responses in future investigations.

It should be noted that a combination of factors might be responsible for the observed difference in clinical inflammatory signals. In addition to human host factors, differences in parasite characteristics may play a role including parasite multiplication rate or virulence as a result of mosquito- or liver passage. For instance, changes do occur in variant surface antigen (VSA) expression profile after mosquito passage.^10^^,^^11^ As expression of certain VSA subsets are associated with severe malaria^12^, such a change may result in a difference in clinical presentation. Recently, however, no transcriptional evidence was found for the expansion of VSA variants associated with severe malaria in early stage malaria infections in either BS or MB infected malaria naïve subjects.^13^

Interestingly and contrary to our findings, in rodent *Plasmodium* species, increased disease severity is observed after (serial) blood passage of asexual blood-stages.^14^^,^^15^ This increased pathogenicity could be reset in a murine *P. chabaudi* model by mosquito passage of the parasites.^10^ In the early 20^th^ century, blood passage of *P. vivax* parasites through the human host was frequently used to treat patients suffering from neurosyphilis. Given the large numbers of patients receiving this therapy, it is remarkable that only few literature reports describe increased pathogenicity after blood passage through the human host.^16^^,^^17^ Moreover, during this period it was described that passing parasites through the mosquito vector increases the severity of human infections.^18^ These contrasting findings highlight that observations in murine models using unnatural parasite-host combinations may not always translate to human malaria infections.

In this trial transmissible gametocytaemia was only observed in BS-volunteers.^3^ In the light of our current findings, this appears to be in contrast with the generally accepted view that gametocyte emergence is enhanced by cell stress and inflammation. Previously it has been shown that a decrease in host derived lysophosphatidylcholine (LysoPC) stimulates parasite gametocytogenesis, where LysoPC decreases are associated with severe (fulminant) infections.^19^ It remains speculative whether there is a causative relationship between severity of infection and sexual commitment, or whether other factors such as duration of parasitaemia or parasite multiplication rate are responsible for higher sexual commitment.

A number of possible limitations can be considered. The post-hoc analyses were not part of the original study objectives, sample sizes were small and the trial was conducted in a single centre. Although the observed inflammatory and symptomatic differences between the study groups were confirmed by multiple parameters, for unequivocal evidence of induced tolerogenic responses in γδ T-cells, this should be confirmed by a second dataset.

In conclusion, *Pf*-infections by MB induce stronger signs and symptoms of inflammation compared to CHMI by BS-infection at similar parasite densities. The pathophysiological basis remains speculative but may relate to immune tolerance induced by prolonged low density parasitaemia following BS-infection. These results are of importance for our understanding of parasite-host interaction. Finally, these results highlight that caution is needed when interpreting and comparing safety and efficacy results obtained from CHMI trials with different infection routes.

## Contributors

Isaie J Reuling, Christian F Ockenhouse, Katharine A Collins, Teun Bousema and Robert W Sauerwein designed the study. Manon Alkema, Gerdie M de Jong, Isaie J Reuling, Quirijn de Mast, Reinout van Crevel, Teun Bousema and Robert W Sauerwein conducted the clinical trial.

X Zen Yap, Matthew BB McCall, Robert W Sauerwein and Manon Alkema designed, performed and analysed the laboratory experiments. X Zen Yap, Matthew BB McCall, Robert W Sauerwein and Manon Alkema wrote the first draft of the manuscript, had access to and verified the data. All authors contributed to the final manuscript, had full access to the data and accept responsibility to submit for publication.

## Declaration of interests

RS received consulting fees from Biomedical Primate Research Centre, Rijswijk, The Netherlands and has stocks in TropIQ Health Sciences, TropIQ, the Netherlands. MA was supported by PATH's Malaria Vaccine Initiative. ZY was supported by the AMMODO science award 2019 awarded to TB. All other authors: No reported conflicts.

## Appendix Supplementary materials

Image, application 1

Image, application 2

Image, application 2
