# Supplementary material for: Controlled human malaria infections by mosquito bites induce more severe clinical symptoms than asexual blood-stage challenge infections
Source: eBioMedicine. 2022 Mar 9;77:103919. doi: 10.1016/j.ebiom.2022.103919 (PMC8917304; doi:10.1016/j.ebiom.2022.103919)
Supplement: Supplementary file 1 [file mmc1.pdf]

Supplementary Material

Table of contents

Supplemental figure 1 – Liver enzyme abnormalities 2

Supplemental figure 2 – Circulating cytokine serum levels 3

Supplemental figure 3 – *In vitro* stimulation of NK cells and CD14+-monocytes 4

Supplemental Table 1 – Normal values for clinical laboratory parameters. 5

Supplemental table 2 – List of adverse events possibly or probably related to the trial. 6

References 7

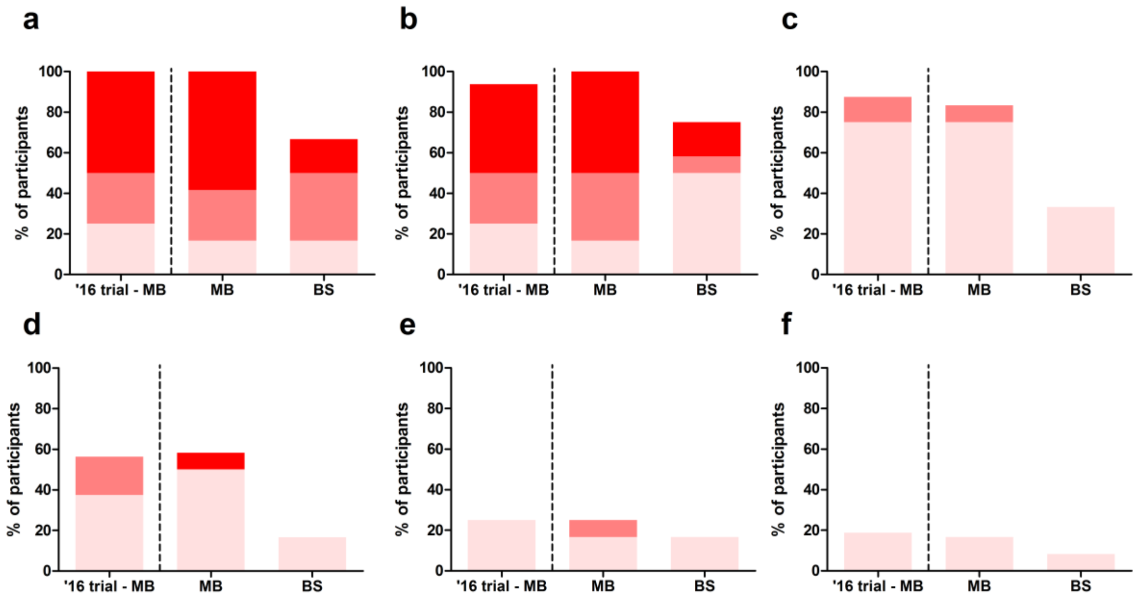

Supplemental figure 1 – **Liver enzyme abnormalities.** Peak laboratory abnormalities of **a)** AST, **b)** ALT, **c)** LDH, **d)** gamma GT, **e)** Alkaline phosphatase and **f)** bilirubin were scored mild (1·1-2·5*ULN, light pink), moderate (2·5-5*ULN, dark pink) or severe (>5*ULN, red) for each individual and shown as percentage of participants in the mosquito bite subjects (MB, n=12) and blood stage infected subjects (BS, n=12), compared to MB-infected subjects in a previous CHMI study from 2016 [1,2]('16 trial – MB, n=16).

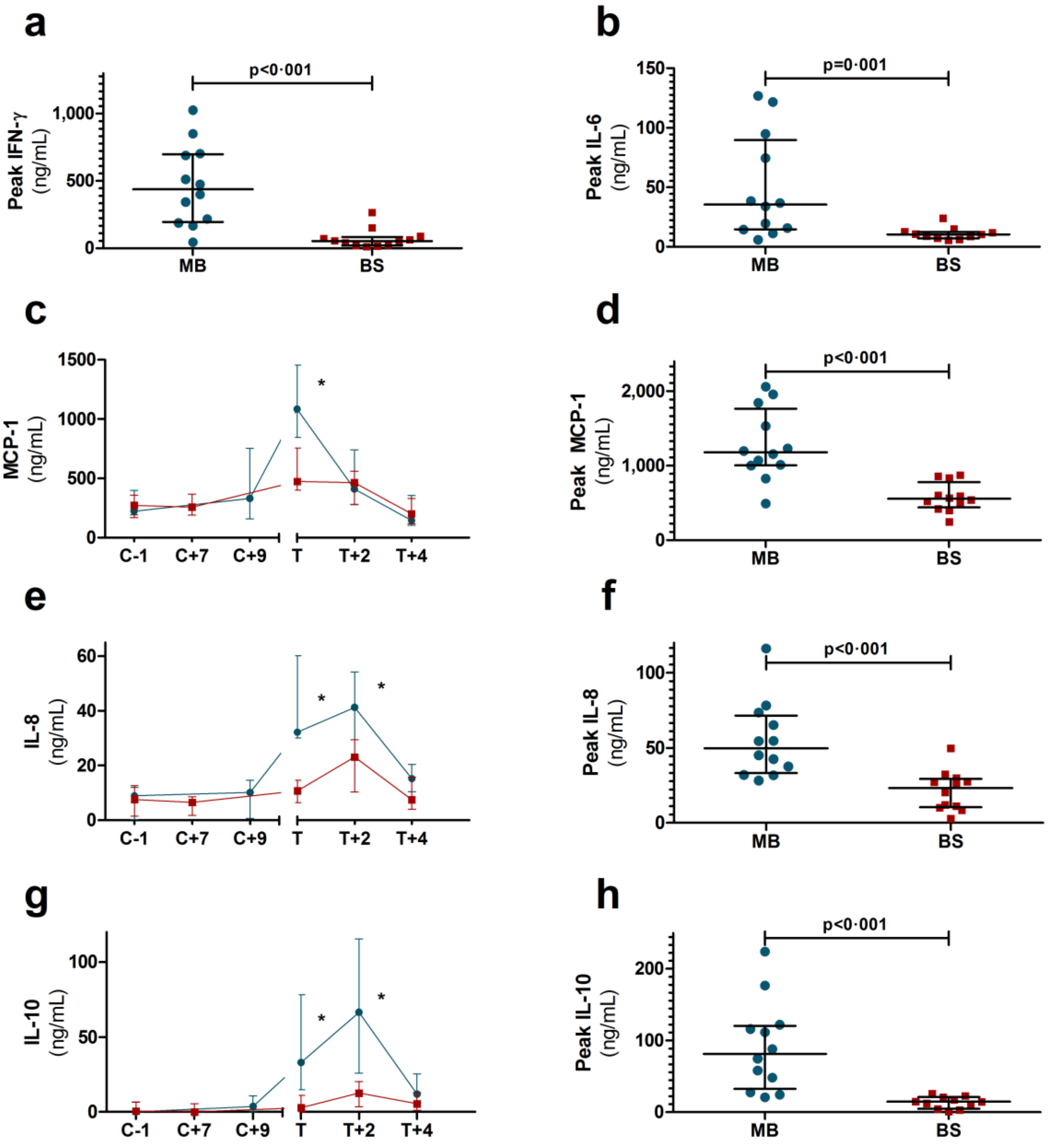

Supplemental figure 2 – Circulating cytokine serum levels.

a) Peak serum concentrations of IFN-γ. **b)** Peak serum IL-6 concentrations. **c)** Serum concentrations of MCP-1. **d)** Peak serum concentrations of MCP-1. **e)** Serum IL-8 concentrations. **f)** Peak serum IL-8 concentrations. **g)** Serum IL-10 concentrations. **h)** Peak serum IL-10 concentrations. Data in a, b, d, f and h represent individual values and data in c, e and g represent median values for MB (blue circles) and BS (red squares), with error bars for IQR. Time points are indicated as days relative to challenge infection (C+#) or treatment (T+#). Asterisks indicate a significant difference between inoculation groups at a given time point ($p<0{\cdot}05$, Mann-Whitney-U).

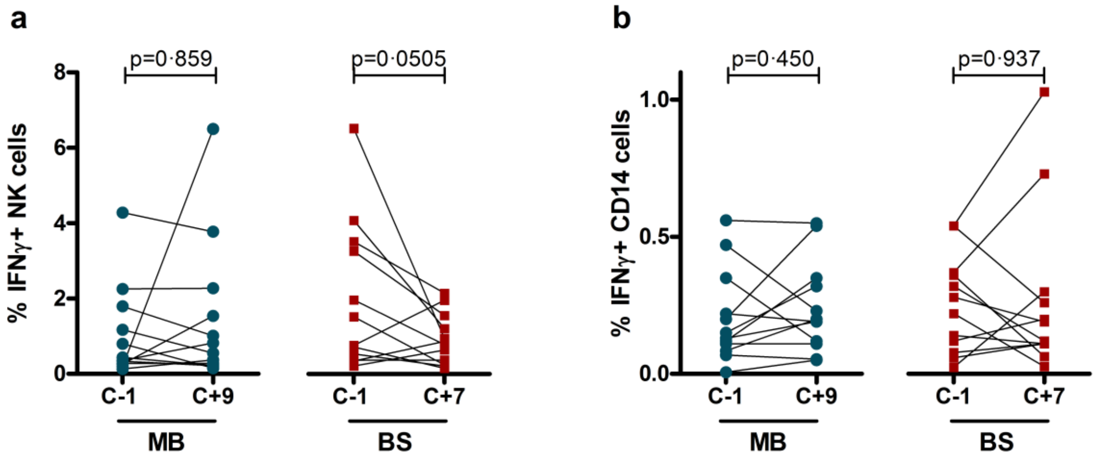

Supplemental figure 3 – *In vitro* stimulation of NK cells and CD14+-monocytes. The proportion of IFNγ+ cells after *in vitro* stimulation with *Pf*RBCs in the **a)** NK cell population and **b)** monocyte (CD14+) population. Figures show values for individual mosquito bite infected subjects (MB, blue circles, n=12) and blood stage infected subjects (BS, red squares, n=12) at baseline(C-1 and (C+9 and C+7 respectively); values corrected for proportions of IFNγ+ cells stimulated with uninfected RBCs (range 0·00 - 1·59 % of NK cells, 0·00 - 0·22 % of CD14+ monocytes). P-values by Wilcoxon signed-rank test.

LABORATORY PARAMETER	NORMAL VALUE
LYMPHOCYTE COUNT	1·0*10^{9}/L – 3·5*10^{9}/L
THROMBOCYTE COUNT	150*10^{9}/L – 400*10^{9}/L
CRP	<10 mg/L
ALT	<35 U/L (females) / <45 U/L (males)
AST	<30 U/L (females) / <35 U/L (males)
LDH	<250 U/L
GAMMA GT	<40 U/L (females) / <55 U/L (males)
ALP	<100 U/L (females) / <115 U/L (males)
BILIRUBIN	<17 µmol/L

Supplemental Table 1 – Normal values for clinical laboratory parameters.

	All adverse events (grade 1, 2 and 3)					
	MB			BS		
	No of subjects	No of episodes	Median duration in days (Range)	No of subjects	No of episodes	Median duration in days (Range)
**Headache**	12	53	0·8 (0·0-4·0)	12	53	0·7 (0·0-4·0)
**Fever**	12	27	0·5 (0·0-1·3)	9	18	0·3 (0·0-1·1)
**Chills**	7	15	0·4 (0·0-3·4)	5	9	0·2 (0·0-4·6)
**Nausea**	9	25	0·6 (0·0-2·3)	8	14	0·3 (0·0-2·0)
**Fatigue**	9	20	1·0 (0·1-3·9)	8	18	0·8 (0·0-8·0)
**Malaise**	8	23	1·5 (0·0-3·9)	3	5	0·9 (0·1-6·5)
**Myalgia**	6	7	2·0 (0·9-3·2)	8	12	0·6 (0·1-5·2)
**Decreased appetite**	5	5	3·5 (0·6-6·9)	2	3	1·3 (0·9-3·0)
**Dizziness**	3	8	1·1 (0·0-1·5)	4	12	0·9 (0·0-4·0)
**Abdominal pain**	2	2	0·5 (0·0-0·9)	7	8	0·4 (0·0-2·0)
**Diarrhea**	2	3	0·3 (0·0-1·7)	1	1	2
**Back pain**				2	3	0·9 (0·1-2·0)
**Syncope**	1	1	0			
**Palpitations**	1	1	5·3			
**Arthralgia**				1	1	0·3
**Nonspecific thoracic pain**				1	2	0·1 (0·0-0·2)
**Total**	**12**	**190**		**12**	**159**	
	**Grade 3 adverse events**					
**Headache**	2	3	0·6 (0·5-1·0)			
**Fever**	4	5	0·5 (0·1 -1·3)	4	5	0·2 (0·0-1·1)
**Chills**	2	2	0·8 (0·3-1·3)			
**Nausea**	4	4	0·5 (0·0-1·7)	1	1	0
**Fatigue**	4	4	0·9 (0·6-1·7)			
**Malaise**	4	5	0·7 (0·5-2·8)			
**Dizziness**	2	2	0·8 (0·5-1·2)			
**Abdominal pain**				1	1	0·2
**Syncope**	1	1	0			
**Total**	**10**	**26**		**6**	**7**	

Supplemental table 2 – List of adverse events possibly or probably related to the trial.

References

1. Reuling IJ, van de Schans LA, Coffeng LE, et al. A randomized feasibility trial comparing four antimalarial drug regimens to induce Plasmodium falciparum gametocytemia in the controlled human malaria infection model. *eLife* 2018; **7**.

2. Reuling IJ, de Jong GM, Yap XZ, et al. Liver Injury in Uncomplicated Malaria is an Overlooked Phenomenon: An Observational Study. *EBioMedicine* 2018; **36**: 131-9.
