# Supplementary material for: Controlled human malaria infections by mosquito bites induce more severe clinical symptoms than asexual blood-stage challenge infections
Source: eBioMedicine. 2022 Mar 9;77:103919. doi: 10.1016/j.ebiom.2022.103919 (PMC8917304; doi:10.1016/j.ebiom.2022.103919)
Supplement: Supplementary file 2 [file mmc2.pdf]

CONFIDENTIAL

CLINICAL TRIAL PROTOCOL

(23 January 2018)

Controlled Human Malaria Infection model for evaluation of transmission-blocking interventions – Study 2

Version 3.0, 23-January-2018

(“*CHMI-trans2*”)

PROTOCOL TITLE: ‘Controlled Human Malaria Infection study to assess gametocytemia and mosquito transmissibility in participants challenged with *Plasmodium falciparum* by sporozoite or blood stage challenge to establish a model for the evaluation of transmission-blocking interventions’

**Protocol ID**	**CHMI-trans2**
**Short title**	Controlled Human Malaria Infection model for evaluation of transmission-blocking interventions – Study 2
**Toetsingonlinenummer**	NL63552.000.17
**EudraCT number**	*2017-004005-40*
**Version**	**2.0**
**Date**	**25-11-2017**
**Project leader/Clinical investigator**	**Isaie J. Reuling, M.D.**<br>Radboudumc, Department of Medical Microbiology<br>Tel: +31 (0)24 361 9515<br>Fax: +31 (0)24 3614666<br>E-mail: isaie.reuling@radboudumc.nl
**Sponsor**<br><br><br><br><br>**Principal Investigator**<br><br><br><br><br><br>**Sub- investigator**	Stichting Katholieke Universiteit<br>Radboud University Medical Centre (Radboudumc)<br>Department of Medical Microbiology<br>Geert Grooteplein-Zuid 10<br>6525 GA, Nijmegen, The Netherlands<br><br>**Prof. Robert W. Sauerwein, M.D.**<br>Radboudumc, Department of Medical Microbiology<br>Tel: +31243610577<br>Fax: +31243614666<br>e-mail: robert.sauerwein@radboudumc.nl<br><br>**Dr. Teun Bousema**<br>Radboudumc, Department of Medical Microbiology<br>Tel: +31243617574<br>Fax: +31243540216<br>e-mail: teun.bousema@radboudumc.nl
**Funding partner**	**PATH**<br>2201 Westlake Avenue, Suite 200<br>Seattle, Washington<br>98121, USA

**Clinical supervisors**	**Prof. André van der Ven, M.D.**<br>Radboudumc, Department of Internal Medicine<br>Tel: +31 24 368 6791<br>e-mail: andre.vanderven@radboudumc.nl<br><br>**Dr. Quirijn de Mast, M.D.**<br>Radboudumc, Department of Internal Medicine<br>Tel: +31 24 361 0929<br>e-mail: quirijn.demast@radboudumc.nl
**Biological evaluator**	**Dr. Kjerstin Lanke**<br>Radboudumc, Department of Medical Microbiology<br>Tel: +31 (0)24 361 9186<br>E-mail: kjerstin.lanke@radboudumc.nl<br><br>**Matthijs Jore, PhD**<br>Radboudumc, Department of Medical Microbiology<br>Tel: +31 (0)24 361 0583<br>E-mail: Matthijs.Jore@radboudumc.nl
**Independent expert (s)**	Radboudumc<br>Department of Internal Medicine<br>**Dr. Monique Keuter**<br>Tel : +31 (0)24 361 4462<br>Monique.Keuter@radboudumc.nl
**Laboratory sites**	**Radboudumc Clinical Chemical Laboratory**<br>**Jan Verhagen**<br>Tel: +31 (0)24 3616997<br>Fax: +31 (0)24 3568408<br>e-mail: j.verhagen@akc.umcn.nl<br><br>**Clinical Microbiology Laboratory**<br>**Prof.dr. Heiman Wertheim**<br>Tel: +31 (0)24 36 14281<br>e-mail: Heiman.Wertheim@radboudumc.nl
**Pharmacy**	**Radboudumc Clinical Pharmacy**<br>Clinical Trial Unit<br>**Dr. Rob ter Heine**<br>Tel: +31 (0)24 3616405<br>Fax: +31 (0)24 3668755<br>e-mail: R.terHeine@radboudumc.nl

**External monitor**	**CROMSOURCE**<br>Lange Dreef 11H<br>4131 NJ Vianen, The Netherlands<br>Tel: +31 347 344977 ext. 2614<br>Fax: +31 347 34 42 89<br>Mobile: +31 6 13516468

PROTOCOL SIGNATURE SHEET

Investigator Agreement

"I have read this protocol and agree to abide by all provisions set forth therein.
I agree to comply with the principles of the International Conference on Harmonisation Tripartite Guideline on Good Clinical Practice."

Name	Signature	Date
Principal investigator:<br>***Robert Sauerwein, MD, PhD***<br>*Department of Medical Microbiology*<br>*Radboud university medical center*		
Sub-investigator:<br>***Teun Bousema, PhD***<br>*Department of Medical Microbiology*<br>*Radboud university medical center*		
Project leader:<br>***Isaie Reuling, MD***<br>*Department of Medical Microbiology*<br>*Radboud university medical center*		

Version History

Version	Date	Author(s)	Summary of changes
2.0	25 November 2017	**Isaie Reuling**<br>**Robert Sauerwein**<br>**Teun Bousema**	- Exploratory objective (skin odor) is removed from protocol.<br>- Section 9.3.2 has been adapted (ALT/AST treatment criteria).<br>- Extra information added on treatment threshold added to section 1.2 and 7.4.<br>- Additional information on exploratory objective is added in section 9.3.14.<br>- Extra information on adverse events in previous studies have been added to section 1.2, 7.4, and 8.4.<br>- Adjusted the piperaqune tablet strength to 160mg.<br>- Additional information on biological samples of study in section 13.1.1.
3.0	23 January 2018	**Isaie Reuling**<br>**Robert Sauerwein**<br>**Teun Bousema**	- Eploratory objective on elevated transaminases has been added to section 2 and 9.1.3<br>- Rh(c) and/or Rh(e) negative women will

			be excluded to participate in cohort B. History of blood transfusion also excludes participation in Cohort B (Section 4.3, 8.4 and 14.5.)

TABLE OF CONTENTS

Inhoud

1. INTRODUCTION AND RATIONALE 16
1.1. Introduction 16
1.2. Rationale 16
1.3. Clinical Experience 18
1.4. Safety 18
2. OBJECTIVES 21
3. STUDY DESIGN 22
4. STUDY POPULATION 24
4.1. Population (base) 24
4.2. Inclusion criteria 24
4.3. Exclusion criteria 25
5. SAMPLE SIZE CALCULATION 27
TREATMENT OF SUBJECTS 28
5.1. Investigational product/treatments (for T1,T2, and T3) 28
5.2. Investigational product Use of co-intervention 28
5.3. Escape medication (if applicable) 28
6. INVESTIGATIONAL PRODUCT (TREATMENT PRODUCT) 29
6.1. Name and description of investigational products 29
6.2. Summary of findings from non-clinical studies 29
6.3. Summary of findings from clinical studies 29
6.3.1. Clinical data for sulfadoxine-pyrimethamine 29
6.3.2. Clinical data for Piperaquine 29
6.3.3. Pharmacokinetics and Metabolism of sulfadoxine-pyrimethamine 30
6.3.4. Pharmacokinetics and Metabolism of Piperaquine 30
6.3.5. Safety and tolerability of sulfadoxine-pyrimethamine 31
6.3.6. Safety and tolerability of Piperaquine 31
6.4. Drug and dose selection rationale 31
6.5. Potential Risks 32
6.6. Potential Benefits 33
6.7. Risk Management 33
6.8. Preparation and labeling of Investigational Medicinal Product 33
6.9. Drug accountability 34
7. NON-INVESTIGATIONAL PRODUCT (SPOROZOITE CHALLENGE PRODUCT) 35
7.1. Name and description of non-investigational product(s) 35
7.2. Summary of findings from non-clinical studies 35
7.3. Summary of findings from clinical studies 35
7.4. Summary of known and potential risks and benefits 35
7.5. Description and justification of route of administration and dosage 36
7.6. Dosages, dosage modifications and method of administration 36
7.7. Preparation and labeling of Non Investigational Medicinal Product 37
7.8. Drug accountability 37
8. NON-INVESTIGATIONAL PRODUCT (BLOOD STAGE CHALLENGE PRODUCT) 38
8.1. Name and description of non-investigational product(s) 38
8.2. Summary of findings from non-clinical studies 38
8.3. Summary of findings from clinical studies 38
8.4. Summary of known and potential risks and benefits 38
8.5. Description of route of administration and dosage 39
8.6. Preparation and labeling of Non Investigational Medicinal Product 39

8.7. Drug accountability 40
9. METHODS 41
9.1. Study parameters/endpoints 41
9.1.1. Main study parameter/endpoint 41
9.1.2. Secondary study parameters/endpoints 41
9.1.3. Exploratory study parameters/endpoints 41
9.2. Randomisation, blinding and treatment allocation 41
9.3. Study procedures 42
9.3.1. Screening period: Screening, Inclusion and Baseline visits 42
9.3.2. Controlled Human Malaria Infection 42
9.3.3. Treatment with piperaquine (T1, T2, and T3 for group 1 and 3) 44
9.3.4. Treatment with sulfadoxine-pyrimethamine (T3 for groups 2 and 4) 45
9.3.5. Treatment with Malarone® (ET) 45
9.3.6. Physical examination 45
9.3.7. Vital signs 45
9.3.8. Patient-reported outcomes (study diary) 46
9.3.9. Electrocardiogram 46
9.3.10. Blood sampling and safety laboratory evaluations 46
9.3.11. Analysis of asexual parasite densities after challenge infection 47
9.3.12. Direct Membrane Feeding Assays (DMFA) and Direct Skin Feeding Assays (DFA) 47
9.3.13. Quantification of gametocytes, and gametocyte sex ratio. 48
9.3.14. Immunological assays 49
9.3.15. Case report forms and data collection 49
9.3.16. Flowchart Study Design 50
9.4. Withdrawal of individual subjects 54
9.5. Replacement of individual subjects after withdrawal 54
9.6. Follow-up of subjects withdrawn from treatment 55
9.7. Premature termination of the study 55
10. SAFETY REPORTING 56
10.1. Temporary halt for reasons of subject safety 56
10.2. AEs, SAEs and SUSARs 56
10.2.1. Adverse events (AEs) 56
10.2.2. Serious adverse events (SAEs) 56
10.2.3. Suspected unexpected serious adverse reactions (SUSARs) 57
10.3. Follow-up of (serious) adverse events 57
10.3.1. Adverse event data collection 57
10.3.2. Assessment of causality 58
10.3.3. Follow-up of adverse events 58
10.4. Local Safety Monitor (LSM) and Safety Monitoring Committee (SMC) 58
10.4.1. Local safety monitor 58
10.4.2. Safety Monitoring Committee (SMC) 59
10.4.3. Review of safety data by the safety monitor and SMC 59
10.4.4. Safety stopping rules 59
11. STATISTICAL ANALYSIS 61
11.1. Primary study parameter(s) 61
11.2. Secondary study parameter(s) 61
11.3. Other study parameters 62
12. ETHICAL CONSIDERATIONS 63
12.1. Regulation statement 63
12.2. Recruitment and consent 63
12.3. Benefits and risks assessment, group relatedness 63
12.4. Ethical aspects concerning the production of P. falciparum infected mosquitoes 64

12.5. Ethical aspects concerning the use of human volunteers 64
12.6. Compensation for injury 64
12.7. Incentives 65
13. ADMINISTRATIVE ASPECTS, MONITORING AND PUBLICATION 66
13.1. Handling and storage of data and documents 66
13.1.1. Confidentiality 66
13.1.2. Data collection 66
13.1.3. Database management and quality control 66
13.2. Monitoring and Quality Assurance 66
13.3. Amendments 67
13.4. Annual progress report 67
13.5. Temporary halt and (prematurely) end of study report 67
13.6. Public disclosure and publication policy 68
14. STRUCTURED RISK ANALYSIS 69
Controlled Human Malaria Infection 69
14.1. Potential issues of concern 69
14.2. Cardiac events following Controlled Human Malaria Infections 71
14.3. Transient liver function test derangements 73
14.4. Transient serum creatinine elevations 74
14.5. Risk management of reaction to the blood stage inoculum sample 74
14.6. Synthesis 75
15. REFERENCES 76
16. APPENDICES 82

LIST OF ABBREVIATIONS AND RELEVANT DEFINITIONS

A.	*Anopheles*
ACT	Artemisinin-Based Combination Treatment
AE	Adverse Event
AL	artemether–lumefantrine
ALT	Alanine Aminotransferase
ANOVA	analysis of variance
AR	Adverse Reaction
BMI	Body mass index
BP	Blood pressure
BSL	Biosafety Level
CA	Competent Authority
CCMO	Central Committee on Research Involving Human Subjects; in Dutch: Centrale Commissie Mensgebonden Onderzoek
CHMI	Controlled Human Malaria Infection
CHMI-trans	Controlled Human Malaria Infection Transmission Model
CRF	Case Report Form
CRO	Contract Research Organization
CV	Curriculum Vitae
DFA	Direct Skin Feeding Assay
DHA	dihydroartemisinine
DHA-PQP	Dihydroartemisinine - piperaquine phosphate
DMFA	Direct Membrane Feeding Assay
T1	Treatment 1
T2	Treatment 2
T3	Treatment 3
ECG	ElektroCardioGram
EDTA	Ethylenediaminetetraacetic acid
ET	End Treatment with Malarone®
eCRF	electronic Case Report Form
ELISA	Enzyme-Linked Immuno Sorbent Assay
G6PD	Glucose-6-phophatedehydrogenase deficiency
GCP	Good Clinical Practice
GP	General Practitioner
HBV	Hepatitis B Virus
HCV	Hepatitis C Virus
HDPE	high density polyethylene
HIV	Human Immunodeficiency Virus
HTLV	HumanT-lymphotropic Virus
IC	Informed Consent
IFN-γ	Interferon-gamma
IL-1β	Interleukin 1β
IL-6	Interleukin 6
IPTp	intermittent preventive treatment of malaria in pregnancy

**IRB**	**Institutional Review Board**
**ITN**	**insecticide-impregnated bednets**
**IV**	**Intravenous**
**LDH**	**Lactate dehydrogenase**
**LD-PIP**	**Low dose of piperaquine (480mg)**
**LSM**	**Local Safety Monitor**
**mAbs**	**Monoclonal antibodies**
**METC**	**Medical research ethics committee (MREC); in Dutch: medisch ethische toetsing commissie (METC)**
**MFS**	**Membrane Feed for Sporozoite production**
**NaCl**	**Sodium Chloride**
**NF54**	**Nijmegen *falciparum* strain 54**
**NK cells**	**Natural killer cells**
P.	***Plasmodium***
**par/ml or p/ml**	**parasites per milliliter**
**PATH REC**	**PATH Research Ethics Committee; the funding partner's ethical committee.**
**PBMC**	**Peripheral blood mononuclear cell**
**PCR**	**Polymerase Chain Reaction**
**Pf**	***Plasmodium falciparum***
**PhHV**	**Phocine Herpes Virus**
**PIP**	**Curative dose of piperaquine (960mg)**
**qPCR**	**Real-time Quantitative Polymerase Chain Reaction**
**qRT-PCR**	**Quantitative Reverse Transcriptase Polymerase Chain Reaction**
**QT-NASBA**	**Quantitative Nucleic Acid Sequence Based Amplification**
**Radboudumc**	**Radboud university medical center**
**SAE**	**Serious Adverse Event**
**Sanquin**	**Sanquin Blood Supply Foundation, who on the basis of the Blood Supply Act is responsible for all blood supply (blood and blood products) for transfusion in The Netherlands.**
**SCORE**	**Systematic Coronary Risk Evaluation**
**SMC**	**Safety Monitoring Committee**
**SMFA**	**Standard Membrane Feeding Assay**
**SOP**	**Standard Operating Procedure**
**SP**	**sulfadoxine-pyrimethamine**
**SPC**	**Summary of Product Characteristics**
**Sponsor**	**The sponsor is the party that commissions the organisation or performance of the research, for example a pharmaceutical company, academic hospital, scientific organisation or investigator. A party that provides funding for a study but does not commission it is not regarded as the sponsor, and is referred to here as a funding partner.**
**SWAB**	**Stichting Werkgroep Antibioticabeleid**
**SUSAR**	**Suspected Unexpected Serious Adverse Reaction**
**TBV(s)**	**Transmission Blocking Vaccine(s)**
**TNF-α**	**Tumor Necrosis Factor alpha**

**WIRB**	**Western Institutional Review Board; PATH's designated IRB to who PATH REC delegates ethical review and oversight of clinical studies.**
**WHO**	**World Health Organization**
**WMO**	**Medical Research Involving Human Subjects Act (in Dutch: Wet Medisch-wetenschappelijk Onderzoek met Mensen**
**ZAVIN**	**Ziekenhuis Afval Verwerkings Installatie Nederland (Hospital Waste Disposal company)**
**γGT**	**Gamma Glutamyl Transferase**

SUMMARY

Rationale:

Malaria is one of the most devastating infectious diseases worldwide. Despite all the progress that has been made in reducing the malaria burden, in 2015 there were still 212 million cases and 429,000 deaths, mainly in children less than five years of age(1). In addition to the intolerable clinical burden, malaria forms a profound economic burden for the affected countries, which are already struggling with poverty. The urgency of the situation is further emphasized by the waning effectiveness of all currently registered anti-malarials due to fast emergence and spread of resistance and the absence of an highly effective vaccine(2).

Malaria transmission blocking vaccines (TBVs) and transmission-blocking drugs aim to interrupt the development of parasites in the mosquito(3). TBVs will play a central role in efforts to reduce the malaria burden, to contain drug resistance and to move towards malaria elimination(2, 4).

The clinical development of such transmission blocking interventions will be greatly accelerated by a suitable model for their evaluation.

Controlled Human Malaria Infections (CHMI) are an established model for evaluation of malaria candidate vaccines and drugs targeting pre-erythrocytic or asexual blood stages.

The primary aim of this project is to develop a controlled human malaria infection transmission model ("*CHMI-trans*") or "challenge model" to evaluate the capacity of vaccines, biologics (monoclonal antibodies, or mAbs), and drugs to block malaria parasite transmission by assessing infectiousness of *Plasmodium falciparum* (*Pf*) gametocyte carriers for *Anopheles* mosquitoes.

Objective:

Primary objectives:

1) To evaluate the safety of CHMI-trans protocols in healthy malaria-naïve volunteers challenged with *Plasmodium falciparum* by sporozoite challenge and blood stage challenge.
2) To assess gametocyte infectiousness for *Anopheles* mosquitoes through mosquito membrane feeding assay (Direct Membrane Feeding Assay, DMFA).

Secondary objectives:

3) To determine the dynamics of gametocyte commitment, maturation and sex ratio by molecular markers of sexual stage development.
4) To determine the time-point of peak density of gametocytemia in the CHMI-trans model.
5) To assess gametocyte infectiousness for *Anopheles* mosquitoes through mosquito feeding assay (Direct Skin Feeding Assay, DFA).

Exploratory Objectives (see section 9.1.3 for detailed endpoints):

6) To determine gametocyte fitness by molecular markers, and *ex vivo* assessments of gamete formation and fertilization.
7) To analyse immune responses in volunteers exposed to a *Plasmodium falciparum* challenge infection

Study design:
Single center, open label, randomized, sporozoite and blood stage challenge study.

Study population:
A maximum of 24 healthy volunteers, aged 18 to 35 years, male and female, will participate in the study.

Intervention:
A total of 24 volunteers, in two cohorts (n=12), will be randomly assigned to two groups per cohort (n=6). **Cohort A** will be subjected to a standard controlled human malaria infection (CHMI) delivered by five *Pf*-infected mosquitoes (groups 1 and 2). **Cohort B** will be subjected to a standard blood stage challenge with ~2,800 *Pf*-infected erythrocytes by intravenous injection (groups 3 and 4) (see Figure 1, section 3).
Treatment is subsequently initiated to induce gametocytemia (treatment 1, T1) and to clear pathogenic asexual parasites whilst leaving gametocytes unaffected (treatment 2 and 3, T2 and T3). At the end of the study, treatment of all parasite stages is provided following national treatment guidelines (end treatment, ET).
Once malaria infections are detected by 18S qPCR positive at a density of 5,000 par/ml (sporozoite challenge) or on day 8 (blood stage challenge), all volunteers will be treated with a single oral subcurative low-dose of piperaquine (LD-PIP, 480 mg, T1). Daily blood samples will allow detailed quantification of gametocytes and gametocyte sex ratio. Using blood samples taken twice daily, the initial clearance of parasitemia will be carefully monitored. After T1, volunteers will receive a second treatment (T2, LD-PIP2, 480mg) if a recrudescence of asexual parasitemia (exceeding 1,500 par/ml by 18S qPCR) occurs before day 21 post challenge infection. On day 21 or when a recrudescence occurs after T2, volunteers in **group 1 and 3 (LD-PIP/LD-PIP2/PIP)** will be curatively treated with piperaquine (960mg) and **group 2 and 4 (LD-PIP/LD-PIP2/SP)** with sulfadoxine-pyrimethamine (1000mg/50mg). These treatment regimens cure asexual parasitemia while leaving immature and mature gametocytes unaffected(5). To ensure the radical clearance of all parasite stages, all volunteers will receive a final treatment (ET) according to national guidelines with atovaquone/proguanil (Malarone®) on day 36. In case a volunteer remains 18S qPCR and Pfs25 qRT-PCR negative for 7 days after T1 or T2, final treatment with Malarone® will also be initiated and end of study will apply for the volunteer. On the day of administration of T3 and three days after initiation of T3, venous blood samples will be obtained for Direct Membrane Feeding Assay (DMFA). An additional blood sample will be taken for DMFA between day 7-21 after T3, the exact time-point being based on the density of gametocytemia as measured by Pfs25 qRT-PCR. In addition, on the DMFA days volunteers will be subjected to Direct Skin Feeding Assays (DFA) with one cup of 20-30 mosquitoes, allowing mosquitoes to feed on the volunteer's forearms. These assays will provide evidence on the infectivity of volunteers at these time-points.

Main study parameters/endpoints:
Primary endpoints:
- Frequency and magnitude of adverse events in the CHMI-trans model.
- Prevalence of gametocytes and gametocyte infectiousness for *Anopheles* mosquitoes through mosquito feeding assay (Direct Membrane Feeding Assay, DMFA).

Secondary endpoints:

- Peak density and time-point of peak density of gametocytes by qRT-PCR.
- The area under the curve of gametocyte density versus time.
- Assessment of the dynamics of gametocyte commitment, maturation and sex-ratio.
- Prevalence of gametocyte infectiousness for *Anopheles* mosquitoes through Direct Feeding Assays (Direct Skin Feeding Assay, DFA).

Exploratory endpoints (see section 9.1.3 for detailed endpoints)

- Gamete formation and fertilization as ex vivo indicators of gametocyte fitness.

Nature and extent of the burden and risks associated with participation, benefit and group relatedness:

Benefits: There are no direct benefits for volunteers.

Risks: Risks for volunteers are related to (i) exposure to *P. falciparum* malaria infection by mosquito bites, (ii) exposure to *P. falciparum infected erythrocytes iii)* potential side-effects associated with treatment medications.

Burden:

The study represents a challenge infection by bites of 5 (3D7 *P. falciparum*) infected mosquitoes or a blood stage malaria infection through inoculation of infected RBCs (3D7 P. falciparum). After the challenge there will be a period (36 days) of intense clinical monitoring with frequent site visits (up to two times a day) and blood examinations. The subjects will receive a sub-curative treatment (T1) with piperaquine when 18S qPCR positive at 5000 par/ml (threshold of microscopic detection) or on day 8 for the blood stage challenge. Using blood samples taken twice daily, the initial clearance of parasitemia will be carefully monitored. After T1, volunteers will receive a second treatment (T2, LD-PIP2, 480mg) if a recrudescence of asexual parasitemia (exceeding 1,500 par/ml by 18S qPCR) occurs before day 21 post challenge infection. On day 21 or when a recrudescence occurs after T2, volunteers in **group 1 and 3 (LD-PIP/LD-PIP2/PIP)** will be curatively treated with piperaquine (960mg) and **group 2 and 4 (LD-PIP/LD-PIP2/SP)** with sulfadoxine-pyrimethamine (1000mg/50mg). All volunteers will receive a final treatment (ET) according to national guidelines with Malarone® on day 36 to assure the radical clearance of all parasite stages. In case a volunteer remains 18S qPCR and Pfs25 qRT-PCR negative for 7 days after T1 or T2, final treatment with Malarone® will also be initiated and end of study will apply for the volunteer. The exact number of site visits and blood examinations per volunteer depends on the time to positive 18S qPCR above 5000 parasites/ml and potential recrudescence - with a maximum number of 45 study visits and a maximum of 500 mL collected blood. Periodical physical examinations will be performed and the subject is asked to complete a diary. In addition three direct skin feeds (DFA) will be performed with one cup of 20-30 mosquitoes, allowing mosquitoes to feed on the volunteer's forearms. The duration of subject participation will be 51 days from day of challenge, following a screening period of up to 120 days.

1. INTRODUCTION AND RATIONALE

1.1. Introduction

Malaria is one of the most devastating infectious diseases worldwide. Despite all the progress that has been made in reducing the burden of malaria by the up-scaling of protective measures and efficacious treatment(6), in 2015 there were still 212 million cases and 429,000 deaths, particularly of children less than five years of age(1). In addition to the appalling human suffering, this disease forms a profound economic burden for the affected countries, which are already struggling with poverty. The urgency of the situation is further emphasized by the waning effectiveness of all currently registered anti-malarials due to fast emergence and spread of resistance and the absence of an effective vaccine(2, 7).

The World Health Organization (WHO) has declared malaria control a global development priority and has changed their recommendation from control programs to eradication programs. It is widely accepted that malaria eradication is unlikely to be attainable with the currently available tools(6, 8).

The major challenge for malaria elimination is the highly efficient spread of malaria parasites. Human malaria is caused by protozoa of the genus *Plasmodium*: *P. falciparum*, *P. vivax*, P. *ovale*, *P. malariae*, and *P. knowlesi*. Parasites (sporozoite stage) are injected into the skin by an infected female *Anopheles* mosquito. After penetration of the skin capillaries, sporozoites are transported to the liver, where they develop and multiply in liver cells before release into the blood (merozoite stage) and invading red blood cells for further maturation and multiplication. The cyclical proliferation of asexual stages within the human red blood cells is responsible for the occurrence of clinical symptoms. A small fraction of asexual stages is committed to enter sexual development. The formation of male and female gametocytes are essential for parasite transmission via the female *Anopheles* mosquito vector. Circulating gametocytes do not cause clinical pathology or symptoms. The circulating gametocytes therefore have no clinical consequences but play an essential role in the onward transmission of malaria infections. The renewed focus on malaria elimination has increased the priority of research towards development of interventions to block malaria transmission, including transmission blocking vaccines (TBVs). By interrupting transmission of malaria parasites in mosquito vectors, a reduction in the number of secondary infections in the community is expected. TBVs will play an important role in complete arrest of malaria transmission in endemic areas(4, 9). Similarly, a number of gametocidal and/or sporontocidal drug candidates have been generated in recent years(10). From a community perspective, deployment of transmission-blocking drugs and TBVs will be an efficient complementary element in an integrated program of anti-malarial interventions, particularly for malaria elimination.

1.2. Rationale

Controlled human malaria infection (CHMI) studies have become a safe(11-13) and widely accepted model for evaluating the efficacy of vaccines(14), anti-malarial drug candidates(15-17), diagnostic assays(18) and assessment of immunologic responses(19, 20). These studies provide a cost-effective and fast way to circumvent the use of large-scale field efficacy studies for down selection of intervention candidates. However, an effective model to evaluate interventions to block malaria transmission is currently lacking. In this project we aim to develop a controlled human malaria infection transmission model ("*CHMI-trans*") or "challenge model" to evaluate the capacity of vaccines, biologics (monoclonal antibodies, or mAbs), and drugs to block malaria parasite transmission by assessing infectiousness of *Plasmodium falciparum* (*Pf*) gametocyte carriers for

Anopheles mosquitoes. This study is a sequel on CHMI-trans1 for the optimization of the transmission model.

The first step of malaria parasite transmission from humans to *Anopheles* mosquitoes, is the generation of mature gametocytes in the human peripheral blood. Gametocytes are non-pathogenic malaria life stages and typically comprising less than 5 percent of the total parasite population prior to treatment. Once treatment is initiated, gametocyte production ceases abruptly (e.g. artemisinins) or is tolerated or even stimulated as part of a terminal investment of malaria parasites under drug pressure (e.g. sulfadoxine-pyrimethamine and piperaquine)(5, 21, 22). Importantly, malaria transmission is not prevented by currently used antimalarial drugs or the recently proposed malaria vaccine RTS,S. Malaria TBVs and drugs can interrupt parasite development in the mosquito and thereby play a central role in malaria elimination efforts and in efforts to contain the spread of drug resistant malaria(4, 23). However, for downstream selection and clinical development, there is currently an uncertain association between the methods used for preclinical and early clinical evaluation of candidate drugs/vaccines (e.g., the standard membrane feeding assay, or SMFA) and their ultimate field deployment, where the infectivity of naturally exposed hosts forms a key outcome measure. The standard membrane feeding assay (SMFA) can determine the percentage of the transmission-reducing activity (TRA) of human serum by feeding Anopheles mosquitoes on human blood with cultured gametocytes, mixed with experimental sera. Presently, we are heavily reliant on the SMFA to inform the early clinical efficacy stage-gate; however, this assay has not been accurately calibrated against the most widely used ex vivo assessment of gametocyte infectiousness, the direct membrane feeding assay (DMFA)(24). Therefore, its predictive value in assessing interventions that block human-to-mosquito transmission remains uncertain(3).

In order to use the CHMI model for the evaluation of transmission blocking interventions, current CHMI challenge methods that abort asexual blood-stage parasites prior to emergence of sexual-stage gametocytes need to be modified to optimize gametocyte development and thus transmission to *Anopheles* mosquitoes. Importantly, preliminary data show the presence of gametocytes in the CHMI volunteers after piperaquine treatment, with undetermined infectivity for mosquitoes(25).

Establishment of the *CHMI-trans* model will facilitate effective bridging of SMFA to the direct membrane feeding assay (DMFA) and direct skin feeding assay (DFA) (such as via the evaluation of mAbs with defined transmission-blocking activity), to support the future positioning of SMFA as a more informative tool for predicting clinical outcomes. Most importantly, successful completion of the proposed work will fill a critical unmet need by making available a more biologically relevant assay to rapidly and cost effectively assess transmission-blocking interventions during early clinical development.

The primary objective of this study is to assess gametocytemia and mosquito transmissibility in volunteers after parasite blood stage- or sporozoite challenge.
We have previously shown in CHMI-trans1 that gametocytemia can safely be induced in participants through a malaria sporozoite challenge followed by subcurative regimens of piperaquine (PIP) and sulfadoxine-pyrimethamine (SP), at a treatment threshold of 5000

parasites/mL or a positive thick smear, with incidental evidence for mosquito infection (*Reuling et al* 2017). In another study with infection after blood-stage inoculum, higher gametocytemia was achieved and transmission to mosquito was observed in 73% (8/11) of the participants with mosquito infection rates of 2-17% (*Collins et al* 2017). The combined data provide clear evidence for the safe and reproducible induction of gametocytes at densities infectious to mosquitoes, and thereby providing first evidence for a successful CHMI transmission model. These studies show that gametocyte densities are strongly associated with the preceding densities of the asexual progenitors. Therefore, studies on the evaluation of transmission-blocking interventions (TBIs) will be optimized aiming to maximize gametocyte densities and the area under the curve (AUC) of gametocytemia by increasing duration and load of the asexual parasite burden (asexual AUC) without compromising the safety of the participants. This proposed study will evaluate different drug regimens based on the results of the CHMI-trans1 study, and compare the difference between a mosquito- and blood stage- challenge.

1.3. Clinical Experience

There is a large clinical experience with infecting humans by the bite of *P. falciparum* sporozoite-infected mosquitoes and blood stage inoculum of *P. falciparum*. These challenge trials have become highly standardized(26, 27). The first human malaria challenge study was performed in 1917, and since 1986, when the modern protocol using laboratory adapted *P. falciparum* strains was first performed by the US army, >3,500 subjects have been challenged by the bites of mosquitoes fed on cultures of *P. falciparum* gametocytes to produce sporozoites(28). Infecting humans by the bite of *P. falciparum* sporozoite-infected mosquitoes and blood stage inoculum are established clinical trial methodologies and is considered a reproducible, predictable and safe method of inducing *P. falciparum* malaria. The results of such studies were summarized in 1997 (11), in 2007 (29) and in 2012 (30) (27).

The Radboud university medical center (Radboudumc) has the experience and infrastructure to conduct CHMI trials. Over 350 subjects have undergone CHMI in studies conducted by Radboudumc since 2001. Standard operating procedures according to international standards are in place for both clinical and laboratory activities, and the Radboudumc mosquito and parasite culture was positively audited in 2014 by an international independent auditor. Radboudumc has developed a sensitive method of parasite detection by real-time quantitative PCR (18S qPCR) in whole blood that allows us to detect malaria infection in an early stage and is able to detect small differences in parasite density(31). We further pioneered the assays for molecular detection of low densities of gametocytes(32) and sexing of gametocytes(33) as parameters for the transmission from humans to mosquitoes(34).

1.4. Safety

After CHMI most malaria-naive volunteers experience symptoms such as headache, chills or fatigue during 1-3 days. During the extensive experience with CHMI, severe or life-threatening malaria has never been reported.

In the twenty-three CHMI studies conducted at Radboudumc (involving 376 healthy volunteers), three cardiac events have occurred after infection with the NF54 strain with the confirmed or differential diagnosis of myocarditis (35, 36). The occurrence of myocarditis in both natural uncomplicated malaria and CHMI has not been described elsewhere. However, the three cardiac events share no specific factor other than malaria infection.

With regards to the malaria infection, these myocarditis cases do share a number of characteristics: i) they took place 1-5 days after start of antimalarial treatment but different anti-malarial drugs were used in all cases ii) there was no parasitemia detectable at the time of the event, and iii) next to the *P. falciparum* infection, other and known possibly triggering factors (e.g. preceding vaccinations, concomitant infections, cannabis use) were present during or preceding the event. The three cases are discussed in detail in section 14.

As a result of these cardiac SAEs our safety procedures for CHMI have been strongly intensified. In the current trial, we will adhere to those stringent procedures that are relevant, including:

1. Individuals are excluded from participation if they have first or second degree relatives who had cardiovascular events (including ischemic events and myocarditis) under the age of 50
2. A positive urine toxicology test for amphetamines, cocaine and cannabis is an exclusion criterion
3. Increased control of hs troponin T as a marker of cardiac damage; treatment with Malarone® is initiated in consultation with the cardiologist.
4. Daily measurements of platelets; volunteers will be treated with T1 when platelet levels are <120x 10^9/L and T3 when platelet levels are <50x 10^9/L.
5. Agreement by the volunteer to refrain from intensive physical exercise (disproportionate to the subject's usual daily activity or exercise routine) during the malaria challenge period.

Transient, asymptomatic liver function derangements have been reported in volunteers in previous CHMI studies, and are likely to be related to the challenge infection (*Reuling et al. manuscript in preparation*). A retrospective analysis of 13 CHMI studies conducted in the Radboudumc showed that 72/120 (60%) of the volunteers that were treated at thick smear parasitemia levels have mild (38%), moderate (10%) or severe (12%) increases of liver transaminases (ALT/AST). The liver function test (LFT) elevations remained asymptomatic in all the affected volunteers, and are typically not associated with bilirubin changes. Detailed analysis on the LFT elevations pattern showed that these elevations exceeded the upper limit of normal (ULN) around the day of treatment with antimalarials to 2 days after treatment. The peak LFTs elevations are found between 2-14 days after treatment with values normalized at study end (35–42 days after challenge infection). In some volunteers, the elevations were also found associated with lactate dehydrogenase, suggesting that associated sub-clinical haemolysis might have been present during challenge.

Volunteers treated based on qPCR threshold of 100 *Pf*/mL showed a lower percentage, and severity of LFT abnormalities. 13/58 volunteers (22%) showed LFT derangements, with 11/58 (19%) mild, 1/58 (2%) moderate, and 1/58 (2%) severe abnormalities. Importantly, all volunteers showed normalized values at study end.

While severe/complicated malaria is clearly associated with liver damage with sequelae after natural infection (*WHO Severe Malaria , Tropical Medicine and International health 2014)*, such severe complications do not seem to occur in naturally acquired uncomplicated clinical malaria (*Abro et al. J Coll Physicians Surg Pak. 2009 and Ghoda et al. Trop Gastroenterol. 2002*). In this context it is important to note that parasite densities under these conditions are several logs higher than observed in the CHMI model.

A clear explanation for the transient elevated transaminases in our CHMI studies is not obvious. Although higher parasitemia associates with higher chances of LFT elevations on group level, there is no clear relationship on individual levels. Given the diversity of drug regimens used, it also seems unlikely to be directly related to anti-malarials. Similarly, the timing, differential, and limited use of paracetamol does not support a clear relationship. Rather a combination of the above mentioned factors, and individual susceptibility may have triggered the observed abnormalities.

Following an independent review of the safety committee, and a hepatology expert, it was concluded that these transient asymptomatic and severe LFT elevations are most likely a direct consequence of the malaria infection rather than reflecting a direct drug-induced liver injury caused by the antimalarial drug or other liver toxin. Given the rapid and spontaneous resolution of the observations and the absence of significantly elevated bilirubin these events were judged to not cause permanent subclinical liver damage, and to not preclude further studies utilising *Plasmodium falciparum* challenge.

As a result of the LFT elevations our safety procedures for CHMI have been intensified. In the current trial, we will adhere to those stringent procedures that are relevant, including:

1. Exclusion of subjects with abnormal ALT/AST values on baseline
2. Upon inclusion: Avoid additional triggers that may cause ALT/AST elevations
   a. Alcohol intake from baseline up to 1 week post-treatment
   b. Heavy physical exercise
   c. Recreational drug use
   d. >3 grams/ day of paracetamol/acetaminophen
3. Standard monitoring of LFT at day* *C-1; C+6; C+8; C+10; T; T+2; T+4; T+6; EOS*
4. Initiation of subcurative anti-malarial treatment at ALT/AST levels >2.5xULN (prior T1)
5. Initiation of a curative anti-malarial treatment at ALT/AST levels >5x ULN (prior T1)

*C = challenge; T = day of treatment 1,2, and 3; EOS = end of study

2. OBJECTIVES

Primary objectives:

1) To evaluate the safety of CHMI-trans protocols in healthy malaria-naïve volunteers challenged with *Plasmodium falciparum* by sporozoite challenge and blood stage challenge.
2) To assess gametocyte infectiousness for *Anopheles* mosquitoes through mosquito membrane feeding assay (Direct Membrane Feeding Assay, DMFA).

Secondary objectives:

3) To determine the dynamics of gametocyte commitment, maturation and sex ratio by molecular markers of sexual stage development.
4) To determine the time-point of peak density of gametocytemia in the CHMI-trans model.
5) To assess gametocyte infectiousness for *Anopheles* mosquitoes through mosquito feeding assay (Direct Skin Feeding Assay, DFA).

Exploratory Objectives (see section 9.1.3 for detailed endpoints):

6) To determine gametocyte fitness by molecular markers, and *ex vivo* assessments of gamete formation and fertilization.
7) To analyse immune responses in volunteers exposed to a *Plasmodium falciparum* challenge infection
8) To explore associations between elevated transaminases and inflammatory biomarkers.

3. STUDY DESIGN

This study will be a single center, open label clinical trial. A total of 24 volunteers, in two cohorts (n=12), will be randomly assigned to two groups per cohort (n=6). **Cohort A** will be subjected to a standard controlled human malaria infection (CHMI) delivered by five *Pf*-infected mosquitoes (groups 1 and 2). **Cohort B** will be subjected to a standard blood stage challenge with ~2,800 *Pf*-infected erythrocytes by intravenous injection (groups 3 and 4) (see Figure 1, section 3).
Treatment is subsequently initiated to induce gametocytemia (treatment 1, T1) and to clear pathogenic asexual parasites whilst leaving gametocytes unaffected (treatment 2 and 3, T2 and T3). At the end of the study, treatment of all parasite stages is provided following national treatment guidelines (end treatment, ET).
Once malaria infections are detected by 18S qPCR positive at a density of 5,000 par/ml (sporozoite challenge) or on day 8 (blood stage challenge), all volunteers will be treated with a single oral subcurative low-dose of piperaquine (LD-PIP, 480 mg, T1). Daily blood samples will allow detailed quantification of gametocytes and gametocyte sex ratio. Using blood samples taken twice daily, the initial clearance of parasitemia will be carefully monitored. After T1, volunteers will receive a second treatment (T2, LD-PIP2, 480mg) if a recrudescence of asexual parasitemia (exceeding 1,500 par/ml by 18S qPCR) occurs before day 21 post challenge infection. On day 21 or when a recrudescence occurs after T2, volunteers in **group 1 and 3 (LD-PIP/LD-PIP2/PIP)** will be curatively treated with piperaquine (960mg) and **group 2 and 4 (LD-PIP/LD-PIP2/SP)** with sulfadoxine-pyrimethamine (1000mg/50mg) (see section 9.3.2. for detailed treatment criteria). These treatment regimens cure asexual parasitemia while leaving immature and mature gametocytes unaffected(5). To ensure the radical clearance of all parasite stages, all volunteers will receive a final treatment (ET) according to national guidelines with atovaquone/proguanil (Malarone®) on day 36. In case a volunteer remains 18S qPCR and Pfs25 qRT-PCR negative for 7 days after T1 or T2, final treatment with Malarone® will also be initiated and end of study will apply for the volunteer. On the day of administration of T3 and three days after initiation of T3, venous blood samples will be obtained for Direct Membrane Feeding Assay (DMFA). An additional blood sample will be taken for DMFA between day 7-21 after T3, the exact time-point being based on the density of gametocytemia as measured by Pfs25 qRT-PCR. In addition, on the DMFA days volunteers will be subjected to Direct Skin Feeding Assays (DFA) with one cup of 20-30 mosquitoes, allowing mosquitoes to feed on the volunteer's forearms. These assays will provide evidence on the infectivity of volunteers at these time-points.
If a planned volunteer (screened and eligible for participation) is subsequently unable to participate or becomes ineligible for enrollment at the inclusion visit, or before challenge on day 0, she/he will be replaced by another volunteer enrolled at the inclusion visit. For this purpose, two to four additional volunteers (screened and eligible for participation) will be identified and enrolled at the inclusion visit to act as back-ups.

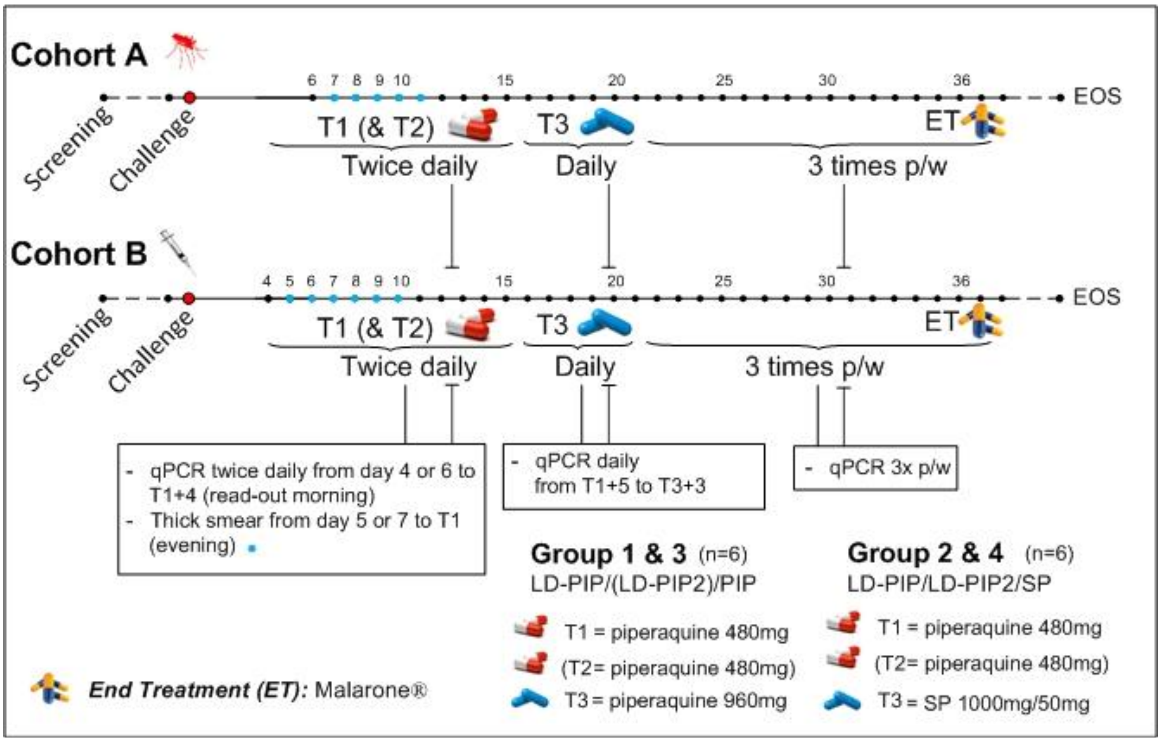

Figure 1 – Study design. After the initial challenge, data from 175(30) volunteers indicate the first possible moment of parasitemia to be at day 6 and the first positive thick blood smear between day 7-14.5 for the sporozoite challenge and day 8 for the blood stage challenge. Volunteers will be monitored twice daily from day 6 (sporozoite challenge) or day 4 (blood stage challenge) onwards until malaria parasites are detected at 5000 p/mL or on day 8 for blood stage challenge, upon which they are treated (T1). After T1, volunteers will receive a second treatment (T2, LD-PIP2, 480mg) if a recrudescence of asexual parasitemia (exceeding 1,500 par/ml by 18S qPCR) occurs before day 21 post challenge infection. On day 21 or once parasites density reach 1500 p/mL after T2, volunteers will receive a curative regimen (T3). After T3, volunteers will visit the study site 3 times a week until final treatment according to national guidelines with Malarone® on day 36, to assure radical clearance of all parasite stages.

4. STUDY POPULATION

4.1. Population (base)

The study population will be comprised of adult male and female healthy, malaria naïve subjects. A total of 24 subjects will be enrolled to participate in the study and be randomized to two groups per cohort (n=6 per group).. The investigator will ensure that all subjects being considered for the study meet the following eligibility criteria. Subject eligibility is to be established and confirmed by checking through all inclusion/exclusion criteria at both screening and baseline. A relevant record (e.g. checklist) of the eligibility criteria will be stored with the source documentation at the study site.

4.2. Inclusion criteria

In order to be eligible to participate in this study, a subject must meet all of the following criteria:

1. Subject is aged ≥ 18 and ≤ 35 years and in good health.
2. Subject has adequate understanding of the procedures of the study and is able and willing (in the investigator's opinion) to comply with all study requirements.
3. Subject is willing to complete an informed consent questionnaire and is able to answer all questions correctly.
4. Subject is able to communicate well with the investigator and is available to attend all study visits, lives in proximity to the trial centre (<10 km) or (if >10km) is willing to stay in a hotel close to the trial centre during part of the study (from day 4 (blood stage challenge) 5 (sporozoite challenge) post-infection until T1+4 provided that the subject has had 2 consecutive negative 18S qPCR tests (at least 24 hours apart) following T1 treatment; or until day T3+3).
5. The subject will remain within the Netherlands during the challenge period, will not travel to a malaria-endemic area during the study period, and is reachable (24/7) by mobile telephone throughout the entire study period.
6. Subject agrees to their general practitioner being informed and contacted about their participation in the study and agrees to sign a form to request the release by their General Practitioner (GP), and medical specialist when necessary, to the investigator(s), of any relevant medical information concerning possible contra-indications for participation in the study.
7. The subject agrees to refrain from blood donation to Sanquin or for other purposes throughout the study period and for a defined period thereafter according to current Sanquin guidelines.
8. For female subjects: subject agrees to use continuous adequate contraception** and not to breastfeed for the duration of study.
9. Subject agrees to refrain from intensive physical exercise (disproportionate to the subject's usual daily activity or exercise routine) during the malaria challenge period until day 38 after infection.
10. Subject agrees to avoid additional triggers that may cause elevations in liver enzymes including alcohol from baseline up to 1 week post treatment (T3).
11. Subject has signed written informed consent to participate in the trial.

*(*Acceptable forms of contraception include: established use of oral, injected or implanted hormonal contraceptives; intrauterine device or intrauterine system; barrier methods (condoms or diaphragm with additional spermicide); male partner's sterilisation (with appropriate post-vasectomy documentation of absence of sperm in the ejaculate); true abstinence when this is in line with the preferred and usual lifestyle of the subject; Periodic abstinence (e.g., calendar, ovulation,*

symptothermal, post-ovulation methods) and withdrawal are not acceptable methods of contraception.)

4.3. Exclusion criteria

A potential subject who meets any of the following criteria will be excluded from participation in this study:

1. Any history, or evidence at screening, of clinically significant symptoms, physical signs or abnormal laboratory values suggestive of systemic conditions, such as cardiovascular, pulmonary, renal, hepatic, neurological, dermatological, endocrine, malignant, haematological, infectious, immunodeficient, psychiatric and other disorders, which could compromise the health of the volunteer during the study or interfere with the interpretation of the study results. These include, but are not limited to, any of the following.
   - 1.1. Body weight <50 kg or Body Mass Index (BMI) <18 or >30 kg/m2 at screening.
   - 1.2. A heightened risk of cardiovascular disease, as determined by: an estimated ten year risk of fatal cardiovascular disease of ≥5% at screening, as determined by the Systematic Coronary Risk Evaluation (SCORE); history, or evidence at screening, of clinically significant arrhythmia's, prolonged QT-interval or other clinically relevant ECG abnormalities; or a positive family history of cardiac events in 1st or 2nd degree relatives <50 years old.
   - 1.3. A medical history of functional asplenia, sickle cell trait/disease, thalassaemia trait/disease or G6PD-deficiency.
   - 1.4. History of epilepsy in the period of five years prior to study onset, even if no longer on medication.
   - 1.5. Screening tests positive for Human Immunodeficiency Virus (HIV), active Hepatitis B Virus (HBV), Hepatitis C Virus (HCV)
   - 1.6. Chronic use of i) immunosuppressive drugs, ii) antibiotics, iii) or other immune modifying drugs within three months prior to study onset (inhaled and topical corticosteroids and oral anti-histamines exempted) or expected use of such during the study period.
   - 1.7. Any recent or current systemic therapy with an antibiotic or drug with potential anti-malarial activity (chloroquine, doxycycline, tetracycline, piperaquine, benzodiazepine, flunarizine, fluoxetine, tetracycline, azithromycin, clindamycin, erythromycin, hydroxychloroquine, etc.) (allowable timeframe for use at the Investigator's discretion).
   - 1.8. History of malignancy of any organ system (other than localized basal cell carcinoma of the skin), treated or untreated, within the past 5 years.
   - 1.9. Any history of treatment for severe psychiatric disease by a psychiatrist in the past year.
   - 1.10. History of drug or alcohol abuse interfering with normal social function in the period of one year prior to study onset, positive urine toxicology test for cocaine or amphetamines at screening or at inclusion, or positive urine toxicology test for cannabis at inclusion.

2. For female subjects: positive urine pregnancy test at screening and/or at the baseline visit.
3. Abnormal ALT/AST values on baseline
4. Any history of malaria, positive serology for *P. falciparum*, or previous participation in any malaria (vaccine) study.
5. Known hypersensitivity to or contra-indications (including co-medication) for use of sulfadoxine-pyrimethamine, piperaquine, chloroquine, Malarone®, artemether-lumefantrine, primaquine or history of severe (allergic) reactions to mosquito bites.

6. Participation in any other clinical study in the 30 days prior to the start of the study or during the study period.
7. Being an employee or student of the department of Medical Microbiology of the Radboudumc or the department of Internal Medicine.
8. Any other condition or situation that would, in the opinion of the investigator, place the subject at an unacceptable risk of injury or render the subject unable to meet the requirements of the protocol.
9. For cohort B (blood stage challenge): Received a blood transfusion in the past.
10. For cohort B (blood stage challenge): Women of childbearing potential with a screening test positive for erythrocyte anti-Rh(c) and/or anti-Rh(e) antibodies.

5. SAMPLE SIZE CALCULATION

In this trial, we will determine the suitability of our treatment strategies to induce gametocytemia, defined as gametocyte prevalence by Pfs25 qRT-PCR on any moment during follow-up and to achieve infectiousness to mosquitoes. Based on preliminary data, we expect >95% individuals will develop gametocytes after commencing treatment with sulphadoxine-pyrimethamine(37) or piperaquine(25). In CHMI-trans1, 100% (16/16) individuals developed gametocytes. The CHMI-trans approach is considered unsuitable if <50% of individuals develop mature gametocytes; the lower limit of the confidence interval around the proportion of gametocyte positive individuals should thus be above 50%. If we enroll 12 individuals of whom 11 become gametocyte positive, the lower limit of the 99% confidence interval around this proportion is 52%. We anticipate that ~73% of the individuals who are exposed to Pf by blood stage inoculation will infect at least one mosquito (Collins, unpublished data). Within each of the inoculation means (mosquito inoculation versus bloodstage, 12 participants each) we may thus expect 8 or 9 infectious individuals and consequently a lower limit of the 95% confidence interval around the proportion of infectious individuals ≥34%.

Comparisons between groups are underpowered by conventional frequentist approaches and a Bayesian model will be used to select the challenge and treatment strategy with evidence for highest gametocyte prevalence, density, infectiousness prevalence and mosquito infection rates.

TREATMENT OF SUBJECTS

5.1. Investigational product/treatments (for T1,T2, and T3)

All volunteers will receive a subcurative treatment (T1, LD-PIP, 480mg) with piperaquine when the first treatment criterion occurs (see section 9 Methods). Volunteers with a recrudescence before day 21 post challenge infection will receive a second treatment with piperaquine (T2, LD-PIP2, 480mg). On day 21 or when a recrudescence occurs after T2, volunteers in **group 1 and 3 (LD-PIP/LD-PIP2/PIP)** will be curatively treated with piperaquine (960mg) and **group 2 and 4 (LD-PIP/LD-PIP2/SP)** with sulfadoxine-pyrimethamine (1000mg/50mg). Volunteers treated with piperaquine will receive 160mg tablet strength as piperaquine phosphate (Penn Pharmaceutical Services Limited UK) for the subcurative or curative regimen. Volunteers treated with sulfadoxine-pyrimethamine will receive two tablets of 500mg/25mg tablet strength (Amstelfarma NL) for the curative regimen.
Refer to section 6 for further details regarding piperaquine and sulfadoxine-pyrimethamine. See section 9.3.2. for detailed treatment criteria.

5.2. Investigational product Use of co-intervention

All volunteers will receive a final treatment (ET, end treatment) with Malarone® after CHMI as described in section 9. The following list of concomitant medications that are contraindications to Malarone® is based on the Malarone® product information sheet.
Concomitant medications:

- Use of anticoagulants (warfarin and other coumarin based anticoagulants)
- Use of Rifampicin, Rifabutin, Tetracycline or Metoclopramide
- Use of Indinavir

5.3. Escape medication (if applicable)

Volunteers may be advised to take tripelennamine crème for the local treatment of mosquito bites. Volunteers are advised to take paracetamol for complaints secondary to the CHMI (fever, muscle aches, headache, etc.). Tripelennamine crème, paracetamol or any other symptomatic treatment will be supplied to the volunteers. The maximum dose of paracetamol
in this study is 3 grams a day.

Pre-emptive rescue treatment with Malarone® can commence whenever deemed necessary
by the investigator.

6. INVESTIGATIONAL PRODUCT (TREATMENT PRODUCT)

6.1. Name and description of investigational products

- **Piperaquine** tablets (160mg tablet strength as piperaquine phosphate, Penn Pharmaceutical Services Limited UK), administered orally.
  - subcurative regimen (480 mg, 3 tablets of160mg)
  - curative regimen (960 mg, 6 tablets of 160mg).
- **Sulfadoxine-pyrimethamine** tablets (Amstelfarma, NL), administered orally.
  - curative regimen (1000mg/50mg as 2 tablets).

6.2. Summary of findings from non-clinical studies

Please see Summary of Product Characteristics (SPC) of Fansidar® (the original proprietary name of the generic drug sulfadoxine-pyrimethamine used in this study) and the Investigator's Brochure for piperaquine.

6.3. Summary of findings from clinical studies

Please see Summary of Product Characteristics (SPC) for Fansidar® (the original proprietary name of the generic drug sulfadoxine-pyrimethamine used in this study) and the Investigator's Brochure for piperaquine for detailed information. A short summary of the available data is given below.

6.3.1. Clinical data for sulfadoxine-pyrimethamine

A fixed-dose combination of sulfadoxine and pyrimethamine, the usual successor to failing chloroquine, has been widely implemented in the last decade and is one of the most widely used antimalarial treatments in the world. Sulfadoxine-pyrimethamine (SP) has the great advantage that the entire treatment can be given as a single dose(37).

Although artemisinin-combination therapy has now replaced the use of SP throughout malaria-endemic settings, SP is still widely used as a first-line intermittent preventive treatment of malaria in pregnancy (IPTp) (38-40) and for malaria seasonal chemoprophylaxis alone or in combination with amodiaquine(41).

Sulfadoxine-pyrimethamine can effectively block two enzymes involved in the biosynthesis of folinic acid within the parasites: sulfadoxine inhibits the dihydropteroate synthetase and pyrimethamine blocks the dihydrofolate reductase. Both drugs are active predominantly against the later development stages of asexual parasites(42). The curative regimen consists of 2-3 tablets of 500mg sulfadoxine and 25mg pyrimethamine per tablet(42).

Some studies show that treatment with SP is correlated with an increase in gametocyte carriage(43, 44).

6.3.2. Clinical data for Piperaquine

Piperaquine is a bisquinoline 4-aminoquinoline anti-malarial structurally related to chloroquine. It was synthesized independently in France and China in the 1960s(45, 46), and widely used for malaria control activities in China in the 1970's and 1980's (13). In the 1990s, piperaquine was reconsidered as a partner drug in artemisinin-based combination therapy, and the renewed development led to a novel combination formulation of dihydroartemisinin plus piperaquine, each tablet containing 40mg dihydroartemisinin and 320mg piperaquine phosphate (DHA-PQP). The mechanism of action and of

resistance of piperaquine has not been well studied but is likely to be similar to those of drugs of the same class(47). The antimalarial activity of piperaquine when administered as a single agent in a malaria challenge model has been established at QIMR Berghofer Medical Research Institute, Australia(25), in a dose finding study. Administered as a single dose (960, 640 and 480 mg) the drug rapidly cleared asexual parasitemia. Recrudescences have occurred in 3 out of the 6 subjects treated with 480mg piperaquine, therefore a second dose of 480mg of piperaquine will be given if this occurs, and thus a curative dose of piperaquine (960 mg) in this study will be given when a second recrudescence occurs or on day 21.(25).

6.3.3. Pharmacokinetics and Metabolism of sulfadoxine-pyrimethamine

Both sulfadoxine and pyrimethamine are readily absorbed from the gastrointestinal tract after oral administration. Both drugs are highly bound to plasma protein (about 90%). Sulfadoxine has an elimination half-life of 4.9-13.2 days and pyrimethamine, 2.1-6.5 days(37). Peak plasma levels of sulfadoxine and pyrimethamine ranging from 51-76 mg/L are reached in approximately 4 hours (range 1.5-8 hours) and 0.13-0.4 mg/L within 2.1-77 hours, respectively, after a single tablet of 500mg/25mg(42). Pyrimethamine has a larger volume of distribution than sulfadoxine and is concentrated in kidneys, lungs, liver and spleen. Sulfadoxine and pyrimethamine are mainly metabolized by the liver. Sulfadoxine undergoes various degrees of acetylation, hydroxylation and glucuronidation. Both drugs are excreted mainly through the kidneys(42).

6.3.4. Pharmacokinetics and Metabolism of Piperaquine

The pharmacokinetic properties of piperaquine are similar to those of chloroquine. It has a very large volume of distribution, ranging from 103 to 716 l/kg, values that are significantly larger even than comparable drugs such as chloroquine(48). It has a very long terminal elimination half-life, 531 h (22 days) and 468 h (20 days) in adults and children, respectively(49). The prolonged half-life results in a beneficial post-treatment prophylactic period, estimated to be about 20 days, and protecting against both *P. vivax* and *P. falciparum*. Although early recurrent infections are reduced, infections treated with DHA-PQP are more likely to produce gametocytes than artemether-lumefantrine (AL), an observation hypothesized to reflect the lower dosing of artemisinin derivative in DHA-PQP (total ~7.5mg/kg of DHA compared to ~11.5mg/kg of artemether in AL). Furthermore, a smaller volume of distribution, and shorter half life of piperaquine is seen in children, resulting in a higher risk of recrudescence and earlier re-infection. Thus, an increase of the weight-adjusted dosage in young children may be required(50).

Piperaquine is highly lipophilic, and its oral bioavailability is approximately doubled by administration with a high-fat meal(51, 52). However, data regarding the influence of food on the bioavailability of piperaquine in human subjects are conflicting(48, 53, 54). In a study carried out in Papua New Guinea, a surprisingly low efficacy of DHA-PQP was reported (88% at day 42), significantly lower than that for artemether–lumefantrine (AL). However, the difference had wide confidence intervals and was apparent at day 28 but not at day 42. This reduced efficacy is in contrast to other studies carried out in Africa(55-57) and Asia(58, 59) where DHA-PQP had similar or higher efficacy to other ACTs. Because of apparent significant effects of food intake, piperaquine will be administered in a fasting state in this study.

6.3.5. Safety and tolerability of sulfadoxine-pyrimethamine

Sulfadoxine-pyrimethamine is well tolerated, with the main adverse effects reported to be gastroinstestinal disturbances, headache, dizziness and skin reactions such as photosensitivity, rash, pruritus, urticaria and slight hair loss, which are mainly associated with sulfanomides(39, 60, 61). In very rare cases, particularly in hypersensitive patients, severe, possibly life-threatening skin reactions such as erythema multiforme, Stevens-Johnson syndrome and Lyell's syndrome may occur. The drug should be withdrawn immediately if skin reactions are observed. Furthermore, leukopenia, thrombocytopenia, megaloblastic anaemia, haemolytic anaemia, haematuria, oliguria and hepatitis have also been reported. Patients should be advised that sore throat, fever, cough, dyspnoea or purpura may be the first signs of serious side effects. The intake must be stopped immediately at the first signs of skin eruptions, a significant decrease of blood cells, or a bacterial or fungal superinfection(42).

6.3.6. Safety and tolerability of Piperaquine

Piperaquine is well tolerated both in adults and in children(49), with the main adverse events reported to be gastrointestinal disturbance such as diarrhoea(58), although this varies considerably according to geographical region. Electrocardiographic effects of piperaquine have been specifically evaluated in two studies(59, 62-64). Both demonstrated a prolongation of the corrected QT interval during treatment (between 11 and 14ms). Very few individual patients experienced a prolongation that could be regarded as clinically significant (>60ms); notably, QTc prolongation induced by piperaquine has not been associated with clinically relevant cardiovascular events suggesting a pro-arrythmogenic effect. Therefore, although statistically significant, the QTc prolongation observed following piperaquine therapy is unlikely to be clinically relevant. European regulatory authorities have however recommended that DHA-PQP should not be administered with food (to reduce peak concentrations), and caution that prior and post electrocardiographic monitoring should be undertaken, as well as avoidance of concomitant consumption or recent exposure to drugs at risk of QTc prolongation(63, 64).

The principal risks related to piperaquine include:

- Mild elevations in hepatic enzymes; transaminase elevations in malaria patients have typically been <2xULN, with no increases >5xULN, and with no severe liver function derangements (Hy’s law cases) observed. The pattern of transaminase increases is not unusual with acute malaria, although there was a suggestion of a potentially dose-related effect.
- QTc prolongation (both QTcB and QTcF); although mostly in the range >30 msec but <60 msec, prolongations >60 msec have been observed with a single instance of QTcF that exceeded 500 msec. This risk is mitigated by administering the drug while the subject is fasting.

6.4. Drug and dose selection rationale

The rationale for the use of sulfadoxine-pyrimethamine and piperaquine as treatment is based on the fact that they are the only drugs known to increase gametocytes in patients (23, 50, 65). Both drugs do not kill circulating gametocytes in contrast to other antimalarial drugs (e.g. artemisinins), which would not allow addressing the current study objectives.

The association between gametocyte production and transmissibility after sulfadoxine-pyrimethamine (SP) is complex. High gametocyte concentrations have been observed after treatment with SP, however, the infectivity of these gametocytes upon their appearance is lower.

Possibly, because the SP treatment initiates an efflux of less mature gametocytes that require 3-7 additional days of maturation(66), and the presence of sulfadoxine-pyrimethamine in the bloodmeal of the mosquitoes(65). Nevertheless, the considerable increase in the prevalence and density of gametocytes results in a net increase of infectivity to mosquitoes although this increase may be lower than expected based on the gametocyte concentration (the chance of mosquito infection per gametocyte is smaller).
The selection of the optimal treatment for induction of infectious gametocyte requires a thorough comparison, and possibly a combination of agents wherein the production and fitness of gametocytes are optimized. The reason to use two study arms per cohort in this study is to directly compare the possible combinations of piperaquine and sulfadoxine-pyrimethamine, and increase the likelihood of obtaining the study-objectives.

The rationale to use suboptimal treatment (T1 and T2) regimens is based on partial clearance of asexual parasites followed by recrudescence. This will presumably cause an increase in asexual commitment to gametocytes through terminal investment of malaria parasites under drug pressure (5, 21) (22).
In previous CHMI's(25), piperaquine was administered at single de-escalating dosages. The lowest dose used (480 mg) resulted in complete clearance of parasitemia in 3 out of 6 subjects (cohort 3B) within 30 to 36 hours after piperaquine treatment. Recrudescent parasitemia occurred in 3/6 volunteers which was completely cleared after a second dose of piperaquine (960 mg). Recrudescent infection was identified by routine 18S qPCR monitoring. In CHMI-trans1, 6/8 volunteers receiving low dose piperaquine (480mg) had a rapid recrudescent infection within 48 hours. Therefore, a second regimen of low dose piperaquine (T2, 480mg) will be given to volunteers in this study if a recrudescent infection occurs after T1 and prior day 21. The addition of the early initiation of a second subcurative treatment with piperaquine will increase the asexual parasite biomass without increasing the peak asexual densities to ensure the safety of the participants. The curative dosing regimen (T3) and in accordance with group assignment, the higher dose (960 mg) will be given to clear recrudescent parasitemia, without affecting (mature) gametocytes.
The rationale to use SP is based on field studies, showing an increase of transmissibility to mosquitoes after a curative treatment regimen. Furthermore, CHMI-trans1 has shown that gametocytes are not killed when a curative dose of SP is used (1000mg/50mg). In addition, the gametocyte densities and the area under the curve of gametocytemia were highest in the LD-PIP/SP arm, therefore the additional arm with SP as curative regimen is added to this study.

6.5. Potential Risks

See Summary of Product Characteristics (SPC) for Fansidar®, the Investigator's Brochure for piperaquine, and section 6.3 Summary of findings from clinical studies above.
Based on preliminary data(25) and clinical data accumulated during previous malaria challenge studies, piperaquine was well tolerated in the treated participants. They showed a robust safety profile in the treated participants in dose of up to 960 mg when used for the treatment of uncomplicated *P. falciparum* malaria infection.
Treatment of uncomplicated malaria with sulfadoxine-pyrimethamine has been widely implemented in the last decade and has been one of the most widely used antimalarial treatments in the world with millions of doses administered. Clinical data show a strong safety profile of SP and treatment is generally very well tolerated(39, 42, 60, 61).

The risk of treating with subcurative regimens for both drugs is limited to a recrudescent infection. This risk is mitigated by the extensive follow-up (twice a day) until 4 days after T1, and once a day after that. In case recrudescence occurs, volunteers will be treated with a second subcurative regimen (T2) if a recrudescent infection occurs prior day 21. On day 21 or recrudescence after T2, volunteers will be treated with a curative regimen of sulfadoxine-pyrimethamine or piperaquine, in accordance with group assignment. Furthermore, in the unlikely case a second recrudescence occurs after T3, final treatment with Malarone® will be initiated.

6.6. Potential Benefits

There are no expected benefits of sulfadoxine-pyrimethamine or piperaquine for participants participating in this study.

6.7. Risk Management

Potential risks have been identified through review of previous clinical studies conducted to date as well as review of the literature and post-marketing data for both sulfadoxine-pyrimethamine and piperaquine. Monitoring of cardiovascular effects after piperaquine treatment will be performed using a 12-lead ECG with a focus on expected maximal piperaquine concentrations after oral dosing (within 4h-12h after treatment). Healthy volunteers with a history of cardiovascular disease or clinically significant ECG abnormalities will be excluded from participation in the study, with particular attention paid to cardiac conduction. The risk to participants in this trial will be minimized in two ways:

1. Adherence to the inclusion/exclusion criteria.
2. Close clinical and laboratory monitoring to ensure the safety and wellbeing of the healthy participants.
3. Volunteers with increases in QT/QTc to >480 ms or >30 ms over baseline, after piperaquine treatment (T1 or T2) will not receive further dosing of piperaquine during the trial. These volunteers will instead receive the alternative drug, sulfadoxine-pyrimethamine as T3.

The overall risk to participants participating in the study is considered to be minimal and acceptable, and the potential of the results of this study to contribute towards future accelerated product development of malaria transmission blocking interventions is considered to outweigh these potential risks.
Scheduled regular clinical chemistry and haematology blood tests will also be performed, (details of time points can be seen in section 9.3.17).

6.8. Preparation and labeling of Investigational Medicinal Product

Piperaquine: Piperaquine tablets (160mg tablet strength as piperaquine phosphate) will be supplied to Radboudumc as HDPE bottles supplies which are manufactured and tested for quality control purposes in accordance with Good Manufacturing Practices by Penn Pharmaceutical Services Limited in the United Kingdom. The piperaquine tablets (160mg tablet strength as piperaquine phosphate) are packaged in HDPE bottles. The tablet supplies will be labelled in accordance with EMA GMP requirements and the label will include information regarding identity, batch number, expiry date and storage condition.

Piperaquine tablet supplies will be held at the nominated storage condition of 15°C-25°C and protected from moisture in appropriate locked storage conditions at Radboudumc until required.

Sulfadoxine-pyrimethamine, (and ***Malarone***®*)* will be acquired by Radboudumc, labelled according to identity, brand or source, and batch number. The supplies will be held in appropriate locked storage conditions at Radboudumc until required. The contents of the label for drug to be administered to the participants will be in accordance with all applicable regulatory requirements.

6.9. Drug accountability

The principal investigator must ensure that sulfadoxine/pyrimethamine and piperaquine are stored in an appropriate storage room. Accurate records must be maintained regarding the receipt of the treatments, which include: drug name, date received, lot number, amount received.
Accurate records must also be maintained regarding administration of drugs to volunteers. These records will be kept by the investigators. This includes:

- Volunteer identification number
- Date and dose of drugs dispensed
- Signature of the person administrating the drugs.

7. NON-INVESTIGATIONAL PRODUCT (SPOROZOITE CHALLENGE PRODUCT)

7.1. Name and description of non-investigational product(s)

P. falciparum 3D7-infected *Anopheles stephensi* mosquitoes for the purpose controlled human malaria infection.

7.2. Summary of findings from non-clinical studies

The *P. falciparum* 3D7 parasite is a clone of the NF54 strain which was isolated from a patient living in the Schiphol-area in the Netherlands. In clinical studies and *in vitro* studies, this parasite has been shown to be completely susceptible to multiple antimalarials, including sulfadoxine-pyrimethamine, piperaquine, chloroquine, mefloquine, atovaquone/proguanil and arthemeter/ lumefantrine (see D2c, 'Product information *Plasmodium falciparum* infected *Anopheles* mosquitoes').

7.3. Summary of findings from clinical studies

CHMIs are well accepted as a powerful tool for the evaluation of parasite development in humans. The Radboudumc has the experience and infrastructure to conduct CHMIs. Our center uses a sensitive method for parasite detection by real-time qPCR that allows detection of malaria infection in an early stage, resulting in early treatment.

There is a large clinical experience with infecting humans by the bite of *P. falciparum*-infected mosquitoes or by blood stage inoculum. Since 1986 more than 3,500 volunteers at global scale have had a CHMI by the bites of mosquitoes fed on cultures of *P. falciparum* gametocytes to produce *P. falciparum* sporozoites or by inoculation of infected-erythrocytes (Chulay et al., 1986, (67, 68)). This has proven to be a reproducible, predictable and safe method of inducing *P. falciparum* malaria. The results of such studies were summarized in 1997 (Church et al., 1997), in 2007 (Epstein et al., 2007) and in 2012 (Roestenberg, de Vlas, Nieman, Sauerwein, & Hermsen, 2012) and ((69)). Most CHMI studies worldwide are conducted with the 3D7 clone.

The *P. falciparum* isolate NF54 and the 3D7 clone have been tested for the purpose of challenge infection in 376 volunteers in 23 CHMI studies in the Radboudumc. In these studies >98 % of malaria-naive volunteers developed patent parasitemia after bites from 5 NF54 infected mosquitoes or blood stage inoculum. The time range to blood stage parasitemia detectable by thick smear (prepatent period) was between 7.0 and 14 days (after sporozoite challenge). Mild-moderate solicited adverse events were generally experienced by all study subjects, most commonly headache (95%), general malaise/fatigue (65%) and fever (90%)(30). Gastro-intestinal complaints, including nausea, diarrhoea and abdominal pain were also reported, mainly following intake of atovaquone-proguanil. Symptoms that were severe enough to prohibit daily activities, occurred in 49% of participants.

7.4. Summary of known and potential risks and benefits

There is no benefit expected for subjects participating in this study. The risk to subjects after challenge infection with 3D7-infected mosquitoes will be minimized by adherence to the inclusion/exclusion criteria and close clinical monitoring, which ensures that subjects with malaria will be treated at earliest stages of parasitemia. The risks of a CHMI for malaria-naïve subjects include i) discomfort induced by mosquito bites, ii) discomfort associated with periodic blood drawing and iii) risk of acquiring mild clinical *P. falciparum* malaria.

Mosquito bites are known to cause mild discomfort associated with mosquito feeding. A small degree of inflammation and pruritus typically accompanies the bite of the insect. Anaphylaxis after mosquito bites is extremely rare and has never been reported after CHMI. While significant allergic

reactions are extremely rare, in the event of an allergic reaction, epinephrine, anti-histamines, on-call physician and resuscitation equipment are available on site. The Radboudumc, an established site for CHMIs, is fully equipped to manage anaphylaxis and any other medical emergency.
Frequent blood draws will be necessary to closely monitor the subjects and to perform qPCR and thick smears for detection of *P. falciparum* parasitemia. Gametocyte densities are strongly associated with the preceding densities of the asexual progenitors. Therefore, in this study the asexual parasite burden will be monitored and allowed to reach levels where appropriate gametocyte production is expected without compromising the safety of the subjects. Subjects in this study will be treated at a parasite density threshold of 5000 parasites/mL or positive thick smear (sporozoite challenge), or on day 8 (blood stage challenge) which has shown to be safe. Recent CHMI studies have used lower treatment thresholds by qPCR detection (100 parasites/mL), however, the parasite burden in those subjects appeared to be too low for sufficient gametocyte production. Consequently, the higher parasite thresholds have been defined for treatment to meet these objectives, but still within the range of acceptability for safety according to experience at QIMR Berghofer studies. Universal precautions will be taken for protection of the volunteer and study personnel. The total amount of blood collected will be maximally 500 ml over the trial period (of 51 days, plus up to 120 days for the screening period) in agreement with guidelines of the Sanquin blood bank.
Intensive follow-up with qPCR performed on samples taken twice daily (and thick smears once daily in the evening), will ensure early treatment - preventing high levels or prolonged duration of parasitemia that would put the subject at undue risk. Severe/complicated malaria has never been described in a CHMI. Mild malaria symptoms include headache, myalgia, fever, chills, sweats, nausea, vomiting, and diarrhoea. Researchers at the Radboudumc have extensive experience with the care of clinical malaria.
It is expected that volunteers subjected to a treatment threshold of 5000 parasites/mL or positive thick smear, in contrast to a lower threshold such as 100 parasites/mL, will experience more symptoms and have a higher occurrence of transient, asymptomatic liver function derangements related to the mild malaria after CHMI (see also section 1.4 Safety). No relation has been found between parasite densities and the three cardiac events reported in CHMI (section 1.4 Safety). Nevertheless, rapid diagnosis by qPCR and/or thick smears and subsequent treatment at these parasite densities quickly attenuates the illness so that the infection does not place the subject at undue risk.
The exposure to infected *P. falciparum* 3D7-infected mosquito bites will occur at Radboudumc insectary which has a double-door barrier system along with a double blower (negative pressure wind blockade).

7.5. Description and justification of route of administration and dosage

Study subjects will be exposed to malaria for the purpose of challenge infection through (cutaneous) bites of *P. falciparum* 3D7-infected female *Anopheles stephensi* mosquitoes. This is the natural route of infection and the one with which most experience has been accumulated in CHMI trials. Volunteers will be exposed to the bites of 5 3D7-infected mosquitoes, which is the gold-standard dosage for CHMI studies worldwide.

7.6. Dosages, dosage modifications and method of administration

See also paragraph 7.5. Subjects receiving a malaria challenge infection with the 3D7 clone of *P. falciparum* will receive 5 infectious bites from *A. stephensi* mosquitoes infected with 3D7

sporozoites. Previous studies have demonstrated that with this dosage, near 100% of malaria-naïve volunteers develop a blood stage malaria infection (Verhage et al., 2005).

7.7. Preparation and labeling of Non Investigational Medicinal Product

The culture of parasites and infection of mosquitoes has been a routine procedure for over 20 years in the Malaria Unit of the Central Animal Facility of the Radboudumc, Nijmegen. The 3D7 parasite is a clone of the NF54 *P. falciparum* isolate, originated from the Schiphol area. The isolate was originally derived from patient material and is cultured in vitro in RPMI-1640 medium with 10% serum and 5% haematocrit red blood cells. Both the serum and the red blood cells are obtained from the Nijmegen department of the Sanquin Bloedbank region Zuid-Oost. These blood products are negative for malaria and Hepatitis B surface Anitgen (HBsAg), and seronegative for HIV, HCV, HumanT-lymphotropic Virus (HTLV) 1+2 and syphilis. The cultures are checked for bacterial, fungal and *Mycoplasma* contamination.

After 14 days of *in vitro* culture, the sexual stage parasites will be harvested for feeding to 1-5 days old laboratory cultured *A. stephensi* mosquitoes via a 'membrane feeder'. The percentage *P. falciparum*-infected mosquitoes will be assessed 6-9 days after feeding and one day prior to the CHMI. Mosquitoes will be kept in the same midi-cage from Membrane Feed for Sporozoite production until the day before CHMI. On the day before CHMI, a sample of 10 mosquitoes from each batch is checked for the presence of sporozoites. The best batch will be selected for CHMI requiring a batch of at least 40% infected mosquitoes. Mosquitoes are then transferred from midi-cages to small CHMI-cages. Each step in this process is performed by an experienced technician, and checked and recorded on standardized forms by another technician according to standard operating procedures.

7.8. Drug accountability

The date, time of collection and person collecting the mosquitoes is filled in on a standard table. This section will be signed by the responsible employee.

8. NON-INVESTIGATIONAL PRODUCT (BLOOD STAGE CHALLENGE PRODUCT)

8.1. Name and description of non-investigational product(s)

P. falciparum 3D7-infected human erythrocytes administered intravenously for the purpose controlled human malaria infection.

8.2. Summary of findings from non-clinical studies

Please refer to D2g Investigator's Brochure. The inoculum containing *Plasmodium falciparum* strain 3D7 has been derived from blood donated from a donor with clinical manifestation of malaria. The preparation of this challenge inoculum has been described in detail ((67, 68)). Briefly, the cells were purified from a donor previously infected with *P. falciparum,* strain 3D7 via mosquito bites. Before the infection, the donor was extensively screened and no serologic evidences were found for the screened infectious agents with exception of seropositivity for Epstein-Barr virus and cytomegalovirus. However, the stored blood sample is PCR negative for both viruses, indicating absence of viral DNA. Once the donor was microscopically positive for presence of malaria parasites, one unit of blood (500 ml) was collected from the donor and processed to remove leucocytes. The packed blood cells were then mixed with glycerolyte 57 solution (Baxter, Deerfield, IL) and cryopreserved in ~1 mL aliquots as previously described ((67, 68)) and stored at QIMR Berghofer under controlled conditions. In clinical studies and *in vitro* studies, this parasite has been shown completely susceptible to multiple antimalarials, including sulfadoxine-pyrimethamine, piperaquine, chloroquine, mefloquine, atovaquone/proguanil and arthemeter/ lumefantrine (see D2g, Investigor's Brochure *Plasmodium falciparum* 3D7-infected blood stage inoculum).

8.3. Summary of findings from clinical studies

Please refer to D2g Investigator's Brochure and section 7.4 above. The *Plasmodium falciparum* 3D7 parasite bank for blood stage challenge has been used to inoculate 296 malaria naïve study participants in previous challenge studies. No SAEs related to the challenge inoculum have been reported in any of these studies ((69) and McCarthy et al., unpublished).

The time range to blood stage parasitemia detectable by PCR (prepatent period) is reliably between 3 and 4 days. Mild-moderate solicited adverse events are generally experienced by all study subjects, most commonly headache, general malaise/fatigue and fever (30). Gastro-intestinal complaints, including nausea, diarrhoea and abdominal pain have also been reported, mainly following intake of piperaquine or atovaquone-proguanil.

8.4. Summary of known and potential risks and benefits

Please refer to D2g Investigator's Brochure. There is no benefit expected for subjects participating in this study. The risk to subjects after challenge infection with 3D7-infected human erythrocytes will be minimized by adherence to the inclusion/exclusion criteria and close clinical monitoring, which ensures that subjects with malaria will be treated at earliest stages of parasitemia. The risk of developing Red Blood Cell (RBC) alloantibodies in this study is considered extremely low since the donor blood used to produce the inocula was blood group O Rh(D) Negative. People with this blood group are generally considered "universal donors", as recipients of their blood have minimal risk of developing RBC alloantibodies when given much larger volumes of blood than is used in the blood stage challenge model. However, it is possible that subjects could suffer a transfusion reaction after they receive the inoculum, or develop alloantibodies to the donor RBCs that may make blood

transfusion more difficult in the future. To date, one subject has developed an antibody response to a minor Rh antigen (anti-E antibody) following blood stage inoculation with P. falciparum 3D7 (see Investigator Brochure). However, there was no laboratory evidence to indicate that the specific Rh phenotype of the donor RBCs in the inoculum provoked production of this allo anti-E antibody. Volunteers will be monitored for signs and symptoms in the period immediately after administration of the inoculum to further assess the risk of the inoculum in causing transfusion reaction. Volunteers of cohort B will also be tested for RBC alloantibodies at screening and at the end of the study as part of their safety monitoring (section 9.3.17).
Women of childbearing potential (WOCBP) have a small additional risk of developing RBC alloantibodies that could cause problems during pregnancy. WOCBP have participated in several blood stage malaria trials with *P. falciparum* isolate 3D7 with no known issues to date. WOCBP will receive matched donor blood, i.e. Rh(c) and/or Rh(e) negative woman will be excluded to participate in group 3 or 4, since the donor blood is Group O (Rh)D Negative ; C negative; E negative ; c positive ; e positive i.e. **rr** and Kell negative. Furthermore, participants with a history of blood transfusion are excluded to participate in these groups. Specific strict contraception requirements will be requested for this population during the study (inclusion/exclusion criteria). Including WOCBP in the trial enhances the generalisability of the study results. Please also see the investigor's brochure and section 7.4 above for the summary of known and potential risks and benefits after the malaria challenge in this study. The exposure to the blood stage malaria inoculum will occur at Radboudumc.

8.5. Description of route of administration and dosage

Please refer to D2g Investigator's Brochure. The inoculation will take place at the Clinical Research Centre Nijmegen (CRCN) in the Radboudumc. The inoculum will be administered by intravenous injection into an indwelling intravenous cannula. Approximately ~2,800 infected red blood cells will be injected in a total volume of 2 mL of normal saline followed by a saline flush. The cannula will then be removed, and hemostasis ensured by use of an appropriate dressing. The time between preparation of the final inoculum and inoculation will be maximum 4 hours, during which time all inocula will be stored on ice. All volunteers will receive the inoculum within 60 minutes after preparation. Subjects will be observed closely for one hour before discharge. Emergency aid kits will be present and readily available at any location, whenever there are volunteers present.

8.6. Preparation and labeling of Non Investigational Medicinal Product

Please refer to D2g Investigator's Brochure. The culture of parasites has been a routine procedure for over 20 years in the Malaria Unit of the Central Animal Facility of the Radboudumc, Nijmegen. The 3D7 parasite is a clone of the NF54 *P. falciparum* isolate, originated from the Schiphol area. The isolate was originally derived from patient material and was frozen directly after processing. These blood products are PCR negative for blood-borne virses, including Hepatitis B, HIV, HCV, HumanT-lymphotropic Virus (HTLV) 1+2 and seronegative for syphilis. The bank has tested negative by culture for bacteria and fungi and for *Mycoplasma* contamination.
The infective inoculum will have been prepared from a single aliquot of the cryopreserved infected packed blood cells aliquots prepared as previously described (68) (Please refer to D2g Investigator's Brochure for detailed description). Each dose of 2 mL will contain ~2,800 viable parasite infected erythrocytes. The inoculum will be prepared aseptically, as outlined in Standard Operating Procedures documents. The actual number of parasites inoculated will take into account the loss of viability resulting from cryopreservation, storage, and thawing. Previous experience indicates that

parasite viability following this process is ~30%, thus requiring ~8,300 infected erythrocytes per inoculum. The parasitemia of the cell bank was 212 p/µL blood which is nearly double in the frozen packed blood cells. Thus, each inoculation will be prepared to contain 250 µL of the thawed and diluted packed blood cell sample. This blood sample will contain about 19.5×10^7 erythrocytes and ~8,300 infected erythrocytes of which around 2,800 of the parasites will be viable.
Thawing and washing of the inoculum will be done with commercial cGCP solutions for human use and with disposable syringes and needles according to standard operating procedures used in 24 previous studies at the Queensland Institute of Medical Research and Oxford University. Sample manipulations will be performed within a safety cabinet that has been especially cleaned and set aside for this purpose. After thawing of the blood inoculum, the cold chain (2-8 degrees) will be maintained at all times until the inoculum has been administered to the volunteer. Each step in this process is performed by an experienced technician, and checked and recorded on standardized forms by another technician according to standard operating procedures.

8.7. Drug accountability

The syringes containing the inocula of the blood stage parasites will be prepared at Radboudumc on the challenge day. The responsible employee will document receipt conditions and time restrictions of use.

9. METHODS

9.1. Study parameters/endpoints

9.1.1. Main study parameter/endpoint

- Frequency and magnitude of adverse events in the CHMI-trans model.
- Prevalence of gametocytes and gametocyte infectiousness for *Anopheles* mosquitoes through mosquito membrane feeding assay (Direct Membrane Feeding Assay, DMFA).

9.1.2. Secondary study parameters/endpoints

- Peak density and time-point of peak density of gametocytes by qRT-PCR.
- The area under the curve of gametocyte density versus time.
- Assessment of the dynamics of gametocyte commitment, maturation and sex-ratio.
- Prevalence of gametocyte infectiousness for *Anopheles* mosquitoes through Direct Feeding Assays (Direct Skin Feeding Assay, DFA).

9.1.3. Exploratory study parameters/endpoints

- Quantification of gamete formation and fertilization as ex vivo indicators of gametocyte fitness.
- Antibodies to proteins expressed/exported during early gametocyte development.
- The association between the timing and density of gametocyte carriage and antibody responses to gametocyte antigens (including but not restricted to Pfs48/45, Pfs230, Pfs16, PfGEXP5).
- Antigen specificity and/or functionality of CHMI induced antibodies against *Plasmodium falciparum.*
- The specificity and/or functionality of CHMI induced T cell responses against *Plasmodium falciparum.*
- Epigenetic profiling of immune cells via Chromatin Immunoprecipitation and sequencing and/or RNA transcriptome profiling through whole mRNA-sequencing, PCR or microarray.
- Phenotype and/or function of immune responses to CHMI (with specific focus on innate lymphoid cells, ɣδT cells, monocytes, platelets, NK cells and granulocytes).
- Comparative transcriptome analysis of 3D7 blood stage challenge parasites and sporozoite challenge parasites during CHMI-trans.
- Hematological profiles for diagnostics in malaria
- To explore associations between elevated transaminases and inflammatory biomarkers (CRP, Ferritine, IFNɣ, IL-6, IL-10, TNF-r2, TGF-β, and MDA).

9.2. Randomisation, blinding and treatment allocation

This is an open-label study. The 24 volunteers will be allocated to one of the two groups per cohort at random according to a randomization list derived from the Microsoft Excel command ASELECTUSSEN (0,1000). Stratification will not be performed. An independent investigator at the Radboudumc, not involved in the clinical trial, is responsible for performing the randomization and for assigning volunteers according to the randomization list. A second employee, not involved in the assignment of volunteers, will check to see if randomization is done correctly. Every volunteer number per cohort will be linked to a random number between 0 and 1000. The six lowest numbers per cohort will be assigned group 1 (for cohort A) or 3 (for cohort B), the six highest numbers per cohort will be assigned to group 2 (for cohort A) or 4 (for cohort B). If two identical numbers are produced, the

whole procedure is repeated. The randomization list is kept in a fireproof clinical trial cabinet at Radboudumc.

9.3. Study procedures

9.3.1. Screening period: Screening, Inclusion and Baseline visits

Volunteers who wish to participate in the trial will be asked to complete an informed consent questionnaire. Their understanding of the trial will be tested after discussing the study with the investigator during informed consent, and prior to being asked to sign and date the consent form. Volunteers who fail to answer all questions correctly on their first attempt are allowed to re-take the questionnaire following further discussion with the investigator, and provided they subsequently answer all questions correctly, they may then complete the consent form and be screened for the trial.

Subjects who sign informed consent will undergo complete screening including a medical history, physical examination, vital signs, ECG, urine tests and laboratory evaluations (see sections 9.3.10). If physical examination, vital signs or laboratory values are out of the normal range a repeat measurement may be obtained.

Subjects who meet the eligibility criteria will be invited back for enrollment into the study at the inclusion visit, which will occur within 3 to 8 days prior to the planned challenge day (day 0). Some screening assessments, including physical examination, vital signs, urine tests and laboratory evaluations, will be repeated at this inclusion visit.

Following this screening period of up to 120 days, subjects who continue to meet the eligibility criteria (Section 4.2 and 4.3) will present to the study site the day before the challenge infection for baseline assessments. Patient history will be taken and all adverse events that have occurred since screening will be noted. Only subjects who still meet the inclusion criteria will receive bites of infected mosquito bites or be inoculated with parasitized erythrocytes. For each subject, study start will be defined as the day of the inclusion visit.

9.3.2. Controlled Human Malaria Infection

Only subjects that met the inclusion criteria will undergo a malaria challenge infection.

Cohort A, on the challenge day all subjects will be exposed to the bites of five 3D7 *P. falciparum* infected mosquitoes. Mosquito feeding will be allowed for 10 minutes. Volunteers will receive a local treatment (tripelennamine crème) for mosquito bites and will be observed for 15 minutes after the feed. Directly after the feed, the mosquitoes will be dissected by a technician of the mosquito unit according to standard operating procedures. Exposure will be repeated until five infected mosquitoes have fed on each volunteer.

As long as there are volunteers present in the mosquito unit, there will be supervision by one of the clinical investigators. Another clinical investigator will be on call, in case of emergency. Emergency aid kits will be present and readily available at any location, whenever there are volunteers present.

Cohort B, on the challenge day all subjects will receive a blood stage inoculum containing 3D7 *P. falciparum* parasites. Subjects will be cannulated with an indwelling intravenous cannula for the malaria inoculum, and recorded which arm is utilized. The malaria inoculation will take place in the CRCN of the Radboudumc. As long as there are volunteers present in the CRCN, there will be supervision by one of the clinical investigators. Another clinical investigator will be on call, in case of emergency. Emergency aid kits will be present and readily available at any location, whenever there

are volunteers present. Volunteers will be observed for a minimum of 60 minutes after administration of the inoculum to evaluate for immediate adverse reactions.

After malaria challenge infection all subjects will be observed closely according to an intensive out-patient follow-up schedule including frequent safety analyses (see section 9.3.17 for details). The study design is illustrated in more detail in the flowchart in section 9.3.17. Subjects are required to reside locally within close proximity to the trial centre (<10 km) or (if >10km) be willing to stay in a hotel close to the trial centre during part of the study (from day 4 (blood stage challenge) or day 5 (sporozoite challenge) post-infection until T1+4 provided that the subject has had 2 consecutive negative 18S qPCR tests (at least 24 hours apart) following T1 or T2 treatment; or until day T3+3).
For all subjects, during this period all relevant investigations will be carried out on an outpatient basis at Radboudumc, including frequent safety analyses (section 9.3.17 for details).
From day 4 (blood stage challenge) or 6 (sporozoite challenge) up until four days after treatment 1 (T1+4) post-CHMI, assessments of parasite densities using qPCR will be performed on samples collected twice daily. Read-out of qPCR will only take place once daily. Therefore, a thick blood smear analysis will be performed in the evenings from day 5 (blood stage challenge) or day 7 (sporozoite challenge) until day of treatment (T1). Once daily assessment by qPCR will start from 5 days post treatment 1 (T1+5) until three days after day of treatment 3 (T3+3). From T3+3 until day 36, subjects will be seen 3 times a week or otherwise in case of symptoms. Subjects will measure their temperature twice daily, and contact the clinical investigators when any symptoms, complaints or fever occurs. All subjects will receive a final treatment (ET) according to national guidelines with atovaquone/proguanil (Malarone®) on day 36. In case a volunteer remains 18S qPCR and Pfs25 qRT-PCR negative for 7 days after T1 or T2, final treatment with Malarone® will also be initiated and end of study will apply for the volunteer. All subjects will visit the study site for a follow-up visit on day 2 after commencing final treatment (ET+2). All subjects will be seen for a final control visit on day 51 after CHMI. The study design is illustrated in more detail in the flowchart in section 9.3.17.
Day time qPCR assessment will be performed in real time while evening samples will be assessed the next morning. To ensure safety, thick smears will be performed on all subjects from day 5 (blood stage challenge) or 7 (sporozoite challenge) until T1 who attend for follow-up in the evening or when a subject is febrile (≥38.0°C) after T1+4 (after morning visits). If a thick smear is positive in the evening (including symptoms for the blood stage challenge), treatment will be started immediately.
As soon as a qPCR or thick smear result is deemed positive for malaria parasites, the technician will inform the trial clinician.

Treatment 1 (**T1**) will be initiated using the following criteria:

1. One positive 18S qPCR above 5000 parasites/mL (blood stage challenge only: and is accompanied by a clinical symptom score ≥ 6 or on day 8)
2. One positive thick smear (which will be made if a volunteer attends an evening follow-up visit or on decision of the trial clinician) (blood stage challenge only: and is accompanied by a clinical symptom score ≥ 6).

Treatment 2 (**T2**) will be initiated using the following criteria:

1. Occurrence of recrudescent infection after T1 (prior day 21):
    - as assessed by 18S qPCR above 1500 asexual parasites/mL and (possibly or probably related) symptoms.

- (in absence of symptoms) as assessed by 18S qPCR & Pfs25 qRT-PCR above 1500 asexual parasites/mL.

2. One positive thick smear (which will be made if a subject is febrile (≥38.0°C) after morning visit) (blood stage challenge only: and is accompanied by a clinical symptom score ≥ 6).

Treatment 3 (**T3**) will be initiated using the following criteria:

1. Occurrence of recrudescent infection after T2 or on day 21:
   - as assessed by 18S qPCR above 1500 asexual parasites/mL and (possibly or probably related) symptoms.
   - (in absence of symptoms) as assessed by 18S qPCR & Pfs25 qRT-PCR above 1500 asexual parasites/mL.
2. On One positive thick smear (which will be made if a subject is febrile (≥38.0°C) after morning visit) (blood stage challenge only: and is accompanied by a clinical symptom score ≥ 6).

If treatment has to be initiated based on the above criteria, the trial clinician will contact the volunteer for immediate treatment. Preferably, the volunteer should return immediately or at least within 1 hour. If the volunteer is not reachable by phone, his/her contact person will be called and a search is started.

During the entire study period subjects will be instructed to call the trial physicians at any time if they experience severe symptoms. The trial clinician can decide to initiate additional diagnostics, clinical observation/monitoring or (symptomatic) treatment at all times.

For unexpected laboratory abnormalities, the laboratory test will be repeated. If there is any ambiguity regarding the decision to include or exclude a volunteer, the clinical supervisor will discuss the case with the local safety monitor and make the final decision after that, if necessary with consultation of a specialist. If volunteers prove to be eligible, they will be invited to the next visit.

All subjects who are exposed to *P. falciparum* will be treated. Treatment after challenge infection will be based on the above mentioned criteria. Additionally, final treatment with Malarone® (ET) can be initiated in any of the following situations:

1. By decision of study doctor or the safety monitor
2. In consultation with the cardiologist
3. On request of the volunteer
4. On day 36 post CHMI
5. When LDH > 1000 U/l

Additionally, treatment with T1 or T3 can be initiated in the following situation: when platelet levels are <120x 10^9/L T1 can be initiated, and when platelet levels are <50x 10^9/L T3 can be initiated. Furthermore, subcurative treatment will be initiated when ALT/AST levels are >2.5xULN (prior T1), and/or curative anti-malarial treatment will be initiated when ALT/AST levels are >5x ULN (prior T1).

9.3.3. Treatment with piperaquine (T1, T2, and T3 for group 1, 2, 3, and 4)

All volunteers will be treated with a subcurative regimen (T1) of LD-PIP (piperaquine 480mg). A second subcurative regimen (T2) of LD-PIP2 (piperaquine 480mg) will subsequently be initiated when recrudescence occurs prior day 21 post challenge infection. In group 1 and/or 3, a curative regimen (T3) of PIP (piperaquine 960mg) will subsequently be initiated when recrudescence occurs after T2 or

on day 21 post challenge infection. All volunteers will be treated based on the predetermined criteria mentioned above.

Treatment with piperaquine will be given after a fasting period of ≥ 3 hours. If dosing is to occur in the evening, subjects will be required to fast for ≥3 hours prior to receiving treatment. Subjects will be required to fast for a further four hours anytime after dosing with piperaquine.

During treatment, complaints of malaria infection will be treated symptomatically. In addition to specific treatment with piperaquine, symptomatic treatment will be administered at the discretion of the attending physician.
Volunteers will not be admitted to the hospital during this study, unless the study doctors or the safety monitor deems it necessary, or on request of the volunteer.

9.3.4. Treatment with sulfadoxine-pyrimethamine (T3 for groups 2 and 4)

All volunteers in group 2 and 4 will be treated with a curative regimen (T3) of SP (1000mg/50mg) when recrudescence occurs after T2 or on day 21, whichever occurs first. All volunteers will be treated based on the predetermined criteria mentioned above. During treatment, complaints of malaria infection will be treated symptomatically. In addition to specific treatment with SP, symptomatic treatment will be administered at the discretion of the attending physician.
Volunteers will not be admitted to the hospital during this study, unless the study doctors or the safety monitor deems it necessary, or on request of the volunteer.

9.3.5. Treatment with Malarone® (ET)

All volunteers will be treated with Malarone® (ET) on day 36. The treatment will consist of the drug Malarone® (atovaquon/proguanil). Dosing of Malarone® will be as follows: once daily 4 tablets of 250/100mg, during three days according to Dutch SWAB guidelines. During the first day and last day of Malarone® treatment qPCR is performed directly in collected blood samples. If qPCR remains positive after Malarone® treatment (usually the result of parasite debris remaining in the bloodstream) a thick blood smear will be performed to confirm the absence of intact malaria parasites.
Volunteers will not be admitted to the hospital during this study, unless the study doctors or the safety monitor deems it necessary, or on request of the volunteer.

9.3.6. Physical examination

A complete physical examination will include the examination of general appearance, skin, neck, eyes, throat, lungs, heart, abdomen, back and extremities, and a routine vascular and neurological examination.
Height (cm) and body weight (to the nearest 0.1 kilogram [kg] in indoor clothing, but without shoes) will also be measured, at screening only. Body mass index (BMI) will be calculated using the following formula: BMI = Body weight (kg) / [Height (m)]^2 and converted to an integer.

9.3.7. Vital signs

Vital signs including body temperature, blood pressure (BP) and pulse measurements will be determined and recorded at set time points during the study. Systolic and diastolic BP will be measured while the subject is sitting, with back supported and both feet placed on the floor, using an

automated validated device, with an appropriately sized cuff. In case the cuff sizes available are not large enough for the subject's arm circumference, a sphygmomanometer with an appropriately sized cuff may be used.
If vital signs are out-of-range at screening or inclusion, the investigator may obtain two additional readings, so that a total of up to three consecutive assessments are made, with the subject seated quietly for approximately five minutes preceding each repeat assessment. At least the last reading must be within the normal range in order for the subject to qualify.
Temperature will be measured according to local practice, consistently throughout the study. The thermometer used should have a precision of 0.1ºC. The same route should be used throughout the study.

9.3.8. Patient-reported outcomes (study diary)

At the inclusion visit, subjects will be issued symptom diaries and thermometers. They will be asked to record all symptoms and medication use daily from the day of inclusion until end of study. The subject diary will be reviewed at each study visit and used as a basis for discussion of possible adverse events or medication use. If the occurrence of an adverse event or use of medication is confirmed by the study physician, it is recorded in the subject's study file (see also: section 10.3).
Subjects will also be asked to measure their temperature orally every morning from the day of the malaria challenge infection until the third day of atovaquone/proguanil treatment, and record this temperature in their study diaries.
At the end of the study, the diary will be collected and kept as source data with the subject's study file.

9.3.9. Electrocardiogram

A standard 12 lead ECG will be performed at screening and 4-12 hours after treatment with piperaquine (focus on expected maximal piperaquine concentrations after oral dosing). Additional ECG assessments may be performed at anytime throughout the study at the discretion of the investigator.
All assessments will occur after the subject has rested for approximately 10 minutes in the supine position. Calibration should be performed per the local site requirements. Each ECG tracing will be labeled with the subject number and date, and kept in the source documents at the study site. Interpretation of the tracing must be made by a qualified physician and documented in the Case Report Form (CRF). Minimally, the CRF will contain date and time of ECG, heart rate, PR interval, QRS duration and QT interval (corrected). Clinically significant abnormalities will also be recorded in the CRF and reported to the Safety Monitor.

9.3.10. Blood sampling and safety laboratory evaluations

During the study, blood samples will be drawn for screening, safety and research purposes. The blood sampling schedule in the flowchart (section 9.3.17) shows when blood will be drawn. Hemoglobin, hematocrit, red blood cell count, white blood cell count with differential (e.g. neutrophils, basophils, eosinophils, monocytes, lymphocytes) and platelet count will be measured at regular time points during the study.
Alkaline phosphatase, total bilirubin, creatinine, γGT, LDH, potassium, AST, ALT, sodium, highly sensitive troponin T, urea, and amylase will be measured at regular time points during the study. Glucose, triglycerides and cholesterol will be measured only at screening. Volunteers will be tested for RBC alloantibodies (Rh(CDE) Kell typing, and antibody screening) at screening and at the end of the study.

In the event that an asymptomatic individual has evidence of an elevated troponin level, a second sample may be obtained to discern whether the result could represent a false positive (0.4% of tests).

A midstream urine sample (approx. 30 ml) will be obtained at screening, inclusion and, for female subjects only, at baseline. In this sample the presence of amphetamines and cocaine will be assessed; the sample taken at inclusion will also be tested for the presence of cannabis. Additionally, for female participants a commercially available hCG urine test will be used to test for pregnancy at screening and baseline.

In the case where a laboratory assessment is outside the reference range for the laboratory at screening and/or inclusion, a decision regarding whether the result is of clinical significance or not shall be made by the clinical investigator and shall be based, in part, upon the nature and degree of the observed abnormality. The assessment may be repeated once prior to randomization.

In all cases, the investigator must document in the source documents, the clinical considerations (i.e., result was/was not clinically significant and/or medically relevant) in allowing or disallowing the subject to continue in the study.

9.3.11. Analysis of asexual parasite densities after challenge infection

qPCR for assessment of parasite densities will be performed directly in volunteer samples, as discussed previously (see section 9.3.2).

qPCR is performed according to a standard procedure as previously described (Hermsen et al., 2001) with small adjustments. In short, qPCR will be performed on the multicopy 18S ribosomal RNA gene. All samples are spiked with the extraction control Phocine Herpes Virus (PhHV) to determine efficacy of DNA isolation.

Thick smears will be performed directly in volunteer samples, as discussed previously and if deemed necessary by the clinical investigator (see section 9.3.2), according to a standard operating procedure which is based on an internationally harmonized protocol for thick smears in CHMIs (Moorthy et al., 1998) and (WHO, 2010). Per slide, the number of fields correlating to 0.5 µl of blood will be read. Slides are considered positive if they contain 2 or more parasites in these fields.

9.3.12. Direct Membrane Feeding Assays (DMFA) and Direct Skin Feeding Assays (DFA)

A reproducible *CHMI-trans* model requires sufficient densities of circulating mature gametocytes in subjects to ensure the generation of adequate numbers of infected *Anopheles* mosquitoes after blood feeding, either directly on the skin (DFA) or through a membrane-covered device that contains a venous blood sample with gametocytes (DMFA).

Following treatment with sulfadoxine-pyrimethamine and/or piperaquine, transmission studies will be undertaken when gametocytemia appears. To evaluate infectivity in vector mosquitoes we will use a direct membrane feeding assay (DMFA) and a direct skin feeding assay (DFA). This study will use approximately 300 female mosquitoes for DMFA, and 20-30 female mosquitoes per cup for the DFA, per time-point to maximize the precision of mosquito infection estimates at low infection rates. For the DMFA, blood will be collected (see flowchart, section 9.3.17) from each participant for membrane feeding assays with *An. stephensi* mosquitoes. For the DMFA, blood will be kept at 38°C (to prevent premature exflagellation) for up to 35 minutes until dispensed into membrane feeders. Female mosquitoes (3-6 days old) will be distributed into 1L plastic containers with gauze lids (~300 females/container/time point) and starved overnight prior to feeding on *P. falciparum*-infected blood samples. Mosquitoes will be allowed to feed on the blood through parafilm membranes attached to

water jacketed glass feeders attached to a 39°C water bath. Mosquitoes will be allowed to feed for up to 30 min in the dark. In the CHMI-trans1 related experiments, we have achieved 90% feeding rate with our *Anopheles stephensi* colony. Non-engorged mosquitoes will be identified and discarded. After blood feeding, mosquitoes will be maintained in environmental chambers set at 26°C and provided with 5% glucose as described(70).
For the DFA, 20-30 mosquitoes in one cup will be allowed to bite on alternating sides of the volar surface of the forearms to directly feed for approximately 15 minutes to enable mosquitoes to fully engorge. The experimental infection of mosquitoes by DMFA and DFA will be performed up to 3 times prior to curative anti-malarial treatment at the End of Study with Malarone®.

Six to nine days after blood feeding, mosquitoes will be dissected and examined for oocysts in midgut preparations. For permanent preparations, oocysts will be stained with 1% mercurochrome in H2O for 5 to 60 minutes then fixed in 1% glutaraldehyde or formaldehyde(71). Oocysts will be counted per mosquito dissected and recorded. Relationship between parasitemia, gametocytemia and mosquito infection (both oocyst prevalence and intensity) will be determined using generalized-linear mixed models(72). The number of mosquitoes dying prior to dissection will be recorded. For mosquitoes that cannot be examined by microscopy due to logistical reasons (e.g. if multiple DMFA/DFA experiments are conducted on the same day, the number of available mosquitoes may exceed the maximum number that can be dissected), infection status will be determined on day 10 following the blood meal by circumsporozoite ELISA, followed by PCR confirmation based on the 18S rRNA target(73). At this day, ELISA-based detection of malaria parasites has equal sensitivity compared to microscopy or PCR(73, 74).

9.3.13. Quantification of gametocytes, and gametocyte sex ratio.

A detailed quantification of gametocytes, gametocyte sex ratio, and gametocyte infectivity will be assessed by using molecular markers, and ex vivo assessments of gametocyte fitness after CHMI challenge infection. Quantification of gametocytes will be performed using a Pfs25 mRNA QT-NASBA, and quantitative reverse transcription (qRT)-PCR(32, 75) P, targeting PfGEXP5(76), PF14_0748 (young gametocytes)(77) PF14_0367 (mature gametocytes)(77) and P230p (male gametocytes)(32). Additional markers of gametocyte maturity and sex ratio will be incorporated as these become available. qRT-PCR determination of circulating gametocytes will be performed on every day after detection of parasitemia by 18S qPCR.
Blood samples will be taken from EDTA-vials taken for routine qPCR analysis (see flowchart, section 9.3.17). 100µL will be directly transferred into a cryovial containing 400µL of RNAprotect for qPCR stage composition analysis. Another 1mL will be enriched prior to analysis, through gametocyte separation by magnetic sorting. This approach takes advantage of the differential deposition of paramagnetic hemozoin by different parasite stages: asexual ring stages have very low hemozoin content, while later stages including all gametocyte stages have higher content; therefore this method allows for the enrichment of gametocytes using magnetic fractionation(78, 79). In both samples, gametocyte presence will be determined using an established multiplex qRT-PCR stage composition assay that targets gametocyte specific RNA and detects both immature gametocytes and mature gametocytes (77). Furthermore, thick smears will be analysed on DMFA time-points to look for gametocytes (see 9.3.17 Flowchart Study Design).

9.3.14. Effect of systemic inflammation on transient subclinical organ injury

Bloodsamples will be taken for plasma/serum analysis to explore associations between elevated transaminases and different inflammatory biomarkers which are known to be increased during a CHMI or natural acquired uncomplicated malaria such as C-Reactive Protein(19), Ferritine(80), IFN-γ(19, 81), IL-6(19), IL-10(19, 81), TNF-r2 and TGF-β(82). A relation between these biomarkers and subclinical organ injury in malaria has never been explored. Furthermore, Malondialdehyde (MDA) will be assessed as a sensitive marker for oxidative stress to explore the relation with subclinical organ injury (40, 83-86).

9.3.15. Immunological assays

Blood samples will be taken for isolation of peripheral blood mononuclear cells (PBMCs) and serum (see flowchart 9.3.17).

PBMCs and sera will be frozen and can then be used by the Radboudumc or its collaborators for exploratory immunological assays to further analyse the phenotype or functionality of the immune response during and after malaria infection.

In order to assess (antigen specific) T cell responses, the HLA-type of volunteers may be determined.

Antibody responses will also be measured to gametocyte antigens, including recombinant proteins Pfs48/45 and Pfs230 using standard ELISA methodologies(87).

9.3.16. Case report forms and data collection

All data collected by the investigators is registered in electronic case report forms. The investigator's notes are collected in subject study files and are considered source data. Since all subjects will be healthy, there is no medical file for the study subjects, with exception of the medical file in case of adverse events/reactions resulting in a medical consultation or hospitalization. In this case the medical file will also be considered as the source data. The diaries, produced by the study volunteers are also considered source data. They will be kept as source documents together with the investigator's notes.

9.3.17. Flowchart Study Design

COHORT A - Sporozoite challenge															
	**Screening**	**Inclusion**	**Baseline**	**CHMI**	**Phase 1**					**Phase 2**					**EOS**
Visit Number	V1	V2	V3	V4	V5-34					V35-44					V45
Trial timeline	-120 to -11	-8 to -3	-1	0	6-20	T1[9]	T1+1-4	T2[9]	T2+1-3	T3[9]	T3+1-3	27-35	36 (ET)	ET+2 (38)	51
Number of visits	1x	1x	1x	1x	2x/day	2x/day	2x/day			1x	1x	3x/week	1x	1x	1x
Informed consent	X														
Eligibility criteria	X		X												
Demographic data, Medical history	X														
Physical examination and vital signs	X	X			X[10]	X[10]	X[10]	X[10]	X[10]	X[10]	X[10]	X[10]	X[10]	X[10]	
ECG	X					X[11]		X[11]		X[11]					
Temperature	X	X	X		X	X	X	X	X	X	X	X	X	X	X
Challenge with 5 infected mosquitoes				X											
Collecting (serious) adverse events	as necessary[16]														
Subcurative treatment[1]						X		X							
Curative treatment[2]										X					
Final treatment (ET; Malarone®)													X		
Haematology tests[3] (2.0ml)	X	X			X	X	X	X	X	X	X	X	X	X	X
Biochemistry tests[4,18] (3.0ml)	X	X	X		X[12]	X[12]	X[12]	X[12]	X[12]	X[12]	X[12]	X[12]		X[12]	X[12]
HsTropT + LDH (2.0ml)					X	X	X	X	X	X	X	X	X	X	
Serology (5.0ml)[5]	X	X													
Pregnancy and toxicology urine test[15]	X	X	X												
Parasitology[6] (qPCR)			X		X	X	X	X	X	X	X	X	X	X	X
Parasitology (thick blood smear)					X[13]					X[13]		X[13]	X[13]		
Parasitology[7] (qRT-PCR)			X				X		X	X	X	X	X	X	X
DMFA and DFA										X[14]		X[14]	X[14]		
Exploratory immunology			X		X	X	X	X	X	X	X	X			X
Safety report[8]										X					X
Total blood volume collect[17]	12.5	8.5	61	0	223					134					61
[1] piperaquine (480mg)															

[2] piperaquine (960mg) or sulfadoxine-pyrimethamine (1000mg/50mg) according to treatment arm
[3] Hemoglobin, hematocrit, platelets, red blood cell count, white blood cell count + differentiation
[4] Creatinine, urea, sodium, potassium, bilirubin, AF, γGT, AST, ALT, LDH and hs-Trop-T. Additional at screening: cholesterol, triglyceride + glucose
[5] HIV, HBV, HCV, *P. falciparum(screening only)*
[6] 18S qPCR for blood stage *P. falciparum*. Additional thick blood smear only on indication, blood will be taken from qPCR samples.
[7] Pfs25 qRT-PCR for sexual stage *P. falciparum.*
[8] A safety report will be compiled upon all subjects having completed day 21 after challenge and at the end of the study.
[9] See treatment criteria section 9 Methods
[10] On indication. Performed at treatment visit only when volunteers are qPCR positive.
[11] Within 4-12 hours after treatment with piperaquine
[12] On C+6, C+8, C+10, T1, T1+2, T1+4, T1+6, T2, T2+2, T2+4, T2+6, T3, T3+2,T3+6, ET+2, and EOS
[13] On evening visits from day 7 until T1, and at DMFA timepoints. Additional thick blood smear only on indication, blood will be taken from qPCR samples.
[14] On day 21, 24 and between day 21-36 (based on the height of gametocytemia as measured by Pfs25 qRT-PCR)
[15] Toxicology urine tests at screening and inclusion visits only. Pregnancy test for female subjects at screening and baseline visits only,
[16] Only SAE's that are possibly or probably related to trial will be collected prior to inclusion.
[17] Total blood volume: 500ml
[18] Additional amylase will be measured on screening, baseline and T1+2

COHORT B – Blood stage challenge

NL63552.000.17 **CHMI-trans2 protocol**

	Screening	Inclusion	Baseline	CHMI	Phase 1					Phase 2					EOS
Visit Number	V1	V2	V3	V4	V5-30					V31-40					V41
Trial timeline	-120 to -11	-8 to -3	-1	0	4-7	T1[9]	T1+1-4	T2[9]	T2+1-3	T3[9]	T3+1-3	27-35	36 (ET)	ET+2 (38)	51
Number of visits	1x	1x	1x	1x	2x/day	2x/day	2x/day			1x	1x	3x/week	1x	1x	1x
Informed consent	X														
Eligibility criteria	X		X												
Demographic data, Medical history	X														
Physical examination and vital signs	X	X			X[10]	X[10]	X[10]	X[10]	X[10]	X[10]	X[10]	X[10]	X[10]	X[10]	
ECG	X					X[11]		X[11]		X[11]					
Temperature	X	X	X		X	X	X	X	X	X	X	X	X	X	X
Challenge with infected erythrocytes				X											
Collecting (serious) adverse events	as necessary[16]														
Subcurative treatment[1]						X		X							
Curative treatment[2]										X					
Final treatment (ET; Malarone®)													X		
Haematology tests[3,19] (2.0ml)	X	X			X	X	X	X	X	X	X	X	X	X	X
Biochemistry tests[4,18] (3.0ml)	X	X	X		X[12]	X[12]	X[12]	X[12]	X[12]	X[12]	X[12]	X[12]		X[12]	X[12]
HsTropT + LDH (2.0ml)					X	X	X	X	X	X	X	X	X	X	
Serology (5.0ml)[5]	X	X													
Pregnancy and toxicology urine test[15]	X	X	X												
Parasitology[6] (qPCR)			X		X	X	X	X	X	X	X	X	X	X	X
Parasitology (thick blood smear)					X[13]					X[13]		X[13]	X[13]		
Parasitology[7] (qRT-PCR)			X				X		X	X	X	X	X	X	X
DMFA and DFA										X[14]		X[14]	X[14]		
Exploratory immunology			X		X	X	X	X	X	X	X	X			X
Safety report[8]										X					X
Total blood volume collect[17]	12.5	8.5	61	0	223					134					61

[1] piperaquine (480mg)

[2] piperaquine (960mg) or sulfadoxine-pyrimethamine (1000mg/50mg) according to treatment arm

[3] Hemoglobin, hematocrit, platelets, red blood cell count, white blood cell count + differentiation

[4] Creatinine, urea, sodium, potassium, bilirubin, AF, γGT, AST, ALT, LDH and hs-Trop-T. Additional at screening: cholesterol, triglyceride + glucose

[5] HIV, HBV, HCV, *P. falciparum(screening only)*

[6] 18S qPCR for blood stage *P. falciparum*. Additional thick blood smear only on indication, blood will be taken from qPCR samples.

[7] Pfs25 qRT-PCR for sexual stage *P. falciparum.*
[8] A safety report will be compiled upon all subjects having completed day 21 after challenge and at the end of the study.
[9] See treatment criteria section 9 Methods
[10] On indication. Performed at treatment visit only when volunteers are qPCR positive.
[11] Within 4-12 hours after treatment with piperaquine
[12] On C+6, C+8, C+10, T1, T1+2, T+4, T+6, T2, T2+2, T2+4, T2+6, T3, T3+2,T3+6, ET+2, and EOS
[13] On evening visits from day 5 until T1, and at DMFA timepoints. Additional thick blood smear only on indication, blood will be taken from qPCR samples.
[14] On day 21, 24 and between day 21-36 (based on the height of gametocytemia as measured by Pfs25 qRT-PCR)
[15] Toxicology urine tests at screening and inclusion visits only. Pregnancy test for female subjects at screening and baseline visits only,
[16] Only SAE's that are possibly or probably related to trial will be collected prior to inclusion.
[17] Total blood volume: 500ml
[18] Additional amylase will be measured on screening, baseline and T1+2
[19] Subjects will be tested for RBC alloantibodies (Rh(CDE) Kell typing, and antibody screening) at screening and at the end of the study

9.4. Withdrawal of individual subjects

Subjects can leave the study at any time for any reason if they wish to do so without any penalty or loss of medical benefits.

The investigator can decide to withdraw a subject from the study for urgent medical reasons.

Volunteers can be withdrawn from the study procedures at the discretion of the clinical investigator or the local safety monitor for the following reasons:

- Any serious adverse event
- Any adverse event that, according to clinical judgment of the investigator, is considered as a definite contraindication to proceeding with the study procedures.
- The use of concomitant, chronic medication active on the immune system (e.g. steroids, immunosuppressive agents) or with known antimalarial activity against *P. falciparum*
- Pregnancy
- Withdrawal of informed consent by volunteer
- Completely lost to follow-up
- Ineligibility (either arising during the study or retrospectively, having been overlooked at screening).
- If, on balance, the investigator or safety monitor believes that continuation would be detrimental to the subject's well-being
- Volunteer non-compliance with study requirements.
- Any other protocol deviation that results in a significant risk to the subject's safety

If a subject withdrawal occurs for any reason, the investigator must make every effort to determine the primary reason for a subject's withdrawal from the study and record this information in the study file. However, in accordance with the principles of the current version of the Declaration of Helsinki, a subject does have the right to withdraw from the study at any time and for any reason, and is not obliged to give his or her reasons for doing so.

If it is felt that inclusion of the study subject's data for analysis is compromised, the subject will be terminated from the study and data will not be included in analysis. This does not preclude the ethical responsibility of the investigators to ensure the safety of the subject and ensure they receive curative therapy for malaria (Malarone), and follow the subject for cardiac manifestations of disease. All data generated before withdrawal will be included in final study analysis. Blood samples collected before withdrawal will be used/stored unless the subject specifically requests otherwise.

For subjects who are lost to follow-up (i.e. those subjects whose status is unclear because they fail to appear for study visits without stating an intention to withdraw), extensive effort (i.e. documented phone calls and e-mails) will be undertaken to locate or recall him or at least to determine his health status. The investigator should show "due diligence" by documenting in the source documents steps taken to contact the subject, e.g. dates of telephone calls, registered letters, etc.

9.5. Replacement of individual subjects after withdrawal

If a subject withdraws before or on inclusion day he/she will be replaced with an alternate volunteer who passed screening, if possible.

9.6. Follow-up of subjects withdrawn from treatment

In the event that a volunteer discontinues the study for any reason, he/she will be required to complete all safety follow-up as appropriate, as determined by the principal investigator. All volunteers who have been exposed to the bites of infectious mosquitoes are required to take a curative regimen of Malarone® (or alternative effective anti-malarial treatment should Malarone® be contra-indicated, at the discretion of the clinical investigator).

9.7. Premature termination of the study

The study may be discontinued by the sponsor:

- On advice of the safety monitor
- On advice of the Safety Monitoring Committee (SMC)
- On advice of the clinical investigator
- On advice of the CCMO

The safety monitor, SMC, CCMO or investigators may decide to put the study on hold based on adverse events, pending discussion with the Sponsor / SMC / CCMO / safety monitor / investigators. Following discussion, it may be decided to terminate the study. Safety reporting procedures are described in section 10.

10. SAFETY REPORTING

10.1. Temporary halt for reasons of subject safety

In accordance to section 10, subsection 4, of the WMO, the sponsor will suspend the study if there is sufficient ground that continuation of the study will jeopardise subject health or safety. The sponsor will notify the CCMO without undue delay of a temporary halt including the reason for such an action. The study will be suspended pending further review by the accredited METC, except insofar as suspension would jeopardise the subject's health. The investigator will take care that all subjects are kept informed.

PATH REC and WIRB will also be notified of any decisions to prematurely suspend or terminate the study.

10.2. AEs, SAEs and SUSARs

10.2.1. Adverse events (AEs)

Adverse events are defined as any undesirable experience occurring to a subject during the study, whether or not considered related to a trial procedure or the experimental intervention. All adverse events reported spontaneously by the subject or observed by the investigator or his or her staff will be recorded.

Abnormal laboratory findings (e.g. clinical chemistry or haematology) or other abnormal assessments that are judged by the investigator to be clinically significant will be recorded as AEs (or SAEs if they meet the definition). The investigator will exercise his or her medical and scientific judgement in deciding whether an abnormal laboratory finding or other abnormal assessment is clinically significant.

If there are any severe complaints not typical for malaria infection, such as chest pain, the volunteer will be evaluated immediately by a qualified clinician using the appropriate clinical assessments (e.g. ECG or measurement of cardiac enzymes) according to standard hospital care.

10.2.2. Serious adverse events (SAEs)

A serious adverse event is any untoward medical occurrence or effect that

- results in death;
- is life threatening (at the time of the event);
- requires hospitalisation or prolongation of existing inpatients' hospitalisation;
- results in persistent or significant disability or incapacity;
- is a congenital anomaly or birth defect; or
- any other important medical event that did not result in any of the outcomes listed above due to medical or surgical intervention but could have been, based upon appropriate judgement by the investigator.

An elective hospital admission will not be considered as a serious adverse event.

The investigator will report all SAEs to the sponsor and the safety monitor without undue delay after obtaining knowledge of the events (within 24 hours). All SAEs will be reported through the web portal *ToetsingOnline* to the CCMO, within 7 days for SAEs that result in death or are life threatening followed by a period of maximum of 8 days to complete the initial preliminary report by the investigator/sponsor. All other SAEs will be reported within a period of maximum 15 days after the sponsor has first knowledge of the serious adverse events.

10.2.3. Suspected unexpected serious adverse reactions (SUSARs)

Adverse reactions are all untoward and unintended responses to an investigational product related to any dose administered.

Unexpected adverse reactions are SUSARs if the following three conditions are met:

1. the event must be serious (see chapter 9.2.2);
2. there must be a certain degree of probability that the event is a harmful and an undesirable reaction to the medicinal product under investigation, regardless of the administered dose;
3. the adverse reaction must be unexpected, that is to say, the nature and severity of the adverse reaction are not in agreement with the product information as recorded in the Summary of Product Characteristics (SPC) or investigational brochure (IB).

The sponsor will report expedited all SUSARs through the web portal *ToetsingOnline* to the CCMO. The expedited reporting of SUSARs through the web portal *ToetsingOnline* is sufficient as notification to the competent authority. The expedited reporting will occur not later than 15 days after the sponsor has first knowledge of the adverse reactions. For fatal or life threatening cases the term will be maximal 7 days for a preliminary report with another 8 days for completion of the report.
As this is an open label study in which the sponsor, investigator and the SMC are not blinded to treatment allocation, the code would not have to be broken in the case of a SUSAR.

Any SAEs, SUSARs or AEs that suggest the research places subjects or others at a greater risk of harm (including physical, psychological, economic, or social harm) than was previously known or recognized, or that require a change to the protocol or consent will be reported to PATH REC and/or WIRB in accordance with the reporting requirements of each.

10.3. Follow-up of (serious) adverse events

10.3.1. Adverse event data collection

Safety assessments will be performed and recorded by the investigators. All adverse events/reactions (solicited and unsolicited), noted by the investigators will be accurately documented in the case report form by the investigators. For each event/reaction the following details will be recorded:

1. Description of the event(s)/reaction(s)
2. Date and time of occurrence
3. Duration
4. Intensity
5. Relationship with the intervention
6. Action taken, including treatment
7. Outcome

In addition, symptoms will be ranked as (1) mild, (2) moderate, or (3) severe, depending on their intensity according to the following scale:

- Mild (grade 1): awareness of symptoms that are easily tolerated and do not interfere with usual daily activity
- Moderate (grade 2): discomfort that interferes with or limits usual daily activity
- Severe (grade 3): disabling, with subsequent inability to perform usual daily activity, resulting in absence or required bed rest

If an AE changes in intensity during the specified reporting period, a new description of the AE will be added.
When an AE/SAE occurs, it is the responsibility of the investigators to review all documentation (e.g. hospital progress notes, laboratory, and diagnostics reports) related to the event. The investigators will then record all relevant information regarding an AE/SAE on the CRF or SAE Report Form, respectively. The investigator will attempt to establish a diagnosis of the event based on signs, symptoms, and/or other clinical information. In such cases, the diagnosis should be documented as the AE/SAE and not the individual signs/symptoms.

The investigators will keep record of a clinical symptom score per volunteers (Appendix 1) during the blood stage challenge to assess if treatment is needed be initiated (see treatment criteria section 9.3).

10.3.2. Assessment of causality

The investigators are obligated to assess the relationship between study procedures and the occurrence of each AE/SAE. The investigators will use clinical judgment to determine the relationship. Alternative causes, such as natural history of the underlying diseases, concomitant therapy, other risk factors and the temporal relationship of the event to the challenge will be considered and investigated. The relationship of the adverse event with the study procedures will be categorized as:

Probable	An adverse event that follows a reasonable temporal sequence from the study procedure and cannot be reasonably explained by the known characteristics of the subject’s clinical state.
Possible	An adverse event for which insufficient information exists to exclude that the event is related to the study procedure.
Not related	An event for which sufficient information exists to indicate that the aetiology is unrelated either because of the temporal sequence of events or because of the subject’s clinical state or other therapies.

10.3.3. Follow-up of adverse events

All AEs will be followed until they have abated, or until a stable situation has been reached. AEs that result in a subject’s withdrawal from the study or that are present at the end of the study will be followed up (if the volunteer consents to this) until a satisfactory resolution or stabilisation occurs, or until a non-study related causality is assigned.
Depending on the event, follow up may require additional tests or medical procedures as indicated, and/or referral to the general physician or a medical specialist.
AEs and SAEs will be reported until end of study within the Netherlands, defined as the last patient visit.

10.4. Local Safety Monitor (LSM) and Safety Monitoring Committee (SMC)

10.4.1. Local safety monitor

For this study, a local safety monitor will appointed, who is based in the Raboudumc and will be involved in the review of severe and serious adverse events and volunteer safety. He/she is an

experienced clinician qualified to evaluate safety data from clinical studies with malaria infections. He/she is independent of the sponsor and the investigators.

10.4.2. Safety Monitoring Committee (SMC)

An independent Safety Monitoring Committee (SMC) will be appointed, including at least 3 individuals. Their main responsibility will be assessing any severe or serious adverse events and, if necessary, halting further study procedures. A safety report including a list of all reported adverse events and any safety laboratory values outside the normal range will be prepared for all groups upon day 21 by all subjects and at the end of the study. For further details see section 9.3.17.
The advice(s) of the SMC will only be sent to the sponsor of the study and the funding partner, PATH. Should the sponsor decide not to fully implement the advice of the SMC, the sponsor will send the advice to the CCMO, including a note to substantiate why (part of) the advice of the SMC will not be followed.

10.4.3. Review of safety data by the safety monitor and SMC

A safety report including a list of all reported adverse events and any safety laboratory values outside the normal range will be prepared upon all subjects having completed the day 21 visit after the malaria challenge infection and at the end of the study.
These reports will be prepared by a clinical investigator and sent to the safety monitor, all clinical investigators involved and the funding partner, PATH. The safety monitor will review the safety data within 2 workdays and if warranted instruct the site to take appropriate action.
In addition, safety data for all participants will be assessed by the SMC at the end of the study. Responsibilities of the SMC are described in the SMC Charter. The advice(s) of the SMC will only be communicated to the CCMO when the sponsor does not follow this. With this notification a statement will be included indicating whether the advice will be followed.
Any highly sensitive troponin T value greater than 60ng/l will be reported to the safety monitor within 24 hours. Any safety laboratory values that lead to immediate malaria treatment will be reported to the safety monitor within 24 hours.

10.4.4. Safety stopping rules

The study may be placed on safety hold for the following reasons:

- On advice of the safety monitor
- On advice of the Principal/Clinical investigators
- On advice of the SMC
- On advice of the CCMO
- One or more participants experience a SAE that is determined to be related to the study intervention
- Two or more grade 3 adverse events in the same group of subjects, which are unexpected and possibly, probably or definitively related to the study intervention.
- Any clinical cardiac event that does not meet the criteria of SAE

The safety monitor, CCMO, or investigators may decide to put the study on hold based on adverse events, pending discussion with the safety monitor, CCMO, and investigators. In addition, the PI can always decide based on characteristics, duration and severity of signs/symptoms to treat and stop the trial for individual cases. The PI will identify when stopping rule criteria are met and alert the

appropriate parties. The safety monitor will review all available safety data on a pre-defined time point after the challenge period. If the CCMO has recommended safety hold, re-initiation of the study will require CCMO concurrence. The CCMO, PATH REC and WIRB will be informed of a safety hold by the sponsor. Following discussion, it may be decided to terminate the study.

11. STATISTICAL ANALYSIS

11.1. Primary study parameter(s)

All adverse events for each volunteer will be tabulated. Adverse events will be analyzed by calculating the proportion of volunteers in each group who report mild, moderate or severe adverse events. The frequency of signs and symptoms will be compared between groups with the chi-square test.

Any clinically important deviations from normal occurring in routine laboratory test results and/or vital signs as determined by the investigator will be listed.

Clinical laboratory data (haematology, blood chemistry, and urinalysis) which is outside of the normal range will be listed in tables. Isolated laboratory abnormalities will be reported as AEs if they are considered to be clinically significant by the Investigator. Vital signs which are outside of the normal range and clinically significant will also be listed in tables. All adverse events will be listed by participant and will include details of the treatment received prior to onset, onset time, duration, severity and relationship to the study drug.

In this trial, we will determine the suitability of our treatment strategies to induce gametocytemia, defined as gametocyte prevalence by Pfs25 qRT-PCR on any moment during follow-up and to achieve infectiousness to mosquitoes. Based on preliminary data, we expect >95% individuals will develop gametocytes after commencing treatment with sulphadoxine-pyrimethamine(37) or piperaquine(25). In CHMI-trans1, 100% (16/16) individuals developed gametocytes. The CHMI-trans approach is considered unsuitable if <50% of individuals develop mature gametocytes; the lower limit of the confidence interval around the proportion of gametocyte positive individuals should thus be above 50%. If we enroll 12 individuals of whom 11 become gametocyte positive, the lower limit of the 99% confidence interval around this proportion is 52%. We anticipate that ~73% of the individuals who are exposed to Pf by blood stage inoculation will infect at least one mosquito (Collins, unpublished data). Within each of the inoculation means (mosquito inoculation versus blood stage, 12 participants each) we may thus expect 8 or 9 infectious individuals and consequently a lower limit of the 95% confidence interval around the proportion of infectious individuals ≥34%.

Comparisons between groups are underpowered by conventional frequentist approaches and a Bayesian model will be used to select the challenge and treatment strategy with evidence for highest gametocyte prevalence, density, infectiousness prevalence and mosquito infection rates.

The aim of this Bayesian analysis is not to test for statistical significance of subtle differences in gametocyte density or mosquito infection rates per arm but rather select the inoculation and treatment strategy with the highest level of evidence of sufficiently high gametocyte densities and mosquito infection rates to allow the CHMI trans model to be used for the evaluation of transmission-blocking interventions.

11.2. Secondary study parameter(s)

Demographic data will be summarized by descriptive statistics and will include total number of observations (n), mean, standard deviation (SD) and range for continuous variables and number and % with characteristics for dichotomous variables.

11.3. Other study parameters

In immunological analyses we will assess differences by comparing mean values between groups or time points using either a two-tailed student's t-test (if comparing two groups) or a one-way ANOVA (if comparing more than two groups) or non-parametric equivalents. Paired tests will be used if pre-intervention values are compared with post intervention values, unpaired tests will be used if comparisons are made between groups. For discrete variables (e.g. the number of positive assays), the chi-squared test or Fisher's exact test will be used (two-tailed).

12. ETHICAL CONSIDERATIONS

12.1. Regulation statement

This study will be conducted in accordance with the latest Fortaleza revision of the Declaration of Helsinki (2013), the Medical Research Involving Human Subjects Act (WMO), the ICH Good Clinical Practice, and local regulatory requirements.

The investigators are responsible for obtaining all relevant Ethics Committee (EC) / Institutional Review Board (IRB) approvals, including WIRB approval, of the protocol and any subsequent amendments in compliance with local law before the start of the study.

12.2. Recruitment and consent

As soon as the study is approved by the CCMO and WIRB, healthy volunteers will be recruited to participate in the study. Advertisements will be placed in prominent places on university campuses and other public places as well as on the intranet of the Radboud University. Furthermore, a Facebook page (link: http://www.facebook.com/malariavaccin) showing the advertisement text will be used to inform people about the trial. This brief advertisement will indicate a telephone number to call and an e-mail address for contact to request further information. It will furthermore indicate a website (www.malariavaccin.nl) which contains a form. This short questionnaire will be completed using the online form. When seemingly suitable volunteers contact investigators via e-mail, telephone or the online form, they will be invited to an information meeting during which the study will be explained to them by the study investigator. Directly after the meeting they will be provided with documents to review at home (the information sheet, the informed consent form, the application form and the insurance text). During and after the meeting there will be time for questions. After this free discussion with the investigator, and any follow-up discussion if necessary, the volunteer will be given sufficient time to consider participation.

Volunteers who are interested in participating will be asked to fill in the application form and will be invited to come for a screening visit. Eligible subjects may only be included in the study after providing written, CCMO- and WIRB-approved informed consent. Informed consent must be obtained before conducting any study-specific procedures (i.e. all of the procedures described in the protocol, including screening procedures). The process of obtaining informed consent should be documented in the subject source documents. During the screening visit, the questionnaire answers will be discussed and inclusion and exclusion criteria will be checked. Also, a letter for the general practitioner will be signed and sent after screening. Again, the investigator will answer any questions the volunteer has. The possibility of withdrawal from the study, at any time, without penalty and without any declaration of the reason will be pointed out to the volunteers.

The investigators will be responsible for providing adequate verbal and written information regarding the objectives and procedures of the study, the potential risks involved and the obligations of the volunteers. Volunteers will be informed that they will not gain health benefits from this study. Trainees or other students who might be dependent on the investigators or the study group will not be included in the study.

12.3. Benefits and risks assessment, group relatedness

Two major areas of ethical concern are contained within this proposal, namely the use of blood from humans and the use of human volunteers for *P. falciparum* CHMI. All partners in this proposal are aware of and follow the relevant national and international rules and regulations as they pertain to

access to material of human origin and clinical research. International agreements such as the Declaration of Helsinki will be observed and respected.

12.4. Ethical aspects concerning the production of P. falciparum infected mosquitoes

The human blood and serum used for parasite culture is obtained from screened healthy blood donors from Sanquin. Continuous culture of the drug sensitive *P. falciparum* strain NF54/3D7 has been routine for the Central Animal Facility of the Radboudumc over the past 3 decades. All culture material is checked for bacterial contamination, including Mycoplasma, and for blood-borne pathogens (HIV, HBV, HCV, HTLV 1+2). The process has become highly standardized as described in standard operating procedures and was positively reviewed by an external auditor in 2014.

12.5. Ethical aspects concerning the use of human volunteers

Infection of humans with malaria has been carried out for nearly a century, including for therapeutic use as treatment for neurosyphilis and later for drug and vaccine evaluation. The ability to carry out this type of work is largely based on the relatively low morbidity and the lack of mortality seen in these studies since the advent of feeding mosquitoes on *P. falciparum* gametocyte cultures in 1986 (28). The occurrence of three cardiac events in volunteers participating in phase I/IIa malaria vaccine trials in Nijmegen has raised discussion about the safety of malaria challenge trials with respect to cardiac events. Based on recommendations of the CCMO and an External Scientific Advisory Committee to the European Malaria Vaccine Development Association, this malaria challenge trial protocol has been adjusted (see also section 1.4 and 14). In 2016 the occurrence of mild, transient liver injury and transient increased creatinine levels during CHMI was extensively evaluated. Based on recommendations of the CCMO and external experts, the malaria challenge protocol was modified to include extra monitoring (see also section 1.4 and 14).

Testing in human subjects remains the only reliable and convincing way to obtain information on the immunological responses that are important for protection against malaria. Of course, the compelling need for a malaria vaccines and treatments needs to be balanced with the potential risks and discomforts of the volunteers. Explorative studies looking for new or complementary transmission blocking drugs or vaccines are of paramount importance with the potential of large-scale application in endemic countries. These transmission-blocking interventions are considered essential tools to consolidate recent gains of malaria control in recent years and move towards malaria elimination.

The study will be undertaken in accordance with Good Clinical Practices (GCP), according to the standards defined in the EEC directive 91/507/EEC, and in the Directive on Good Clinical Practice in Clinical Trials (ICH GCP, 75/318/EEC, January 1997) and under the principles of the Declaration of Helsinki; ethical permission will be sought from the CCMO the Netherlands and from the funding partner’s designated IRB, WIRB..

12.6. Compensation for injury

The sponsor/investigator has a liability insurance which is in accordance with article 7 of the WMO.
The sponsor (also) has an insurance which is in accordance with the legal requirements in the Netherlands (Article 7 WMO). This insurance provides cover for damage to research subjects through injury or death caused by the study.

1. € 650.000,-- (i.e. six hundred and fifty thousand Euro) for death or injury for each subject who participates in the research;
2. € 5.000.000,-- (i.e. five million Euro) for death or injury for all subjects who participate in the research;
3. € 7.500.000,-- (i.e. seven million five hundred thousand Euro) for the total damage incurred by the organisation for all damage disclosed by scientific research for the sponsor as 'verrichter' in the meaning of said Act in each year of insurance coverage.

The insurance applies to the damage that becomes apparent during the study or within 4 years after the end of the study.

12.7. Incentives

Enrolled and challenged volunteers will receive up to 1900,- Euros in compensation for their time and for the inconveniences of taking part in this study. This is based on compensation fees for procedures as below:

- Inconvenience of blood tests and/or visits: 20,- Euros per blood sampling and/or visit
- Malaria challenge: 300,- Euros
- Illness and treatments compensation: 400,- Euros
- Direct Feeding Assays: 300,- Euros
- Compensation length of study: 20,- Euros per month

Travel expenses will not be additionally reimbursed, and compensation will not be provided to volunteers who are not enrolled i.e. screen failures. Eligible volunteers who are enrolled at the inclusion visit as back-ups, but who are not challenged on Day 0 will be compensated 50,- Euros. These compensation amounts are reasonable and in line with Dutch common practice. In case of unexpected medical complications, there will be access to state-of-the-art medical treatment with full costs covered by the insurance of Radboudumc.

13. ADMINISTRATIVE ASPECTS, MONITORING AND PUBLICATION

13.1. Handling and storage of data and documents

13.1.1. Confidentiality

All parties agree to adhere to the principles of medical confidentiality in relation to clinical study subjects involved in this trial, and shall not disclose the identity of subjects to third parties without prior written consent of the subject.
All data will be anonymised; volunteer data will be identified by a unique study number in the CRF. Separate confidential files containing identifiable information will be stored in secured locations. All plasma samples, or other biological samples, with exception of those taken for safety diagnostics, will be labelled with the volunteer study identification number. Samples taken for safety diagnostic (processed by the central clinical laboratory of the Radboudumc) will be labelled with part of the subject identification code, study identification name and a fictitious birth date (only using the subjects actual birth year). The samples will not be labelled with volunteer names or actual birth dates.
The subject identification code will be kept by the principal investigator.
The investigator will maintain and retain appropriate medical and research records and essential documents for this trial in compliance with ICH GCP Guidelines, any regulatory requirements, and institutional requirements for the length of storage and for the protection of confidentiality of volunteers. The investigator will permit direct access to study records and source documents to authorized representatives of the sponsor, ethical committee(s) / institutional review board(s), regulatory agencies, authorised individuals from PATH, and the external monitor(s), for the purposes of quality assurance reviews, audits / inspections, and evaluation of the study safety and progress. Direct access includes examination, analysis, verification, and reproduction of de-identified records and reports that are important to the evaluation of the trial. Data and biological samples will be stored for 15 years. Biological samples from this study will be stored for 15 years for research related to malaria immunology and transmission dynamics. New immunological or molecular tests may become available in the future that could strengthen or validate this research or help find important new findings. Samples will only be used for malaria-related research.

13.1.2. Data collection

Designated trial staff will enter the data required by the protocol into the electronic CRF (eCRF).

13.1.3. Database management and quality control

All data should be recorded, handled and stored in a way that allows its accurate reporting, interpretation and verification.
An external monitor will review the data entered into the eCRFs by investigational staff for completeness and accuracy and will instruct the site personnel to make any required corrections or additions. Queries are made during each monitoring visit. Designated investigator site staff are required to respond to the queries and confirm or correct the data. Medical history/current medical conditions and adverse events will be coded using the ICD-10 terminology.

13.2. Monitoring and Quality Assurance

Before study initiation, the protocol and eCRFs together with relevant SOPs will be reviewed by the sponsor, the investigators and their staff. During and after completion of the study, the data monitor

will visit the site to check the completeness of records, the accuracy of entries on the eCRFs, the adherence to the protocol and to Good Clinical Practice, the progress of enrolment, and to ensure that sulfadoxine-pyrimethamine, piperaquine and Malarone® and are being dispensed and accounted for according to protocol.
The investigator will maintain source documents for each subject in the study, consisting of case and visit notes containing demographic and medical information, laboratory data, electrocardiograms, subject’s diaries, and the results of any other tests or assessments. All information on eCRFs must be traceable to these source documents in the subject's file. The only exception is the data from the quantitative PCR, which is loaded from the PCR machine directly into the eCRF. As with all parts of the eCRF, there is an audit trail in place to register every data entry. The investigator will also keep the original informed consent form signed by the subject (a signed copy is given to the subject).
The investigator will give the external monitor access to all relevant source documents to confirm their consistency with the eCRF entries. According to the NFU risk classification system, this clinical trial has been classified as ‘middle risk’. The monitor will perform full verification for the presence of informed consent, adherence to the inclusion/exclusion criteria and documentation of SAEs. The recording of data that will be used for all primary and safety variables will be assessed for 25% of included subjects (i.e. 8 subjects).

13.3. Amendments

Amendments are changes made to the research, and will only be made after favourable opinions / approvals by the CCMO and WIRB have been given - except where necessary to eliminate apparent immediate hazards to the subject(s). All amendments will be submitted to CCMO and WIRB for review and approval.

13.4. Annual progress report

The investigator will submit a summary of the progress of the trial to the CCMO once a year. Information will be provided on the date of inclusion of the first subject, numbers of subjects included and numbers of subjects that have completed the trial, serious adverse events/ serious adverse reactions, other problems, and amendments.
Continuing review reports will be submitted to WIRB, in accordance with its reporting requirements.

13.5. Temporary halt and (prematurely) end of study report

The investigator will notify the CCMO of the end of the study within a period of 8 weeks. The end of the study is defined as the subject’s last visit on day 51 after the malaria challenge infection.
The investigator will notify the CCMO immediately of a temporary halt of the study, including the reason of such an action. In case the study is ended prematurely, the investigator will notify the CCMO within 15 days, including the reasons for the premature termination.
Within one year after the end of the study, the investigator will submit a final study report with the results of the study, including any publications/abstracts of the study, to the CCMO.
PATH REC and WIRB will also be notified of any decisions to prematurely suspend or terminate the study, and a study closure report will be submitted to WIRB when all subjects have finished their final visits and follow-up and when the sponsor has indicated the study is closed.

13.6. Public disclosure and publication policy

The final report will be prepared by the investigators at the Radboud university medical center. It will be signed by the project leader or the principal investigator. The investigators will make every effort to publish the results in a peer-reviewed journal.

14. STRUCTURED RISK ANALYSIS

Controlled Human Malaria Infection

14.1. Potential issues of concern

In this study all volunteers undergo a Controlled Human Malaria Infection by the bites of 5 laboratory reared *Anopheles* mosquitoes infected with the 3D7 clone of *Plasmodium falciparum* or a blood stage challenge with 3D7 infected erythrocytes.

<u>a. Level of knowledge about mechanism of action</u>

The causative organisms of malaria, *Plasmodium* parasites, were first identified by Laveran in 1880 and their complete life-cycle in the mammalian host was elucidated by 1947. Extensive clinical experience of malaria infection has since been accumulated by the medical community. Nevertheless, certain aspects of the pathophysiology of (severe) malaria (e.g. cerebral malaria) remain incompletely understood. Such manifestations, however, do not occur during Controlled Human Malaria Infections due to the very early treatment of study subjects and hence the clearance of parasitemia at extremely low levels (see also below).

<u>b. Previous exposure of human beings with the test product(s) and/or products with a similar biological mechanism</u>

There is extensive clinical experience with controlled human *P. falciparum* malaria infections by the bite of infected mosquitoes or a blood stage challenge with infected erythrocytes. Since 1986 more than 3,500 volunteers have had CHMI by the bites of mosquitoes fed on cultures of *P. falciparum* gametocytes to produce *P. falciparum* sporozoites or a blood stage challenge with infected erythrocytes. Worldwide the majority of these infections have been performed with the laboratory NF54 strain or its daughter clone 3D7. This has proved to be a reproducible, predictable and safe method of inducing *P. falciparum* infections. The results of such studies were summarized in 1997 (11), in 2007 (29) and in 2011-2012 (88) (27).

<u>c. Can the primary or secondary mechanism be induced in animals and/or in *ex-vivo* human cell material?</u>

In vitro assays cannot capture the complexity of the multi-stage life-cycle of *Plasmodium* parasites and their complex of interaction with the host. Although (parts of) the *Plasmodium* life-cycle can be reproduced in (rodent or primate) animal models, these either involve non-human *Plasmodium*-species or non-natural host-parasite combinations. As a result, extrapolation of pathophysiological and immunological aspects of infection to human disease are only possible to a limited extent (89, 90).

<u>d. Selectivity of the mechanism to target tissue in animals and/or human beings</u>

The life-cycle of malaria parasites follows a fixed and pre-determined course in the human host, including migration of sporozoites from the skin to the liver sinusoids, intra-hepatic development, asexual multiplication and finally gametocytogenesis within erythrocytes. These developments are constrained by multiple parasite-host ligand interactions and the parasite’s ability to manipulate the host cell’s internal environment (91).

e. Analysis of potential effect

Safety and tolerability of CHMI will be assessed by recording adverse events, reported at any time during the trial either at a visit or by writing in the diary. Temperature will be recorded at every visit following the CHMI. Assessment of complete blood count, LDH and troponin-T will be performed daily starting on day 4-6 after the CHMI. Assessment of liver and kidney function will be performed on multiple timepoints (see section 1.4 above) and at the end of study visit. Parasitemia in subjects undergoing CHMIs will never exceed ±0.001% (i.e. equivalent to 50 parasites/µL, the detection level of thick smear microscopy is ~5 parasites/µL). In contrast, manifestations of severe malaria generally only occur above 5% parasitemia and by definition never below 1% parasitemia (WHO Guideline Severe Malaria, SWAB Richtlijn).

f. Pharmacokinetic considerations

Not applicable.

g. Study population

Included subjects are healthy young adult volunteers, who have been extensively screened for any evidence of co-morbidity, in particular cardiovascular risk factors. Female subjects of child-bearing age are screened for pregnancy by urine test and are required to use contraception throughout the study period, as described in section 9.

h. Interaction with other products

Concurrent use of drugs potentially interacting with sulfadoxine-pyrimethamine (e.g trimpethoprim, trimethoprim-sulfonamide combinations, chloroquine, lorazepam, phenytoin, coumarin derivatives, folic acid antagonists), piperaquine (e.g. QTc-prolonging medication, strong CYP3A4 inhibitors, rifampicin, carbamazepine, phenytoin, phenobarbital, St.
John's wort (Hypericum perforatum)) and atovaquone-proguanil (e.g. artemether-lumefantrine, rifampicin, metoclopramide, oral anti-coagulants and certain anti-retrovirals) are contra-indicated.
No adverse drug interactions have been described between piperaquine, sulfadoxine-pyrimethamine and/or atovaquone-proguanil(92-95). Piperaquine has been combined with sulfadoxine-pyrimethamine in clinical trials for intermittent preventive treatment and in CHMI-trans1, and is safe, well tolerated, and effective (96, 97).
The relatively long half-life of sulfadoxine-pyrimethamine and, especially, piperaquine might potentially cause a low concentration of either of these drugs at the time of final Malarone® treatment. However, no adverse interactions between these drugs have been described nor are expected to occur in these doses (93-95, 98). Piperaquine is mainly metabolised by CYP3A4 and to a lesser extent by CYP2C9 and CYP2C19. Piperaquine is an inhibitor of CYP3A4 (also in a time-dependent manner) and to a lesser extent of CYP2C19, while it induced CYP2E1(95). In Malarone®, there is no evidence that atovaquone (the primary effective anti-malarial component) is metabolised and there is negligible excretion of atovaquone in urine with the parent drug being predominantly (> 90%) eliminated unchanged in faeces. Proguanil hydrochloride is partially metabolised with less than 40% being excreted unchanged in the urine. Proguanil is metabolized to cycloguanil (primarily via CYP2C19) and 4-chlorophenylbiguanide, and these are also excreted unchanged in the urine. Proguanil also has antimalarial activity independent of its metabolism to cycloguanil, and proguanil, but not cycloguanil, is able to potentiate the ability of atovaquone to collapse mitochondrial membrane potential in malaria parasites. During administration of Malarone® at recommended

doses, proguanil metabolism status appears to have no implications for treatment or prophylaxis of malaria(93).
Therefore, it is highly unlikely that low concentration piperaquine would affect the effectivity or safety of Malarone® in treatment of malaria.

i. Predictability of effect
In this trial all volunteers will experience blood stage malaria infection. Blood stage malaria infection has been seen after CHMI in over 3,500 volunteers. The progression and symptoms of this parasitemia has been demonstrated to be reproducible and predictable (11, 29, 30). However, the occurrence of cardiac serious adverse events in Radboudumc CHMI trials has led to an increase in safety measures, discussed in a separate section below.

j. Can effects be managed?
Subjects are followed up intensively on an outpatient basis for clinical and parasitological assessment to ensure treatment is started at the earliest possible time point. The 3D7 *P. falciparum* clone has been tested for sensitivity to sulfadoxine-pyrimethamine, piperaquine and atovaquone-proguanil *in vitro*. Should treatment with this drug need to be discontinued prematurely in any subject for whatever other reason (e.g. intolerability), various other anti-malarial drugs are available, both oral and intravenous, to which this clone is also susceptible.

14.2. Cardiac events following Controlled Human Malaria Infections

In the twenty-three CHMI studies conducted at Radboudumc (involving 376 healthy volunteers), three cardiac events have occurred after infection with the NF54 strain with the confirmed or differential diagnosis of myocarditis. The occurrence of myocarditis in both natural uncomplicated malaria and CHMI has never been described elsewhere. However, the three cardiac events share no specific factor other than malaria infection.
With regards to the malaria infection, these myocarditis cases do share a number characteristics: i) they took place 1-5 days after start of antimalarial treatment but different anti-malarial drugs were used in all cases ii) there was no parasitemia detectable at the time of the event, and iii) next to the P. falciparum infection, other and known possibly triggering factors (e.g. preceding vaccinations, concomitant infections, cannabis use) were present during or preceding the event. The three cases are discussed in detail below.

Case 1

In 2007 a 20 year old female was immunized with the candidate malaria vaccine, LSA3 (NL14715.000.06), underwent a CHMI and was treated with artemether/lumefantrine (Riamet®) after a positive thick smear. She presented with retrosternal chest pain three days after treatment, blood slide was negative. Elevated troponin-I (maximally 11.80ug/l) and ECG abnormalities were suggestive for a cardiac event with minimal cardiac damage and an MRI showed no abnormalities. Risk factors for cardiac disease in this volunteer included an acute myocardial infarction in a paternal grandfather at age 43 and a paternal family history of dyslipidemia. The final diagnosis was acute coronary syndrome or myocarditis. This case is reported in (35), attachment K4a. Following this event, recommendations of the European Malaria Vaccine Development Association and the CCMO followed for improved safety of participating volunteers:

- Riamet® is no longer used as treatment during CHMIs at Radboudumc. Malarone® replaces it.
- Individuals will be excluded from participation if they have 1st or 2nd degree relatives who had cardiac events when less than 50 years of age.
- Volunteers will be required to stay very close to the Radboudumc to ensure maximal safety from day 5 after CHMI until treatment has been finished (maximum 12 days).
- Monitoring of highly sensitive troponin T (hsTropT), D-dimer, lactate dehydrogenase (LDH) and thrombocytes.

Case 2

In 2013, a healthy male, taking part in the TIP5 clinical trial (NL39541.091.12) underwent a CHMI challenge after immunization with cyropreserved sporozoites under chloroquine prophylaxis (CPS protocol). Thick smear became positive after challenge infection and the volunteer was treated with atovaquone/proguanil (Malarone®). Per protocol blood examination revealed an elevated troponin-T (maximally 1115ng/l) on the second day of Malarone treatment, while the volunteer was asymptomatic. He was admitted to cardiology and troponin-T values, ECG abnormalities and a cardiac MRI confirmed the diagnosis acute myocarditis. During admission he experienced a 20 minute episode of retrosternal chest pain. Further diagnostic tests and patient history revealed a concurrent rhinovirus infection and a boosting regime of standard travel vaccines 46 days preceding the CHMI, with a booster 20 days before the CHMI. This case has been reported in (36), attachment K4b. Due to this SAE, new safety recommendations were integrated into our procedures for CHMI. These include:

- Exclusion of volunteers who took standard vaccinations within 3 months before the start of the trial or are planning to take standard vaccinations during the trial period up to 8 weeks after CHMI.
- Increased control of hs troponin T as a marker of cardiac damage; initiation of treatment with Malarone when hs troponin T > 0.1 µg/ml or on recommendation of the cardiologist.
- Continuation of daily LDH measurements starting from day 5 after CHMI; Malarone treatment will be initiated if LDH values are above 1000 U/L.
- Daily measurements of thrombocytes; volunteers will be treated with Malarone when thrombocyte levels are < 120x 10^9/L.
- After CHMI by sporozoite challenge we will rely on real-time qPCR for diagnosis of malaria and initiation of treatment, using the following criteria:
    - Two consecutive positive qPCR results in a volunteer with temperature <38.0^{o}C
    - One positive qPCR result in a volunteer with temperature ≥38.0^{o}C
    - One positive thick smear (which will be made if a volunteers attends for evening follow-up with temperature ≥38.0^{o}C or on decision of the trial clinician)

Case 3

In 2014, a healthy, 23 year old, male volunteer underwent a CHMI under chloroquine prophylaxis (CPS-immunization). On day 10 after exposure to bites of 15 malaria infected mosquitoes, routine per protocol blood examinations revealed an elevated troponin-T (maximally 168ng/l) though the volunteer was asymptomatic. This volunteer had no obvious risk factors for cardiac disease other than cigarette smoking and recent cannabis use. This volunteer was negative by thick smear but

positive by PCR with maximum parasitemia was 1265Pf/ml while under chloroquine prophylaxis. Following this cardiac event, further safety measures have been integrated into our CHMI protocols:

- Volunteers will be treated with antimalarial after a single positive qPCR after malaria challenge infection
- Use of cannabis will be added as an exclusion criterion
- Excessive physical exercise around immunization and during challenge period will be prohibited

14.3. Transient liver function test derangements

Transient, asymptomatic liver function derangements have been reported in volunteers in previous CHMI studies, and are likely to be related to the challenge infection (*Reuling et al. manuscript in preparation*). A retrospective analysis of 13 CHMI studies conducted in the Radboudumc showed that 72/120 (60%) of the volunteers that were treated at thick smear parasitemia levels have mild (38%), moderate (10%) or severe (12%) increases of liver transaminases (ALT/AST). The liver function test (LFT) elevations remained asymptomatic in all the affected volunteers, and are typically not associated with bilirubin changes. Detailed analysis on the LFT elevations pattern showed that these elevations exceeded the upper limit of normal (ULN) around the day of treatment with antimalarials to 2 days after treatment. The peak LFTs elevations are found between 2-14 days after treatment with values normalized at study end (35–42 days after challenge infection). In some volunteers, the elevations were also found associated with lactate dehydrogenase, suggesting that associated sub-clinical haemolysis might have been present during challenge.

Volunteers treated based on qPCR threshold of 100 *Pf*/mL showed a lower percentage, and severity of LFT abnormalities. 13/58 volunteers (22%) showed LFT derangements, with 11/58 (19%) mild, 1/58 (2%) moderate, and 1/58 (2%) severe abnormalities. Importantly, all volunteers showed normalized values at study end.

A clear explanation for the transient elevated transaminases in our CHMI studies is not obvious. Although higher parasitemia associates with higher chances of LFT elevations on group level, there is no clear relationship on individual levels. Given the diversity of drug regimens used, it also seems unlikely to be directly related to anti-malarials. Similarly, the timing, differential, and limited use of paracetamol does not support a clear relationship. Rather a combination of the above mentioned factors, and individual susceptibility may have triggered the observed abnormalities.

Following an independent review of the safety committee, and a hepatology expert, it was concluded that these transient asymptomatic and severe LFT elevations are most likely a direct consequence of the malaria infection rather than reflecting a direct drug-induced liver injury caused by the antimalarial drug or other liver noxe. Given the rapid and spontaneous resolution of the observations and the absence of significantly elevated bilirubin these events were judged to not cause permanent subclinical liver damage, and to not preclude further studies utilising *Plasmodium falciparum* challenge.

As a result of the LFT elevations our safety procedures for CHMI have been intensified. In the current trial, we will adhere to those stringent procedures that are relevant, including:

1. Exclusion of subjects with abnormal ALT/AST values on baseline
2. Upon inclusion: Avoid additional triggers that may cause ALT/AST elevations
    a. Alcohol intake from baseline up to 1 week post-treatment (T3)
    b. Heavy physical exercise

c. Recreational drug use
d. >3 grams/ day of paracetamol/acetaminophen
3. Standard monitoring of LFT at day* *C-1; C+6; C+8; C+10; T; T+2; T+4; T+6; EOS*
4. Initiation of subcurative anti-malarial treatment at ALT/AST levels >2.5xULN (prior T1)
5. Initiation of a curative anti-malarial treatment at ALT/AST levels >5x ULN (prior T1)

**C = challenge; T = day of treatment 1,2, and 3; EOS = end of study*

14.4. Transient serum creatinine elevations

Transient, asymptomatic increases in serum creatinine compared to baseline (though within normal limits) have also been found during CHMI in the Radboudumc. A retrospective analysis of 5 CHMI studies showed a small increase of creatinin, relative to the subject's own baseline (but within the accepted range of normal), in 70/80 (88%) of the subjects. Only 5/80 (5%) showed mild elevations above the upper limit of normal 2 days after treatment. No moderate or severe elevations have been found. No difference in serum creatinine was found between subjects that were treated based on thick smear threshold or qPCR threshold. All subjects showed normalized values at the end of the study. An independent nephrology expert did not consider the transient decrease in renal function as a particular risk for permanent kidney damage.

Based on these findings the CHMI protocol has been adapted to:
- Exclude subjects with abnormal renal function at baseline.
- Avoid additional triggers that may cause renal dysfunction, such as:
  - Alcohol intake from baseline up to 1 week post-treatment (T3)
  - Heavy physical exercise
  - Use of recreational drugs
- Add standard monitoring of renal function at specific time points after CHMI

14.5. Risk management of reaction to the blood stage inoculum sample

The injection of *P. falciparum* 3D7 Master Cell Bank (MCB) inoculum contains a small number of red blood cells (between ~3.9x10^6 and 5.2x10^8) derived from the original donor. One mL of blood contains about 5 billion red cells. Thus, the prepared challenge inoculum would contain an extremely small amount of blood. The donor of the *P. falciparum* 3D7 MCB is also Blood Group O (Rh) negative and people with blood group O- blood are generally considered "universal donors", as recipients of their blood have minimal risk of developing red cell alloantibodies when given much larger volumes of blood than is envisaged here. The risk for development of red cell antibodies in this study is considered extremely low. However, it is possible that some of the volunteers could suffer a transfusion reaction after they receive the inoculum, or develop antibodies to the donor red blood cells that may make blood transfusion more difficult in the future. In a recently completed study (99), one volunteer developed an antibody response to a minor RH antigen (anti-E antibody) following the inoculation. Such allo-reactive antibodies have rarely been implicated in hemolytic transfusion reactions. There was no laboratory evidence to indicate that the specific Rh phenotype of the donor red cells in the inoculum provoked production of this allo anti-E antibody. Alloantibodies conversion following the inoculation has not been recorded in any of the other *P. falciparum* 3D7 MCB inoculum recipients. The onset of 'Natural' anti-E antibodies has also been described unrelated to transfusion. Nevertheless, the challenged volunteers will continue to be monitored after the administration of the inoculum to further assess the risk of this inoculum in causing transfusion reaction. Volunteers of

cohort B will also be tested for RBC alloantibodies at screening and at the end of the study as part of their safety monitoring. WOCBP will receive matched donor blood, i.e. Rh(c) and/or Rh(e) negative women will be excluded to participate in Cohort B, since the donor blood is Group O (Rh)D Negative ; C negative; E negative ; c positive ; e positive i.e. **rr** and Kell negative. Furthermore, participants with a history of blood transfusion are excluded to participate in these groups (section 9.3.17).

14.6. Synthesis

There is a large clinical experience with infecting humans by the bite of *P. falciparum*-infected mosquitoes and -infected erythrocytes. Since 1986 more than 3,500 volunteers have had CHMI by the bites of mosquitoes fed on cultures of *P. falciparum* gametocytes to produce sporozoites (28). This has proved to be a reproducible, predictable and safe method of inducing *P. falciparum* malaria. The results of such studies were summarized in 1997 (11), in 2007 (29) and in 2011 (30) (69). Following three cardiac events after blood stage parasitemia, our safety procedures for CHMI have been strongly intensified. In the current trial, we will use a 3D7 clone and adhere to those stringent procedures that are relevant, see section 1.4.

15. REFERENCES

1. WHO. Eliminating malaria. World Health Organization. 2016:24.
2. Ashley EA, Dhorda M, Fairhurst RM, Amaratunga C, Lim P, Suon S, et al. Spread of artemisinin resistance in Plasmodium falciparum malaria. The New England journal of medicine. 2014;371(5):411-23.
3. Sauerwein RW, Bousema T. Transmission blocking malaria vaccines: Assays and candidates in clinical development. Vaccine. 2015.
4. Mal ERACGoBS. Consultative Group on Vaccines. A research agenda for malaria eradication: vaccines. PLoS medicine. 2011;8(1):e1000398.
5. Adjalley SH, Johnston GL, Li T, Eastman RT, Ekland EH, Eappen AG, et al. Quantitative assessment of Plasmodium falciparum sexual development reveals potent transmission-blocking activity by methylene blue. Proceedings of the National Academy of Sciences of the United States of America. 2011;108(47):E1214-23.
6. Bhatt S, Weiss DJ, Cameron E, Bisanzio D, Mappin B, Dalrymple U, et al. The effect of malaria control on Plasmodium falciparum in Africa between 2000 and 2015. Nature. 2015;526(7572):207-11.
7. Hoffman SL, Vekemans J, Richie TL, Duffy PE. The March Toward Malaria Vaccines. American journal of preventive medicine. 2015;49(6S4):S319-S33.
8. Griffin JT, Hollingsworth TD, Okell LC, Churcher TS, White M, Hinsley W, et al. Reducing Plasmodium falciparum malaria transmission in Africa: a model-based evaluation of intervention strategies. PLoS medicine. 2010;7(8).
9. Targett GA, Greenwood BM. Malaria vaccines and their potential role in the elimination of malaria. Malaria journal. 2008;7 Suppl 1:S10.
10. Kiszewski AE. Blocking Plasmodium falciparum Malaria Transmission with Drugs: The Gametocytocidal and Sporontocidal Properties of Current and Prospective Antimalarials. Pharmaceuticals. 2010;4(1):44-68.
11. Church LW, Le TP, Bryan JP, Gordon DM, Edelman R, Fries L, et al. Clinical manifestations of Plasmodium falciparum malaria experimentally induced by mosquito challenge. The Journal of infectious diseases. 1997;175(4):915-20.
12. Epstein JE, Rao S, Williams F, Freilich D, Luke T, Sedegah M, et al. Safety and clinical outcome of experimental challenge of human volunteers with Plasmodium falciparum-infected mosquitoes: an update. The Journal of infectious diseases. 2007;196(1):145-54.
13. Roestenberg M, Teirlinck AC, McCall MB, Teelen K, Makamdop KN, Wiersma J, et al. Long-term protection against malaria after experimental sporozoite inoculation: an open-label follow-up study. Lancet. 2011;377(9779):1770-6.
14. Sauerwein RW, Roestenberg M, Moorthy VS. Experimental human challenge infections can accelerate clinical malaria vaccine development. Nature reviews Immunology. 2011;11(1):57-64.
15. Alving AS, Arnold J, Hockwald RS, Clayman CB, Dern RJ, Beutler E, et al. Potentiation of the curative action of primaquine in vivax malaria by quinine and chloroquine. The Journal of laboratory and clinical medicine. 1955;46(2):301-6.
16. Shapiro TA, Ranasinha CD, Kumar N, Barditch-Crovo P. Prophylactic activity of atovaquone against Plasmodium falciparum in humans. The American journal of tropical medicine and hygiene. 1999;60(5):831-6.
17. Berman JD, Nielsen R, Chulay JD, Dowler M, Kain KC, Kester KE, et al. Causal prophylactic efficacy of atovaquone-proguanil (Malarone) in a human challenge model. Transactions of the Royal Society of Tropical Medicine and Hygiene. 2001;95(4):429-32.
18. Beadle C, Long GW, Weiss WR, McElroy PD, Maret SM, Oloo AJ, et al. Diagnosis of malaria by detection of Plasmodium falciparum HRP-2 antigen with a rapid dipstick antigen-capture assay. Lancet. 1994;343(8897):564-8.
19. McCall MB, Netea MG, Hermsen CC, Jansen T, Jacobs L, Golenbock D, et al. Plasmodium falciparum infection causes proinflammatory priming of human TLR responses. Journal of immunology. 2007;179(1):162-71.

20. Teirlinck AC, McCall MB, Roestenberg M, Scholzen A, Woestenenk R, de Mast Q, et al. Longevity and composition of cellular immune responses following experimental Plasmodium falciparum malaria infection in humans. PLoS pathogens. 2011;7(12):e1002389.
21. Bousema T, Drakeley C. Epidemiology and infectivity of Plasmodium falciparum and Plasmodium vivax gametocytes in relation to malaria control and elimination. Clinical microbiology reviews. 2011;24(2):377-410.
22. Butcher GA. Antimalarial drugs and the mosquito transmission of Plasmodium. International journal for parasitology. 1997;27(9):975-87.
23. Sawa P, Shekalaghe SA, Drakeley CJ, Sutherland CJ, Mweresa CK, Baidjoe AY, et al. Malaria transmission after artemether-lumefantrine and dihydroartemisinin-piperaquine: a randomized trial. The Journal of infectious diseases. 2013;207(11):1637-45.
24. Bousema T, Churcher TS, Morlais I, Dinglasan RR. Can field-based mosquito feeding assays be used for evaluating transmission-blocking interventions? Trends in parasitology. 2013;29(2):53-9.
25. McCarthy J. Investigating the Controlled Human Malaria Infection model as a platform to evaluate transmission blocking interventions. American Society of Tropical Medicine and Hygiene Annual Meeting, New Orleans2014.
26. Laurens MB, Duncan CJ, Epstein JE, Hill AV, Komisar JL, Lyke KE, et al. A consultation on the optimization of controlled human malaria infection by mosquito bite for evaluation of candidate malaria vaccines. Vaccine. 2012;30(36):5302-4.
27. Engwerda CR, Minigo G, Amante FH, McCarthy JS. Experimentally induced blood stage malaria infection as a tool for clinical research. Trends in parasitology. 2012;28(11):515-21.
28. Chulay JD, Schneider I, Cosgriff TM, Hoffman SL, Ballou WR, Quakyi IA, et al. Malaria transmitted to humans by mosquitoes infected from cultured Plasmodium falciparum. The American journal of tropical medicine and hygiene. 1986;35(1):66-8.
29. Epstein JE, Giersing B, Mullen G, Moorthy V, Richie TL. Malaria vaccines: are we getting closer? Current opinion in molecular therapeutics. 2007;9(1):12-24.
30. Roestenberg M, O'Hara GA, Duncan CJ, Epstein JE, Edwards NJ, Scholzen A, et al. Comparison of clinical and parasitological data from controlled human malaria infection trials. PloS one. 2012;7(6):e38434.
31. Hermsen CC, de Vlas SJ, van Gemert GJ, Telgt DS, Verhage DF, Sauerwein RW. Testing vaccines in human experimental malaria: statistical analysis of parasitemia measured by a quantitative real-time polymerase chain reaction. The American journal of tropical medicine and hygiene. 2004;71(2):196-201.
32. Schneider P, Schoone G, Schallig H, Verhage D, Telgt D, Eling W, et al. Quantification of Plasmodium falciparum gametocytes in differential stages of development by quantitative nucleic acid sequence-based amplification. Molecular and biochemical parasitology. 2004;137(1):35-41.
33. Schneider P, Reece SE, van Schaijk BC, Bousema T, Lanke KH, Meaden CS, et al. Quantification of female and male Plasmodium falciparum gametocytes by reverse transcriptase quantitative PCR. Molecular and biochemical parasitology. 2015;199(1-2):29-33.
34. Mulder B, Lensen T, Tchuinkam T, Roeffen W, Verhave JP, Boudin C, et al. Plasmodium falciparum: membrane feeding assays and competition ELISAs for the measurement of transmission reduction in sera from Cameroon. Experimental parasitology. 1999;92(1):81-6.
35. Nieman AE, de Mast Q, Roestenberg M, Wiersma J, Pop G, Stalenhoef A, et al. Cardiac complication after experimental human malaria infection: a case report. Malaria journal. 2009;8:277.
36. van Meer MP, Bastiaens GJ, Boulaksil M, de Mast Q, Gunasekera A, Hoffman SL, et al. Idiopathic acute myocarditis during treatment for controlled human malaria infection: a case report. Malaria journal. 2014;13:38.
37. Marquart L, Baker M, O'Rourke P, McCarthy JS. Evaluating the pharmacodynamic effect of antimalarial drugs in clinical trials by quantitative PCR. Antimicrobial agents and chemotherapy. 2015;59(7):4249-59.

38. Falade CO, Yusuf BO, Fadero FF, Mokuolu OA, Hamer DH, Salako LA. Intermittent preventive treatment with sulphadoxine-pyrimethamine is effective in preventing maternal and placental malaria in Ibadan, south-western Nigeria. Malaria journal. 2007;6:88.
39. Peters PJ, Thigpen MC, Parise ME, Newman RD. Safety and toxicity of sulfadoxine/pyrimethamine: implications for malaria prevention in pregnancy using intermittent preventive treatment. Drug safety. 2007;30(6):481-501.
40. van Eijk AM, Ayisi JG, ter Kuile FO, Otieno JA, Misore AO, Odondi JO, et al. Effectiveness of intermittent preventive treatment with sulphadoxine-pyrimethamine for control of malaria in pregnancy in western Kenya: a hospital-based study. Tropical medicine & international health : TM & IH. 2004;9(3):351-60.
41. Zongo I, Milligan P, Compaore YD, Some AF, Greenwood B, Tarning J, et al. Randomized Noninferiority Trial of Dihydroartemisinin-Piperaquine Compared with Sulfadoxine-Pyrimethamine plus Amodiaquine for Seasonal Malaria Chemoprevention in Burkina Faso. Antimicrobial agents and chemotherapy. 2015;59(8):4387-96.
42. Roche. Fansidar product information. 2005.
43. Hogh B, Gamage-Mendis A, Butcher GA, Thompson R, Begtrup K, Mendis C, et al. The differing impact of chloroquine and pyrimethamine/sulfadoxine upon the infectivity of malaria species to the mosquito vector. The American journal of tropical medicine and hygiene. 1998;58(2):176-82.
44. Sowunmi A, Fateye BA, Adedeji AA, Fehintola FA, Bamgboye AE, Babalola CP, et al. Effects of antifolates--co-trimoxazole and pyrimethamine-sulfadoxine--on gametocytes in children with acute, symptomatic, uncomplicated, Plasmodium falciparum malaria. Memorias do Instituto Oswaldo Cruz. 2005;100(4):451-5.
45. Gargano N, Cenci F, Bassat Q. Antimalarial efficacy of piperaquine-based antimalarial combination therapies: facts and uncertainties. Tropical medicine & international health : TM & IH. 2011;16(12):1466-73.
46. Davis TM, Hung TY, Sim IK, Karunajeewa HA, Ilett KF. Piperaquine: a resurgent antimalarial drug. Drugs. 2005;65(1):75-87.
47. Nosten F, White NJ. Artemisinin-based combination treatment of falciparum malaria. The American journal of tropical medicine and hygiene. 2007;77(6 Suppl):181-92.
48. Karunajeewa HA, Ilett KF, Mueller I, Siba P, Law I, Page-Sharp M, et al. Pharmacokinetics and efficacy of piperaquine and chloroquine in Melanesian children with uncomplicated malaria. Antimicrobial agents and chemotherapy. 2008;52(1):237-43.
49. Myint HY, Ashley EA, Day NP, Nosten F, White NJ. Efficacy and safety of dihydroartemisinin-piperaquine. Transactions of the Royal Society of Tropical Medicine and Hygiene. 2007;101(9):858-66.
50. Group WARNWDS. The effect of dosing regimens on the antimalarial efficacy of dihydroartemisinin-piperaquine: a pooled analysis of individual patient data. PLoS medicine. 2013;10(12).
51. Sim IK, Davis TM, Ilett KF. Effects of a high-fat meal on the relative oral bioavailability of piperaquine. Antimicrobial agents and chemotherapy. 2005;49(6):2407-11.
52. Nguyen TC, Nguyen NQ, Nguyen XT, Bui D, Travers T, Edstein MD. Pharmacokinetics of the antimalarial drug piperaquine in healthy Vietnamese subjects. The American journal of tropical medicine and hygiene. 2008;79(4):620-3.
53. Price RN, Dorsey G, Nosten F. Antimalarial therapies in children from Papua New Guinea. The New England journal of medicine. 2009;360(12):1254; author reply 5.
54. Hai TN, Hietala SF, Van Huong N, Ashton M. The influence of food on the pharmacokinetics of piperaquine in healthy Vietnamese volunteers. Acta tropica. 2008;107(2):145-9.
55. Yeka A, Dorsey G, Kamya MR, Talisuna A, Lugemwa M, Rwakimari JB, et al. Artemether-lumefantrine versus dihydroartemisinin-piperaquine for treating uncomplicated malaria: a randomized trial to guide policy in Uganda. PloS one. 2008;3(6):e2390.
56. Kamya MR, Yeka A, Bukirwa H, Lugemwa M, Rwakimari JB, Staedke SG, et al. Artemether-lumefantrine versus dihydroartemisinin-piperaquine for treatment of malaria: a randomized trial. PLoS clinical trials. 2007;2(5):e20.
57. Nambozi M, Van Geertruyden JP, Hachizovu S, Chaponda M, Mukwamataba D, Mulenga M, et al. Safety and efficacy of dihydroartemisinin-piperaquine

versus artemether-lumefantrine in the treatment of uncomplicated Plasmodium falciparum malaria in Zambian children. Malaria journal. 2011;10:50.
58. Ratcliff A, Siswantoro H, Kenangalem E, Maristela R, Wuwung RM, Laihad F, et al. Two fixed-dose artemisinin combinations for drug-resistant falciparum and vivax malaria in Papua, Indonesia: an open-label randomised comparison. Lancet. 2007;369(9563):757-65.
59. Karunajeewa H, Lim C, Hung TY, Ilett KF, Denis MB, Socheat D, et al. Safety evaluation of fixed combination piperaquine plus dihydroartemisinin (Artekin) in Cambodian children and adults with malaria. British journal of clinical pharmacology. 2004;57(1):93-9.
60. Luntamo M, Kulmala T, Mbewe B, Cheung YB, Maleta K, Ashorn P. Effect of repeated treatment of pregnant women with sulfadoxine-pyrimethamine and azithromycin on preterm delivery in Malawi: a randomized controlled trial. The American journal of tropical medicine and hygiene. 2010;83(6):1212-20.
61. Maokola W, Chemba M, Hamisi Y, Mrisho M, Shirima K, Manzi F, et al. Safety of sulfadoxine/pyrimethamine for intermittent preventive treatment of malaria in infants: evidence from large-scale operational research in southern Tanzania. International health. 2011;3(3):154-9.
62. Mytton OT, Ashley EA, Peto L, Price RN, La Y, Hae R, et al. Electrocardiographic safety evaluation of dihydroartemisinin piperaquine in the treatment of uncomplicated falciparum malaria. The American journal of tropical medicine and hygiene. 2007;77(3):447-50.
63. EMA. Assessment Report Eurartesim. 2011.
64. EMA. Annex 1 Summary of Product Characteristics: Product Information for Eurartesim. 2011.
65. Targett G, Drakeley C, Jawara M, von Seidlein L, Coleman R, Deen J, et al. Artesunate reduces but does not prevent posttreatment transmission of Plasmodium falciparum to Anopheles gambiae. The Journal of infectious diseases. 2001;183(8):1254-9.
66. Hallett RL, Dunyo S, Ord R, Jawara M, Pinder M, Randall A, et al. Chloroquine/sulphadoxine-pyrimethamine for gambian children with malaria: transmission to mosquitoes of multidrug-resistant Plasmodium falciparum. PLoS clinical trials. 2006;1(3):e15.
67. Cheng Q, Lawrence G, Reed C, Stowers A, Ranford-Cartwright L, Creasey A, et al. Measurement of Plasmodium falciparum growth rates in vivo: a test of malaria vaccines. The American journal of tropical medicine and hygiene. 1997;57(4):495-500.
68. McCarthy JS, Sekuloski S, Griffin PM, Elliott S, Douglas N, Peatey C, et al. A pilot randomised trial of induced blood-stage Plasmodium falciparum infections in healthy volunteers for testing efficacy of new antimalarial drugs. PloS one. 2011;6(8):e21914.
69. Duncan CJ, Draper SJ. Controlled human blood stage malaria infection: current status and potential applications. The American journal of tropical medicine and hygiene. 2012;86(4):561-5.
70. Ouédraogo. A protocol for membrane feeding assays to determine the infectiousness of P. falciparum naturally infected individuals to Anopheles gambiae Malaria World Journal; 2013 [Available from: http://www.malariaworld.org/sites/default/files/mwjournal/article/MWJ%202013_4_16.pdf.
71. Blagborough AM, Churcher TS, Upton LM, Ghani AC, Gething PW, Sinden RE. Transmission-blocking interventions eliminate malaria from laboratory populations. Nature communications. 2013;4:1812.
72. Churcher TS, Blagborough AM, Delves M, Ramakrishnan C, Kapulu MC, Williams AR, et al. Measuring the blockade of malaria transmission--an analysis of the Standard Membrane Feeding Assay. International journal for parasitology. 2012;42(11):1037-44.
73. Stone WJ, Eldering M, van Gemert GJ, Lanke KH, Grignard L, van de Vegte-Bolmer MG, et al. The relevance and applicability of oocyst prevalence as a read-out for mosquito feeding assays. Scientific reports. 2013;3:3418.
74. Stone W, Grabias B, Lanke K, Zheng H, Locke E, Diallo D, et al. A comparison of Plasmodium falciparum circumsporozoite protein-based slot blot and ELISA immuno-assays for oocyst detection in mosquito homogenates. Malaria journal. 2015;14(1):451.

75. Wampfler R, Mwingira F, Javati S, Robinson L, Betuela I, Siba P, et al. Strategies for detection of Plasmodium species gametocytes. PloS one. 2013;8(9):e76316.
76. Tiburcio M, Dixon MW, Looker O, Younis SY, Tilley L, Alano P. Specific expression and export of the Plasmodium falciparum Gametocyte EXported Protein-5 marks the gametocyte ring stage. Malaria journal. 2015;14:334.
77. Joice R, Narasimhan V, Montgomery J, Sidhu AB, Oh K, Meyer E, et al. Inferring developmental stage composition from gene expression in human malaria. PLoS computational biology. 2013;9(12):e1003392.
78. Karl S, David M, Moore L, Grimberg BT, Michon P, Mueller I, et al. Enhanced detection of gametocytes by magnetic deposition microscopy predicts higher potential for Plasmodium falciparum transmission. Malaria journal. 2008;7:66.
79. Karl S, Davis TM, St-Pierre TG. A comparison of the sensitivities of detection of Plasmodium falciparum gametocytes by magnetic fractionation, thick blood film microscopy, and RT-PCR. Malaria journal. 2009;8:98.
80. de Mast Q, van Dongen-Lases EC, Swinkels DW, Nieman AE, Roestenberg M, Druilhe P, et al. Mild increases in serum hepcidin and interleukin-6 concentrations impair iron incorporation in haemoglobin during an experimental human malaria infection. British journal of haematology. 2009;145(5):657-64.
81. Hermsen CC, Konijnenberg Y, Mulder L, Loe C, van Deuren M, van der Meer JW, et al. Circulating concentrations of soluble granzyme A and B increase during natural and experimental Plasmodium falciparum infections. Clinical and experimental immunology. 2003;132(3):467-72.
82. Scholzen A, Sauerwein RW. Immune activation and induction of memory: lessons learned from controlled human malaria infection with Plasmodium falciparum. Parasitology. 2016;143(2):224-35.
83. Pabon A, Carmona J, Burgos LC, Blair S. Oxidative stress in patients with non-complicated malaria. Clin Biochem. 2003;36(1):71-8.
84. Plewes K, Kingston HWF, Ghose A, Maude RJ, Herdman MT, Leopold SJ, et al. Cell-free hemoglobin mediated oxidative stress is associated with acute kidney injury and renal replacement therapy in severe falciparum malaria: an observational study. BMC infectious diseases. 2017;17(1):313.
85. Guha M, Kumar S, Choubey V, Maity P, Bandyopadhyay U. Apoptosis in liver during malaria: role of oxidative stress and implication of mitochondrial pathway. FASEB journal : official publication of the Federation of American Societies for Experimental Biology. 2006;20(8):1224-6.
86. Dey S, Guha M, Alam A, Goyal M, Bindu S, Pal C, et al. Malarial infection develops mitochondrial pathology and mitochondrial oxidative stress to promote hepatocyte apoptosis. Free radical biology & medicine. 2009;46(2):271-81.
87. Jones S, Grignard L, Nebie I, Chilongola J, Dodoo D, Sauerwein R, et al. Naturally acquired antibody responses to recombinant Pfs230 and Pfs48/45 transmission blocking vaccine candidates. The Journal of infection. 2015;71(1):117-27.
88. Roestenberg M, de Vlas SJ, Nieman AE, Sauerwein RW, Hermsen CC. Efficacy of preerythrocytic and blood-stage malaria vaccines can be assessed in small sporozoite challenge trials in human volunteers. The Journal of infectious diseases. 2012;206(3):319-23.
89. Craig AG, Grau GE, Janse C, Kazura JW, Milner D, Barnwell JW, et al. The role of animal models for research on severe malaria. PLoS pathogens. 2012;8(2):e1002401.
90. Langhorne J, Buffet P, Galinski M, Good M, Harty J, Leroy D, et al. The relevance of non-human primate and rodent malaria models for humans. Malaria journal. 2011;10:23.
91. Gaur D, Chitnis CE. Molecular interactions and signaling mechanisms during erythrocyte invasion by malaria parasites. Current opinion in microbiology. 2011;14(4):422-8.
92. Organization WH. Guidelines for the treatment of malaria. Third edition2015.
93. UK G. Summary of Product Characteristics Malarone. Middlesex, UK2013. p. 8.

94. Pharma R. Drug prescription information Fansidar®. Reinach, Switserland2006.
95. (MMV) MfMV. INVESTIGATOR'S SUMMARY - Piperaquine phosphate. 2012.
96. Benjamin JM, Moore BR, Salman S, Page-Sharp M, Tawat S, Yadi G, et al. Population pharmacokinetics, tolerability, and safety of dihydroartemisinin-piperaquine and sulfadoxine-pyrimethamine-piperaquine in pregnant and nonpregnant Papua New Guinean women. Antimicrobial agents and chemotherapy. 2015;59(7):4260-71.
97. Cisse B, Cairns M, Faye E, O ND, Faye B, Cames C, et al. Randomized trial of piperaquine with sulfadoxine-pyrimethamine or dihydroartemisinin for malaria intermittent preventive treatment in children. PloS one. 2009;4(9):e7164.
98. World Health O. World Malaria Report. 2014.
99. Krause A, Dingemanse J, Mathis A, Marquart L, Mohrle JJ, McCarthy JS. Pharmacokinetic/pharmacodynamic modelling of the antimalarial effect of Actelion-451840 in an induced blood stage malaria study in healthy subjects. British journal of clinical pharmacology. 2016;82(2):412-21.

16. APPENDICES

16.1. Appendix 1

Clinical Score for Malaria (Appendix 1)

Clinical Score	
**Symptom**	**Clinical Score**<br>*0 - Absent, 1 - Mild, 2 - Moderate, 3 – Severe*
Headache	
Myalgia (muscle ache)	
Arthralgia (joint ache)	
Fatigue/lethargy	
Malaise (general discomfort/uneasiness)	
Chills/shivering/rigors	
Sweating/hot spells	
Anorexia	
Nausea	
Vomiting	
Abdominal discomfort	
Fever	
**TOTAL SCORE**	

Recorder's signature: ____ Date: ________________
