# Supplementary material for: Controlled human malaria infections by mosquito bites induce more severe clinical symptoms than asexual blood-stage challenge infections
Source: eBioMedicine. 2022 Mar 9;77:103919. doi: 10.1016/j.ebiom.2022.103919 (PMC8917304; doi:10.1016/j.ebiom.2022.103919)
Supplement: Supplementary file 3 [file mmc3.docx]

**Supplementary material**

**Clinical trial protocol**
